# The effect of glucocorticoid therapy on mortality in patients with rheumatoid arthritis and concomitant type II diabetes: a retrospective cohort study

**DOI:** 10.1186/s41927-019-0105-4

**Published:** 2020-02-19

**Authors:** Ruth E. Costello, Antonia Marsden, Mohammad Movahedi, Mark Lunt, Jenny H. Humphreys, Richard Emsley, William G. Dixon

**Affiliations:** 1 Centre for Epidemiology Versus Arthritis, Centre for Musculoskeletal Research, Faculty of Biology, Medicine and Health, Manchester Academic Health Science Centre, The University of Manchester, Manchester, UK; 2 Centre for Biostatistics, School of Health Sciences, The University of Manchester, Manchester Academic Health Science Centre, Manchester, UK; 3 Ontario Best Practices Research Initiative, University Health Network, Toronto, Ontario Canada; 4 Institute of Psychiatry, Psychology and Neuroscience, King’s College London, London, UK

**Keywords:** Rheumatology, General diabetes, Epidemiology

## Abstract

**Background:**

Patients with rheumatoid arthritis (RA) have increased cardiovascular (CV) and mortality risk. Patients with RA are also frequently prescribed glucocorticoids (GCs) which have been associated with increased risk of mortality. In addition, for patients who have concomitant diabetes mellitus (DM), GCs are known to worsen glycaemic control and hence may further increase CV and mortality risk. This study aimed to understand the relationship between GCs, DM and mortality in patients with RA.

**Methods:**

This was a retrospective cohort study of patients with incident RA identified from UK primary care electronic medical records. Patients with linkage to Office for National Statistics (ONS) for mortality data (*N* = 9085) were included. DM was identified through Read codes, prescriptions and blood tests, and GC use was identified through prescriptions. Mortality rate ratios (RR) and rate differences (RD) were calculated across the different exposure groups. Cox proportional hazards regression models were used to estimate interaction on the multiplicative and additive scales.

**Results:**

In those without DM GC use had a 4.4-fold increased all-cause mortality RR (95% confidence interval (CI): 3.77 to 5.07) compared to non-use, whilst those with DM had a lower RR for GC use (2.99 (95% CI: 2.32, 3.87)). However, those with DM had a higher RD associated with GC use because of their higher baseline risk. In those with DM, GC use was associated with an additional 44.9 deaths/1000 person-years (pyrs) (95% CI: 32.9 to 56.8) compared to non-use, while in those without DM GC use was associated with an additional 34.4 deaths/1000 pyrs (95% CI: 30.1 to 38.7) compared to non-use, while in those without DM GC use was associated with an additional 36.2 deaths/1000 pyrs (95% CI: 31.6 to 40.8). A similar pattern was seen for CV mortality. The adjusted Cox proportional hazards model showed no evidence of multiplicative interaction, but additive interaction indicated a non-significant increased risk. For CV mortality there was no interaction on either scale.

**Conclusions:**

GC use was associated with higher mortality rates in people with comorbid DM compared to people without DM, despite apparently reassuring similar relative risks. Clinicians need to be aware of the higher baseline risk in patients with DM, and consider this when prescribing GCs in patients with RA and comorbid DM.

## Background

Rheumatoid arthritis (RA) is an inflammatory disease that is thought to affect around 1% of the UK population [1] and is associated with a significantly higher rate of cardiovascular (CV) mortality compared to the general population [2]. Glucocorticoids (GC) have been widely used as a treatment for RA since their discovery in the 1950s [3] and continue to be used in around half of patients with RA [4]. Although GCs have many benefits, they also have risks associated with them, including possible increased risk of CV events and mortality [5, 6]. In addition, GCs are known to increase the risk of diabetes mellitus (DM) [7, 8] and are associated with poor glucose control [9], meaning they may also affect the long-term outcome of DM (including CV events and mortality) [10, 11]. This has not been investigated in patients with RA. Further, it is not known how the additional burden of DM and then GC therapy influence the cardiovascular and mortality risk in patients with RA. Therefore an important unanswered question is whether GC treatment in RA is associated with worse outcomes in patients with comorbid DM, compared to patients without DM.

As we think that the baseline risk of CV and all-cause mortality for patients with RA and DM will be higher than those with RA only, to investigate the impact of GCs it is appropriate to look at the absolute risks as well as the relative risks. The aims of this study were: 1) to compare the event rates for all-cause mortality and CV mortality, by GC use status and DM status, and 2) to examine whether DM modifies, on either the multiplicative or additive scales, the effect of GCs on all-cause mortality and CV mortality.

## Methods

### Setting

This was a retrospective cohort study using data from the Clinical Practice Research Datalink (CPRD) which was linked to mortality data from the Office of National Statistics (ONS). The CPRD is a large database of primary care electronic medical records that covers around 7% of the UK population and has been shown to be broadly representative of the UK population. Consenting practices in England have linkage to the ONS mortality data, which represents around 58% of all CPRD practices [12]. CPRD provide indicators of when a practice’s data was up to research standard, and whether a patient’s data meets their acceptability standards. For this study, only data from practices that consented to ONS linkage were used if the data met acceptability standards and was up to research standard.

### Study population

The study period began at the start of ONS coverage (1st January 1998) and ended 1st October 2011. Patients with incident RA during the study period were identified from CPRD using a validated algorithm where patients have to have either at least 2 Read codes for RA and no alternative diagnosis after their last RA code or a Read code for RA and at least 2 product (medication) codes for Disease-Modifying Anti-Rheumatic Drugs (DMARDs) and no alternative diagnosis for the DMARDs in the previous 5 years [13]. Patients entered into the study upon RA diagnosis and participation ended at death, the date the patient left the practice or at the end of the study period. All patients were registered with the practice for a year prior to RA diagnosis, to ensure patients were truly incident cases.

### Exposures

Patients were identified as having type 2 DM if they had either (1) a Read code for type 2 DM; (2) at least two prescriptions for oral anti-diabetic medication, either on 2 different dates or the same date with 2 types of medication; or (3) fasting blood sugar ≥7.0 mmol/litre, random glucose test ≥11.1 mmol/litre, glucose tolerance test ≥11.1 mmol/litre or a glycosylated haemoglobin (HbA1C) ≥7% [7]. Patients with polycystic ovary syndrome (PCOS) treated with metformin were excluded as it was possible they were incorrectly identified as diabetic because of taking anti-diabetic medication. Diagnosis of DM was time-varying and could be prior to diagnosis of RA whereby a person would be flagged as diabetic throughout follow-up, or during follow-up whereby a person would be flagged as diabetic from the point of DM diagnosis. Where the diagnosis was made on the basis of two sequential prescriptions, the date of onset was allocated as the date of the second prescription to avoid immortal time bias.

Oral GC therapy was identified using product codes from prescription data. Patients were classified by current/recent use of GCs, whereby a person was classified as exposed for the duration of each GC prescription and for 6 months after the end of the prescription.

### Outcomes

All-cause and CV mortality were identified through linkage to ONS data with date of death and cause of death provided. Cause of death was recorded on ONS using International Statistical Classification of Diseases and Related Health Problems (ICD) version 10 codes. Deaths prior to 2001 were recorded using ICD-9 codes and these were mapped to ICD-10 codes. There also were 31 deaths recorded on CPRD but not on ONS and these were included in the all-cause mortality analyses. CV mortality was identified using ICD-10 codes under the circulatory chapter heading as the underlying cause of death.

### Covariates

Age at RA diagnosis was calculated using year of birth and year of RA diagnosis. Gender was given on the CPRD database. Baseline Charlson comorbidity index was determined using an adaption of the index for CPRD data where diseases were identified through Read codes for diagnosis at any point prior to RA diagnosis [14]. DMARD types and non-steroidal anti-inflammatory drugs (NSAIDs) were identified using product codes and were time-varying. GC use in the year preceding baseline was determined from GC prescriptions prior to baseline. Baseline smoking category (ever or never) was determined using Read codes and product codes at any point up to RA diagnosis, or in the 3 months after RA diagnosis. Prior macrovascular disease was defined as diseases of large blood vessels including myocardial infarction, stroke, peripheral artery disease or amputation [15] and were identified through Read codes prior to RA diagnosis. Body mass index (BMI) at baseline was calculated using median height and weight measurements from the 5 years prior to baseline. All code lists can be found in Additional file 1.

### Analysis

For both outcomes, mortality rates were estimated (with 95% confidence intervals (CI)), stratified by time-varying DM status and time-varying current/recent use of GCs. As mentioned earlier, the baseline risk of CV and all-cause mortality for patients with RA and DM will be higher than those with RA only. Therefore, to investigate the impact of GCs both rate ratios (RR) and rate differences (RD) between GC users and non-GC users were calculated for those with and without DM separately.

When estimating the effect of both GC exposure and DM status, the presence of interaction was measured on both the multiplicative scale, corresponding to the RR, and on the additive scale, corresponding to the RD. Interaction on the additive scale can give more meaningful comparisons as it is not dependent on baseline risks [16]. Crude and adjusted Cox proportional hazards (PH) regression models were fitted with an interaction term for time-varying DM and time-varying current/recent use of GCs. Multiplicative interaction was assessed via the inclusion of an interaction term in the Cox model.

Additive interaction cannot be estimated directly from the Cox model as it depends on the baseline hazard function [17]. However, we can estimate the Relative Excess Risk due to Interaction (RERI) and Ratio of Absolute Effects (RAE): 1) RERI [17, 18] assesses if there is a difference in the hazard differences. The RERI is equal to 0 if the additive interaction effect is equal to 0. Therefore, if it is statistically significantly different from zero then this is interpreted as a statistically significant difference in the hazard differences between those with and without DM, and indicates the direction of the effect. 2) RAE is defined as the ratio of hazard differences in patients with DM compared to those without DM (See Additional file 2 for further information). Departure from 1 indicates a difference in the two groups and it was calculated here in addition to the RERI as it gives an indication of the magnitude of the difference in subgroup absolute effects, unlike the RERI. Both measures are calculated after the Cox model as a function of the model parameters.

### Missing data

Ever smoking at baseline and baseline BMI had 753 (8%) and 3849 (42%) missing data, respectively. Multiple imputation with 57 imputations was used to replace these missing values. The number of imputations was based on the fraction of missing information. Forty-nine patients did not have a Townsend score, however this was not imputed as it was not used in the final models.

## Results

**Fig. 1:**
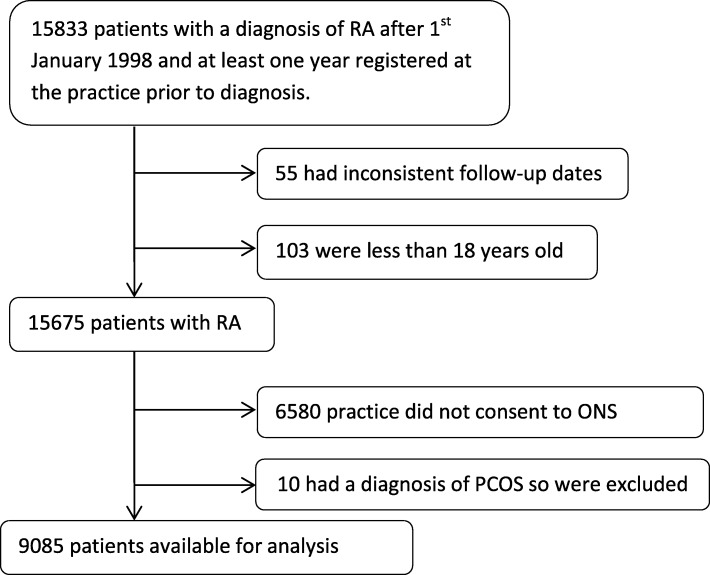
Flowchart of patients selected for the study

There were 15,833 patients identified who had a diagnosis of RA and were registered at their practice for at least 1 year prior to diagnosis, 6748 were excluded due to either inconsistent follow-up dates, being age 18 years or under at diagnosis, being registered at a practice that did not consent to ONS linkage or having a diagnosis of PCOS and being treated with metformin, resulting in 9085 patients in the final cohort (Fig. 1). The cohort had a mean follow-up of 5.2 years (standard deviation 3.5 years).

At baseline there were 1034 patients with DM, and 761 patients developed DM during follow-up. Compared to those without DM at baseline, those with DM at baseline were older (DM: mean 64 years vs non-DM: mean 59 years) had a greater proportion of males (DM: 37% vs non-DM: 30%) and ever smokers (DM: 58% vs non-DM: 50%), had more GC use prior to baseline (DM: 31% vs non-DM: 23%), had more macrovascular disease at baseline (DM: 11% vs non-DM: 4%) and had a higher BMI (DM: 30 vs non-DM: 27) (Table 1). 50% of patients had used GC at any point during follow-up. Those with prior DM had slightly higher average GC dose over follow-up (DM: 4.9 mg prednisolone equivalent dose (PED) vs non-DM: 4.4 mg PED). Across both those with and without DM those who ever used GC were older and had more prior macrovascular disease.

**Table 1:** Baseline characteristics by diabetes mellitus status and ever use of glucocorticoids during follow-up (*N* = 9085)

	DM at baseline			No DM at baseline			DM during FU^a^		
	All subjects	Never users	Ever users	All subjects	Never users	Ever users	All subjects	Never users	Ever users
	*N* = 1034	*N* = 512	*N* = 522	*N* = 8051	*N* = 4026	*N* = 4025	*N* = 761	*N* = 269	*N* = 492
Females, n (%)	652 (63.1)	325 (63.5)	327 (62.6)	5600 (69.6)	2878 (71.5)	2722 (67.6)	503 (66.1)	176 (65.4)	327 (66.5)
Age at baseline (years), mean (standard deviation (SD))	64.42 (13.0)	63 13.6)	65.81 (12.3)	58.51 (14.7)	55.94 (14.6)	61.07 (14.4)	64.63 (12.8)	62.4 (12.9)	65.86 (12.5)
Body Mass Index in year prior to baseline									
mean (SD)	29.77 (6.5)	29.78 (6.7)	29.77 (6.3)	27.14 (5.5)	27.17 (5.5)	27.1 (5.4)	29.86 (6.9)	30.71 (6.8)	29.35 (6.9)
Missing (%)	180 (17.4)	87 (17.0)	93 (17.8)	3669 (45.6)	1853 (46.0)	1816 (45.1)	224 (29.4)	68 (25.3)	156 (31.7)
Smoking status at baseline, n (%)									
Never smoker	407 (39.4)	203 (39.7)	204 (39.1)	3337 (41.5)	1748 (43.4)	1589 (39.5)	234 (30.8)	80 (29.7)	154 (31.3)
Ever smoker	600 (58.0)	293 (57.2)	307 (58.8)	3988 (49.5)	1949 (48.4)	2039 (50.7)	505 (66.4)	182 (67.7)	323 (65.7)
Missing	2 (0.2)	1 (0.2)	1 (0.2)	41 (0.5)	20 (0.5)	21 (0.5)	5 (0.7)	1 (0.4)	4 (0.8)
SES quintile at baseline (In subset), n (%)									
First (least deprived)	214 (20.7)	102 (19.9)	112 (21.5)	1830 (22.7)	910 (22.6)	920 (22.9)	165 (21.7)	60 (22.3)	105 (21.3)
Second	224 (21.7)	98 (19.1)	126 (24.1)	1993 (24.8)	1023 (25.4)	970 (24.1)	182 (23.9)	65 (24.2)	117 (23.8)
Third	215 (20.8)	121 (23.6)	94 (18.0)	1731 (21.5)	839 (20.8)	892 (22.2)	156 (20.5)	62 (23.1)	94 (19.1)
Fourth	229 (22.2)	119 (23.2)	110 (21.1)	1470 (18.3)	741 (18.4)	729 (18.1)	161 (21.2)	49 (18.2)	112 (22.8)
Fifth (most deprived)	150 (14.5)	71 (13.9)	79 (15.1)	986 (12.3)	493 (12.3)	493 (12.3)	92 (12.1)	32 (11.9)	60 (12.2)
Missing	2 (0.2)	1 (0.2)	1 (0.2)	41 (0.5)	20 (0.5)	21 (0.5)	6 (0.8)	1 (0.4)	5 (1.0)
Charlson comorbidity index at baseline, mean (SD)	2.57 (0.8)	2.48 (0.8)	2.66 (0.8)	1.32 (0.7)	1.23 (0.6)	1.42 (0.7)	2.63 (0.8)	2.39 (0.7)	2.76 (0.8)
Prior history of macrovascular diseases, n (%)	113 (10.9)	41 (8.0)	72 (13.8)	297 (3.7)	108 (2.7)	189 (4.7)	77 (10.1)	16 (6.0)	61 (12.4)
History of GC use in year prior to baseline, n (%)	325 (31.4)	42 (8.2)	283 (54.2)	1861 (23.1)	256 (6.4)	1605 (39.9)	299 (39.3)	3 (1.1)	296 (60.2)
Duration of diabetes at baseline (yrs)									
Mean (SD)	4.59 (3.7)	4.88 (3.8)	4.31 (3.6)	N/A	N/A	N/A	N/A	N/A	N/A
Number of anti-DM medication prior to baseline									
0	525 (50.8)	264 (50.6)	261 (51.0)	N/A	N/A	N/A	N/A	N/A	N/A
1	482 (46.6)	249 (47.7)	233 (45.5)	N/A	N/A	N/A	N/A	N/A	N/A
2	26 (2.5)	8 (1.5)	18 (3.5)	N/A	N/A	N/A	N/A	N/A	N/A
3	1 (0.1)	1 (0.2)	0 (0)	N/A	N/A	N/A	N/A	N/A	N/A
Prescribed insulin prior to follow-up, n (%)	155 (15.0)	86 (16.8)	69 (13.2)	N/A	N/A	N/A	N/A	N/A	N/A
DMARDs prescribed during follow-up, n (%)									
Methotrexate	633 (61.2)	309 (60.4)	324 (62.1)	5012 (62.3)	2386 (59.3)	2626 (65.2)	353 (46.4)	110 (40.9)	243 (49.4)
Hydroxychloroquine	261 (25.2)	132 (25.8)	129 (24.7)	2304 (28.6)	1129 (28.0)	1175 (29.2)	138 (18.1)	49 (18.2)	89 (18.1)
Sulfasalazine	372 (36.0)	171 (33.4)	201 (38.5)	3333 (41.4)	1591 (39.5)	1742 (43.3)	207 (27.2)	80 (29.7)	127 (25.8)
Leflunomide	75 (7.3)	28 (5.5)	47 (9.0)	719 (8.9)	264 (6.6)	455 (11.3)	57 (7.5)	13 (4.8)	44 (8.9)
Other	70 (6.8)	15 (2.9)	55 (10.5)	549 (6.8)	117 (2.9)	432 (10.7)	54 (7.1)	6 (2.2)	48 (9.8)
Average GC dose during follow up, mean (SD)	4.93 (14.0)	0	9.8 (18.5)	4.38 (6.3)	0	8.75 (6.4)	5.02 (6.3)	0	7.77 (6.4)

^a^Characteristics at time of diabetes mellitus diagnosis

### All-cause mortality

During follow-up there were 1,005 deaths. Mortality rates differed according to the presence of DM and the use of GC therapy. For those with DM, the mortality rate was 67.4 (95% CI 57.1 to 79.5) per 1000 person-years (pyrs) in those with GC exposure and 22.5 (95% CI 18.7 to 27.1) per 1000 pyrs in those without GC exposure. For those without DM, the mortality rate was 44.6 (95% CI 40.6 to 48.9) per 1000 pyrs in those exposed to GCs and 10.2 (95% CI: 9.1 to 11.4) per 1000 pyrs in those without GC exposure. The risk ratio for GC use was slightly lower for those with DM (DM RR 2.99 (95% confidence interval (CI) 2.32 to3.87) compared to those with no DM RR 4.37 (95% CI 3.77 to 5.07)). However, despite this lower RR, those with DM had a *higher RD* compared to those without DM (DM RD: 44.9 (95% CI: 32.9 to 56.8) vs no DM RD: 34.4 (95% CI: 30.1 to 38.7 per 1000 pyrs) (Table 2).

**Table 2:**
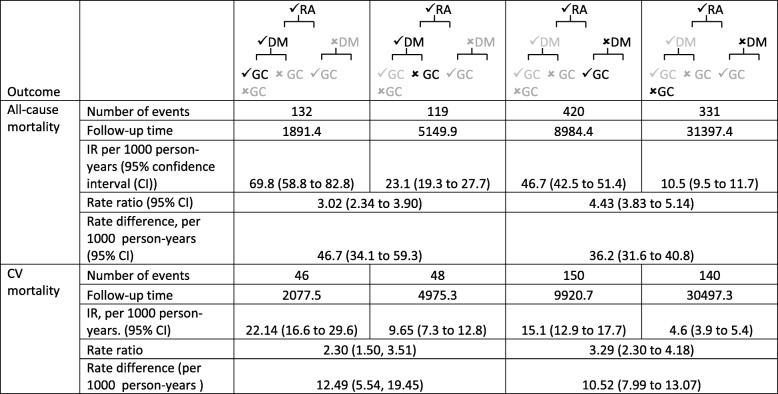
Mortality rates, rate ratios and rate difference by diabetes mellitus and glucocorticoid use status

The unadjusted Cox PH model for all-cause mortality showed current/recent GC use and DM interacted on the multiplicative scale (0.69 (95% CI 0.51, 0.91)). Adjustment removed this significant interaction (0.86 (95% CI: 0.64–1.15)) (Table 3). In both the unadjusted and adjusted models both the RERI and RAE indicated increased risk for those with DM and current/recent GC use but were not statistically significant (adjusted RAE: 1.22 (95% CI: 0.86 to 1.72) (Table 3).

**Table 3:** Multiplicative and additive interaction^a^ between diabetes mellitus and ever glucocorticoid use

Outcome	Multiplicative interaction	Additive interaction	
	Hazard ratio (95% confidence interval)	RERI (95% confidence interval)	RAE (95% confidence interval)
All-cause mortality			
Unadjusted	0.69 (0.51 to 0.91)	0.94 (−0.29 to 2.16)	1.27 (0.95 to 1.70)
All-cause mortality			
Adjusted^b^	0.86 (0.64 to 1.15)	0.41 (−0.36 to 1.18)	1.22 (0.86 to 1.72)
CV mortality			
Unadjusted	0.70 (0.44 to 1.11)	0.40 (−1.19 to 2.00)	1.17 (0.64 to 2.14)
CV mortality			
Adjusted^b^	0.93 (0.60 to 1.48)	0.11 (−0.75 to 0.96)	1.11 (0.48 to 2.57)

^a^ On the multiplicative scale significant interaction is different than 1, on the additive scale significant interaction for the RERI is different than 0 and for the RAE is different than 1

^b^Adjusted for age, gender, Charlson comorbidity index, baseline BMI, baseline smoking status, DMARDs, prior GC, prior macrovascular disease and NSAIDs

### CV mortality

There were 384 CV deaths during follow-up. A similar pattern was seen for CV mortality, where a slightly lower RR was seen for those with DM compared to those without DM, but the RD was higher for those with DM (Table 2). The unadjusted and adjusted Cox models showed that DM did not interact with ever GC use on the multiplicative scale, the additive interaction indicated increased risk but was not statistically significant (Table 3).

## Discussion

In this study, we have shown that in patients with RA and DM, the RR of GC use on all-cause and CV mortality was slightly lower than in patients with RA alone. This might seem reassuring at first glance, suggesting the impact of GC therapy in patients with DM is no worse than in patients without DM. However, the RD was notably higher in those with DM compared to those without. The higher baseline mortality rate for those with DM is thus resulting in a greater number of excess deaths despite the slightly lower RR. When examined together in an adjusted Cox PH model, current/recent use of GC in those with DM was associated with a non-significant absolute increased hazard of all-cause mortality compared to those without DM, but not a relative increased hazard. A similar pattern was seen for CV mortality. The increased absolute hazard for all-cause mortality indicates the greater public health impact of people with RA using GCs if they have DM. This increase is not seen on the multiplicative scale because the comparison made is relative to other patients with DM who have a higher risk of mortality prior to using GCs. Notably, most studies only assess effect modification or interaction on the multiplicative scale, despite recommendations to use both the multiplicative and additive scales [16, 19].

To our knowledge no previous studies have looked at the effect of both GCs and DM on mortality in patients with RA. Studies have looked at short term diabetic outcomes with GC use, investigating its effects on glucose intolerance or metabolic syndrome in patients with RA [8, 20]. Two studies have investigated longer term outcomes of GC use in patients with DM but not RA. One looked at mortality 14 years after diagnosis and found that after adjustment for age and gender there was not increased mortality in patients with DM who had GC treatment compared to those who did not, however only small numbers of patients had GC treatment in this study (35/1334) [10]. The other study aimed to describe the adverse effects of GC treatment in patients with DM, but did not discuss mortality [11]. We and others have previously shown GC therapy to be associated with higher all-cause mortality rates in patients with RA. However, a causal association is difficult to establish as several biases are at play in an observational study including ‘peri-mortal bias’ [21].

This was a large study that used electronic medical records that are a rich source of medical information. CPRD data has been shown to be broadly representative of the UK population, so results should be generalisable to the UK RA population [12]. However, there are some limitations with the study. Although we used a validated algorithm to identify patients with RA there could still be some misclassification. Further misclassification may result from medication being based on prescription data rather than dispensing data. However, any differences between prescribed medication and medication dispensed are unlikely to differ by DM status. To allow examination of interaction a simple model of oral GC exposure was used, therefore it was not possible to examine the impact of GC dose or intramuscular GCs. This study focuses on type 2 DM, as GCs induce insulin resistance similar to type 2 diabetes. Results are likely to be similar with type 1 diabetes, but given the different pathogenetic mechanisms, further work would be required to confirm this. There could be confounding by indication, as RA disease severity has been shown to confound the relationship between GCs and CVD in RA [22]. However, there is no measure of disease activity available on CPRD and we would not expect the confounding to differentially affect those with or without DM. There may be known unmeasured confounding, there were no measures of biologic DMARD use in this study as biologics are only prescribed in secondary care in the UK. This may be important as biologics have been shown to be associated with reduced CVD [23]. Unfortunately we were not able to use methods to explore unmeasured confounding as most are applied to relative risks rather than additive interaction terms.

## Conclusions

This study gives an indication that GC therapy may be associated with a higher number of deaths in patients with RA and comorbid type 2 DM. Rheumatologists should consider DM status when prescribing GCs to patients with RA given this potential impact of GC therapy on glucose control and mortality.

## Supplementary information

**Additional file 1.** Codelists for disease and drug definition.

**Additional file 2.** The RAE measure.

## Data Availability

Clinical Practice Research Datalink (CPRD) data can be accessed with an appropriate licence from the CPRD and with approval from the Independent Scientific Advisory Committee. Licences are available from CPRD: Clinical Practice Research Datalink, The Medicines and Healthcare products Regulatory Agency, 10th Floor, 10 South Colonnade, Canary Wharf, London E14 4PU, England or http://www.cprd.com.

## Abbreviations

BMI: Body mass index
CI: Confidence interval
CPRD: Clinical practice research datalink
CV: Cardiovascular
DM: Diabetes mellitus
DMARDs: Disease modifying anti-rheumatic drugs
GCs: Glucocorticoids
Hba1c: Glycosylated haemoglobin
ICD: International Statistical Classification of Diseases and Related Health Problems
NSAIDs: Non-steroidal anti-inflammatory drugs
ONS: Office for National Statistics
PCOS: Polycystic ovary syndrome
pyrs: Person years
RA: Rheumatoid arthritis
RAE: Ratio of Absolute Effects
RD: Rate difference
RERI: Relative Excess Risk due to Interaction
RR: Rate ratio
