# Correction to: The effect of glucocorticoid therapy on mortality in patients with rheumatoid arthritis and concomitant type II diabetes: a retrospective cohort study

**DOI:** 10.1186/s41927-021-00181-8

**Published:** 2021-03-02

**Authors:** Ruth E. Costello, Antonia Marsden, Mohammad Movahedi, Mark Lunt, Jenny H. Humphreys, Richard Emsley, William G. Dixon

**Affiliations:** 1 Centre for Epidemiology Versus Arthritis, Centre for Musculoskeletal Research, Faculty of Biology, Medicine and Health, Manchester Academic Health Science Centre, The University of Manchester, Manchester, UK; 2 Centre for Biostatistics, School of Health Sciences, The University of Manchester, Manchester Academic Health Science Centre, Manchester, UK; 3 Ontario Best Practices Research Initiative, University Health Network, Toronto, Ontario Canada; 4 Institute of Psychiatry, Psychology and Neuroscience, King’s College London, London, UK

**Correction to: BMC Rheumatol 4, 4 (2020)**

**https://doi.org/10.1186/s41927-019-0105-4**

Following publication of the original article [1], the authors noted several errors in the reported values in Table 2, the ‘Results’ section of the abstract, and in the first sentence of the “All-cause mortality” sub-section. The correct Table and text are given below with the corrected values highlighted in bold for the Abstract.

The original article has been updated.

**Abstract**

**Results:** In those without DM GC use had a 4.4-fold increased all-cause mortality RR (95% confidence interval (CI): **3.77 to 5.07**) compared to non-use, whilst those with DM had a lower RR for GC use (**2.99 (95% CI: 2.32, 3.87**)). However, those with DM had a higher RD associated with GC use because of their higher baseline risk. In those with DM, GC use was associated with an additional **44.9** deaths/1000 person-years (pyrs) (95% CI: **32.9 to 56.8**) compared to non-use, while in those without DM GC use was associated with an additional **34.4** deaths/1000 pyrs (95% CI: **30.1 to 38.7**). A similar pattern was seen for CV mortality. The adjusted Cox proportional hazards model showed no evidence of multiplicative interaction, but additive interaction indicated a non-significant increased risk. For CV mortality there was no interaction on either scale.

**Table 2:**
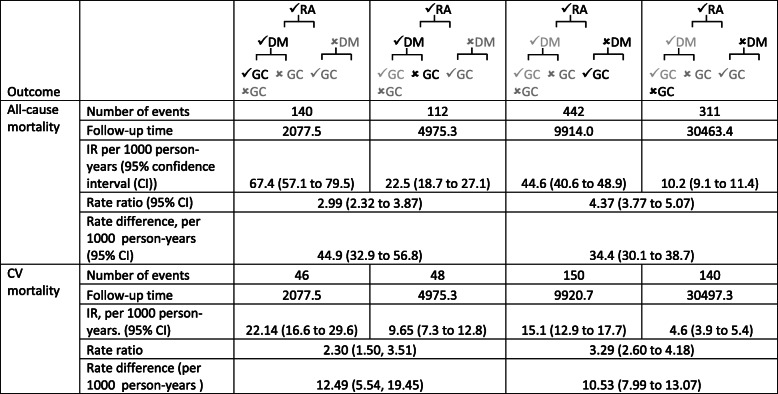
Mortality rates, rate ratios and rate difference by diabetes mellitus and glucocorticoid use status

In the “All-cause mortality” sub-section it now correctly reads “During follow-up there were 1005 deaths” rather than 1002 deaths.
