# Supplementary material for: The effect of glucocorticoid therapy on mortality in patients with rheumatoid arthritis and concomitant type II diabetes: a retrospective cohort study
Source: BMC Rheumatol. 2020 Feb 19;4:4. doi: 10.1186/s41927-019-0105-4 (PMC7029556; doi:10.1186/s41927-019-0105-4)
Supplement: Supplementary file 1 — Additional file 1. Codelists for disease and drug definition. [file 41927_2019_105_MOESM1_ESM.docx]

## Codelists for disease and drug definitions

Rheumatoid arthritis - medcodes

| Medcode | Description |
| --- | --- |
| 844 | Rheumatoid arthritis |
| 5723 | Rheumatoid nodule |
| 6639 | H/O: rheumatoid arthritis |
| 6916 | Seronegative rheumatoid arthritis |
| 8350 | Flare of rheumatoid arthritis |
| 9707 | Seropositive errosive rheumatoid arthritis |
| 9954 | Rheumatoid lung |
| 12019 | Seropositive rheumatoid arthritis, unspecified |
| 17412 | Rheumatoid arthrit. monitoring |
| 21358 | Rheumatoid arthritis of shoulder |
| 23552 | Felty's syndrome |
| 23834 | Adult Still's Disease |
| 27603 | Rheumatoid arthritis and other inflammatory polyarthropathy |
| 28853 | Fibrosing alveolitis associated with rheumatoid arthritis |
| 30548 | Rheumatoid vasculitis |
| 31054 | Rheumatoid arthritis - multiple joint |
| 31209 | Myopathy due to rheumatoid arthritis |
| 31724 | Rheumatoid lung |
| 32001 | Adult-onset Still's disease |
| 37431 | Rheumatoid arthropathy + visceral/systemic involvement NOS |
| 41941 | Rheumatoid arthritis of PIP joint of finger |
| 42299 | Rheumatoid arthritis of MCP joint |
| 43816 | Rheumatoid carditis |
| 44203 | Other rheumatoid arthritis of spine |
| 44743 | Rheumatoid arthritis of cervical spine |
| 46436 | Rheumatoid lung disease |
| 48832 | Rheumatoid arthritis of wrist |
| 49067 | Rheumatoid arthritis of hip |
| 49227 | Other rheumatoid arthropathy + visceral/systemic involvement |
| 49787 | Rheumatoid myocarditis |
| 50863 | Rheumatoid arthritis of knee |
| 51238 | Rheumatoid arthritis of 1st MTP joint |
| 51239 | Rheumatoid arthritis of ankle |
| 53621 | Rheumatoid nodule |
| 56202 | [X]Seropositive rheumatoid arthritis, unspecified |
| 56838 | Caplan's syndrome |
| 59738 | Rheumatoid arthritis of elbow |
| 62401 | Polyneuropathy in rheumatoid arthritis |
| 63198 | Rheumatoid arthritis of DIP joint of finger |
| 63365 | Rheumatoid arthritis of distal radio-ulnar joint |
| 70221 | [X]Other specified rheumatoid arthritis |
| 70658 | Rheumatoid arthritis of talonavicular joint |
| 71784 | Rheumatoid arthritis of other tarsal joint |
| 73619 | Rheumatoid arthritis of subtalar joint |
| 93715 | [X]Other seropositive rheumatoid arthritis |
| 99414 | Rheumatoid arthritis of lesser MTP joint |
| 100187 | Disease activity score in rheumatoid arthritis |
| 100776 | Rheumatoid arthritis of sacro-iliac joint |
| 100914 | Rheumatoid arthritis of acromioclavicular joint |
| 102088 | Delivery of rehabilitation for rheumatoid arthritis |

Disease-modifying anti-rheumatic drugs (DMARDs) – product codes

| **Prodcode** | **Product name** |
| --- | --- |
| 265 | Chloroquine sulphate 68mg/5ml oral solution |
| 267 | Penicillamine 50mg tablets |
| 270 | Azathioprine 50mg powder for solution for injection vials |
| 283 | Myocrisin 10mg/0.5ml solution for injection ampoules (Sanofi) |
| 359 | Sulfasalazine 3g/100ml enema |
| 370 | Salazopyrin 3g/100ml Enema (Pharmacia Ltd) |
| 380 | Salazopyrin EN-Tabs 500mg (Pfizer Ltd) |
| 422 | Chloroquine phosphate 250mg tablets |
| 451 | Azathioprine 25mg tablets |
| 456 | Chloroquine sulphate 200mg tablets |
| 463 | Nivaquine 272.5mg(200mg base)/5ml Injection (Aventis Pharma) |
| 508 | Sulfasalazine 500mg gastro-resistant tablets |
| 516 | Chloroquine phosphate 250mg tablets and Proguanil 100mg tablets |
| 571 | Azathioprine 50mg tablets |
| 604 | Penicillamine 250mg tablets |
| 643 | Penicillamine 125mg tablets |
| 671 | Imuran 25mg Tablet (Wellcome Medical Division) |
| 672 | Hydroxychloroquine 200mg tablets |
| 770 | Azathioprine capsules |
| 823 | Methotrexate 2.5mg tablets |
| 877 | Methotrexate 10mg tablets |
| 972 | Neoral 25mg capsules (Novartis Pharmaceuticals UK Ltd) |
| 973 | Neoral 100mg capsules (Novartis Pharmaceuticals UK Ltd) |
| 1566 | Salazopyrin 500mg Tablet (Pharmacia Ltd) |
| 1626 | Ciclosporin 100mg/ml oral solution sugar free |
| 1899 | Imuran 50mg Tablet (Wellcome Medical Division) |
| 1905 | Neoral 100mg/ml oral solution (Novartis Pharmaceuticals UK Ltd) |
| 2837 | Ciclosporin 50mg capsules |
| 2838 | Ciclosporin 25mg capsules |
| 2920 | Sulfasalazine 500mg tablet |
| 3169 | Nivaquine 68mg/5ml Oral solution (Aventis Pharma) |
| 3224 | Nivaquine 200mg Tablet (Aventis Pharma) |
| 3267 | Myocrisin 50mg/0.5ml solution for injection ampoules (Sanofi) |
| 3325 | Avloclor 250mg tablets (AstraZeneca UK Ltd) |
| 3327 | Distamine 125mg tablets (Alliance Pharmaceuticals Ltd) |
| 3329 | Myocrisin 20mg/0.5ml solution for injection ampoules (Sanofi) |
| 3697 | Sulfasalazine 500mg suppositories |
| 3896 | Ciclosporin 100mg capsules |
| 3920 | Sandimmun 25mg capsules (Novartis Pharmaceuticals UK Ltd) |
| 3934 | Auranofin 3mg tablets |
| 4231 | Neoral 50mg capsules (Novartis Pharmaceuticals UK Ltd) |
| 4418 | Salazopyrin 500mg Suppository (Pharmacia Ltd) |
| 4470 | Sodium aurothiomalate 50mg/0.5ml solution for injection ampoules |
| 4946 | Plaquenil 200mg tablets (Sanofi) |
| 4970 | Leflunomide 100mg tablets |
| 4971 | Leflunomide 10mg tablets |
| 4978 | Salazopyrin 500mg tablets (Pfizer Ltd) |
| 5427 | Sulfasalazine 500mg tablets |
| 6230 | Proguanil 100mg & chloroquine 250mg tablets |
| 6882 | Adalimumab 40mg injection |
| 6934 | Leflunomide 20mg tablets |
| 7221 | Paludrine/Avloclor tablets anti-malarial travel pack (AstraZeneca UK Ltd) |
| 7336 | Methotrexate 12.5mg/0.5ml solution for injection pre-filled syringes |
| 7337 | Methotrexate 10mg/0.4ml solution for injection pre-filled syringes |
| 7497 | Sulfasalazine 250mg/5ml oral solution |
| 8327 | Methotrexate 50mg/3ml Injection |
| 8583 | Methotrexate 25mg/ml Injection |
| 8904 | Distamine 250mg tablets (Alliance Pharmaceuticals Ltd) |
| 9528 | Methotrexate 5mg/2ml solution for injection vials |
| 9845 | Oilatum scalp treatment shampoo (GlaxoSmithKline UK Ltd) |
| 10211 | Salazopyrin 500mg suppositories (Pfizer Ltd) |
| 10658 | Chloroquine phosphate 80mg/5ml oral solution |
| 10842 | Sodium aurothiomalate 10mg/0.5ml solution for injection ampoules |
| 11767 | Sulfasalazine 250mg/5ml oral suspension |
| 11959 | Distamine 50mg Tablet (Alliance Pharmaceuticals Ltd) |
| 12339 | Azamune 50mg Tablet (Penn Pharmaceuticals Ltd) |
| 12816 | Methotrexate 100mg/ml Injection |
| 13022 | Nivaquine 200mg tablets (Sanofi) |
| 13320 | Azathioprine 10mg tablets |
| 13321 | Salazopyrin 3g/100ml enema (Pfizer Ltd) |
| 13428 | Maxtrex 2.5mg tablets (Pfizer Ltd) |
| 13493 | Ridaura Tiltab 3mg tablets (Astellas Pharma Ltd) |
| 13494 | Sandimmun 100mg/ml oral solution (Novartis Pharmaceuticals UK Ltd) |
| 13556 | Sandimmun 100mg capsules (Novartis Pharmaceuticals UK Ltd) |
| 14054 | Salazopyrin 250mg/5ml oral suspension (Pfizer Ltd) |
| 14347 | Methotrexate 20mg/0.8ml solution for injection pre-filled syringes |
| 14348 | Metoject 20mg/2ml solution for injection pre-filled syringes (medac UK) |
| 14395 | Imuran 50mg powder for solution for injection vials (Aspen Pharma Trading Ltd) |
| 14748 | Methotrexate sodium 25mg/ml Injection |
| 14828 | Nivaquine 68mg/5ml syrup (Sanofi) |
| 14886 | Enbrel 25mg powder and solvent for solution for injection vials (Pfizer Ltd) |
| 15362 | Chloroquine sulphate 200mg/5ml solution for injection ampoules |
| 15373 | Sulfasalazine 500mg suppositories |
| 15596 | Sandimmun 50mg capsules (Novartis Pharmaceuticals UK Ltd) |
| 15921 | Etanercept 25mg powder and solvent for solution for injection vials |
| 16035 | Ciclosporin 10mg capsules |
| 16137 | Neoral 10mg capsules (Novartis Pharmaceuticals UK Ltd) |
| 16519 | Methotrexate 25mg/1ml solution for injection pre-filled syringes |
| 16522 | Arava 10mg tablets (Sanofi) |
| 16540 | Methotrexate 15mg/0.6ml solution for injection pre-filled syringes |
| 16570 | Methotrexate 7.5mg/0.3ml solution for injection pre-filled syringes |
| 16606 | Sodium aurothiomalate 20mg/0.5ml solution for injection ampoules |
| 16822 | Infliximab 100mg powder for solution for infusion vials |
| 17035 | Methotrexate 2.5mg/5ml oral suspension |
| 17642 | Arava 20mg tablets (Sanofi) |
| 17672 | Methotrexate 22.5mg/0.9ml solution for injection pre-filled syringes |
| 17880 | Salazopyrin 250mg/5ml Liquid (Pharmacia Ltd) |
| 18424 | Methotrexate sodium 2.5mg Tablet |
| 18460 | Arava 100mg tablets (Sanofi) |
| 18890 | Methotrexate 17.5mg/0.7ml solution for injection pre-filled syringes |
| 19072 | Oprisine 50mg Tablet (Opus Pharmaceuticals Ltd) |
| 19257 | Enbrel 50mg powder and solvent for solution for injection vials (Wyeth Pharmaceuticals) |
| 19370 | Ciclosporin 50mg/ml concentrate solution infusion |
| 20255 | Pendramine 250mg Tablet (Viatris Pharmaceuticals Ltd) |
| 20862 | Sulfasalazine 500mg gastro-resistant tablets (Actavis UK Ltd) |
| 20951 | Methotrexate 2.5mg tablets (Mercury Pharma Group Ltd) |
| 21753 | Maxtrex 10mg tablets (Pfizer Ltd) |
| 21889 | METHOTREXATE 25MG/1ML |
| 21899 | Immunoprin 50mg tablets (Ashbourne Pharmaceuticals Ltd) |
| 22392 | Remicade 100mg powder for solution for infusion vials (Merck Sharp & Dohme Ltd) |
| 22982 | Azathioprine 50mg/5ml oral solution |
| 23401 | Sulazine EC 500mg tablets (Genesis Pharmaceuticals Ltd) |
| 23441 | Malarivon 80mg/5ml syrup (Wallace Manufacturing Chemists Ltd) |
| 23850 | Humira 40mg Injection (Abbott Laboratories Ltd) |
| 24634 | Methotrexate 25mg/2.5ml solution for injection pre-filled syringes |
| 24783 | Methotrexate 50mg/2ml Injection |
| 26064 | Methotrexate 20mg/2ml solution for injection pre-filled syringes |
| 26261 | Berkaprine 50mg Tablet (Rorer Pharmaceuticals Ltd) |
| 26387 | Etanercept 50mg powder and solvent for solution for injection vials |
| 26790 | Sandimmun 50mg/ml Concentrate for solution for infusion (Novartis Pharmaceuticals UK Ltd) |
| 27342 | Maxtrex 2.5mg/ml Injection (Pharmacia Ltd) |
| 27400 | Metoject 15mg/1.5ml solution for injection pre-filled syringes (medac UK) |
| 27404 | Methotrexate 15mg/1.5ml solution for injection pre-filled syringes |
| 27579 | METHOTREXATE |
| 27642 | Methotrexate 27.5mg/1.1ml solution for injection pre-filled syringes |
| 28041 | Methotrexate 12.5mg/5ml oral suspension |
| 28490 | Rituximab 100mg/10ml solution for infusion vials |
| 29069 | Methotrexate 500mg/vial sterile powder |
| 29340 | Azathioprine 50mg tablets (IVAX Pharmaceuticals UK Ltd) |
| 29566 | Hydroxychloroquine 200mg/5ml oral solution |
| 29721 | Pendramine 125mg Tablet (Viatris Pharmaceuticals Ltd) |
| 30495 | Imuran 10mg Tablet (Wellcome Medical Division) |
| 30703 | Methotrexate 30mg/1.2ml solution for injection pre-filled syringes |
| 30780 | Methotrexate 2.5mg Tablet (Pharmacia Ltd) |
| 30925 | Penicillamine 250mg tablets (Actavis UK Ltd) |
| 30932 | Methotrexate 5mg/0.2ml solution for injection pre-filled syringes |
| 31120 | Penicillamine 125mg Tablet (IVAX Pharmaceuticals UK Ltd) |
| 31215 | Azathioprine 50mg tablets (Kent Pharmaceuticals Ltd) |
| 31216 | Penicillamine 125mg tablets (Actavis UK Ltd) |
| 31217 | Penicillamine 250mg tablets (A A H Pharmaceuticals Ltd) |
| 31667 | Sulfasalazine 500mg tablets (A A H Pharmaceuticals Ltd) |
| 31683 | Sulfasalazine 3g/100ml retention enema |
| 31949 | Sulfasalazine 500mg tablets (Actavis UK Ltd) |
| 32101 | Azathioprine 25mg tablets (A A H Pharmaceuticals Ltd) |
| 32111 | Methotrexate 2.5mg tablets (Hospira UK Ltd) |
| 32229 | Methotrexate 500mg/20ml solution for injection vials |
| 32418 | Kineret 100mg/0.67ml solution for injection pre-filled syringes (Swedish Orphan Biovitrum Ltd) |
| 32865 | Methotrexate 10mg/1ml solution for injection pre-filled syringes |
| 33601 | Metoject 25mg/2.5ml solution for injection pre-filled syringes (medac UK) |
| 33682 | Sulfasalazine 500mg Gastro-resistant tablet (DDSA Pharmaceuticals Ltd) |
| 33968 | Sulfasalazine 500mg Tablet (Approved Prescription Services Ltd) |
| 34258 | Methotrexate 20mg/0.8ml Injection (Central Homecare) |
| 34451 | Azathioprine 50mg tablets (Generics (UK) Ltd) |
| 34473 | Sulfasalazine 500mg tablets (Generics (UK) Ltd) |
| 34684 | Penicillamine 250mg tablets (Generics (UK) Ltd) |
| 34687 | Azathioprine 50mg tablets (A A H Pharmaceuticals Ltd) |
| 34816 | Azathioprine 25mg tablets (Generics (UK) Ltd) |
| 34894 | Sulfasalazine 500mg Gastro-resistant tablet (Ceretron Ltd) |
| 34929 | Methotrexate 10mg tablets (Hospira UK Ltd) |
| 35126 | Etanercept 50mg injection solution |
| 35402 | Methotrexate 7.5mg/0.75ml solution for injection pre-filled syringes |
| 35419 | Enbrel 25mg/0.5ml solution for injection pre-filled syringes (Pfizer Ltd) |
| 35518 | Azathioprine 50mg/5ml oral suspension |
| 35752 | Methotrexate 7.5mg/5ml oral suspension |
| 35865 | Metoject 7.5mg/0.75ml solution for injection pre-filled syringes (medac UK) |
| 36008 | Etanercept 25mg/0.5ml solution for injection pre-filled syringes |
| 36167 | Methotrexate 1g/10ml solution for injection vials |
| 36294 | Rituximab 500mg/50ml solution for infusion vials |
| 36556 | Enbrel 50mg Solution for injection (Pfizer Consumer Healthcare Ltd) |
| 36726 | Anakinra 100mg/0.67ml solution for injection pre-filled syringes |
| 36792 | Azathioprine 250mg/5ml oral solution |
| 36800 | Methotrexate 10mg/5ml oral solution |
| 36849 | Methotrexate 10mg/5ml oral suspension |
| 37117 | Metoject 10mg/1ml solution for injection pre-filled syringes (medac UK) |
| 38056 | Ciclosporin 50mg/1ml solution for infusion ampoules |
| 39111 | Rituximab 10mg/ml concentrated intravenous infusion |
| 39115 | Azathioprine 10mg capsules |
| 40170 | Penicillamine 125mg tablets (Generics (UK) Ltd) |
| 40273 | Methotrexate 20mg/0.4ml solution for injection pre-filled syringes |
| 40280 | Metoject 7.5mg/0.15ml solution for injection pre-filled syringes (medac UK) |
| 40281 | Methotrexate 15mg/0.3ml solution for injection pre-filled syringes |
| 40284 | Metoject 15mg/0.3ml solution for injection pre-filled syringes (medac UK) |
| 40292 | Metoject 20mg/0.4ml solution for injection pre-filled syringes (medac UK) |
| 40293 | Metoject 25mg/0.5ml solution for injection pre-filled syringes (medac UK) |
| 40301 | Methotrexate 7.5mg/0.15ml solution for injection pre-filled syringes |
| 40328 | Methotrexate 25mg/0.5ml solution for injection pre-filled syringes |
| 40356 | Metoject 10mg/0.2ml solution for injection pre-filled syringes (medac UK) |
| 40371 | Methotrexate 10mg/0.2ml solution for injection pre-filled syringes |
| 41058 | Enbrel Paediatric 25mg powder and solvent for solution for injection vials (Pfizer Ltd) |
| 41086 | Methotrexate 5g/50ml solution for infusion vials |
| 41104 | Methotrexate 2.5mg tablets (Wockhardt UK Ltd) |
| 41502 | Tocilizumab 80mg/4ml solution for infusion vials |
| 41585 | Methotrexate sodium 2.5mg Tablet (Wyeth Pharmaceuticals) |
| 41620 | Azathioprine 50mg tablets (Teva UK Ltd) |
| 41670 | Azathioprine 50mg Tablet (C P Pharmaceuticals Ltd) |
| 42178 | Sulfasalazine 500mg Tablet (Berk Pharmaceuticals Ltd) |
| 42448 | Deximune 50mg capsules (Dexcel-Pharma Ltd) |
| 42449 | Deximune 100mg capsules (Dexcel-Pharma Ltd) |
| 42637 | Deximune 25mg capsules (Dexcel-Pharma Ltd) |
| 42924 | Ciclosporin 250mg/5ml solution for infusion ampoules |
| 42988 | Imuran 50mg tablets (Aspen Pharma Trading Ltd) |
| 43077 | Imuran 25mg tablets (Aspen Pharma Trading Ltd) |
| 43562 | Azathioprine 50mg tablets (Actavis UK Ltd) |
| 43703 | Cimzia 200mg/1ml solution for injection pre-filled syringes (UCB Pharma Ltd) |
| 44100 | Certolizumab pegol 200mg/1ml solution for injection pre-filled syringes |
| 44183 | Sulfasalazine 500mg gastro-resistant tablets (A A H Pharmaceuticals Ltd) |
| 44411 | Chloroquine sulphate 200mg/5ml solution for injection ampoules (Sanofi) |
| 44908 | Methotrexate 30mg/0.6ml solution for injection pre-filled syringes |
| 45023 | Quinoric 200mg tablets (Bristol Laboratories Ltd) |
| 45165 | Methotrexate 20mg/1ml solution for injection pre-filled syringes |
| 45558 | Methotrexate 25mg/1.25ml solution for injection pre-filled syringes |
| 46039 | Methotrexate 30mg/1.5ml solution for injection pre-filled syringes |
| 46098 | Metoject 12.5mg/0.25ml solution for injection pre-filled syringes (medac UK) |
| 46129 | Methotrexate 22.5mg/0.45ml solution for injection pre-filled syringes |
| 46152 | Methotrexate 12.5mg/0.25ml solution for injection pre-filled syringes |
| 46156 | Methotrexate 17.5mg/0.35ml solution for injection pre-filled syringes |
| 46197 | Metoject 22.5mg/0.45ml solution for injection pre-filled syringes (medac UK) |
| 46265 | Metoject 17.5mg/0.35ml solution for injection pre-filled syringes (medac UK) |
| 46348 | Tocilizumab 200mg/10ml solution for infusion vials |

Smoking status - medcodes

| **medcode** | **Description** | **Ever or never smoker** |
| --- | --- | --- |
| 33 | Never smoked tobacco | Never |
| 60 | Current non-smoker | Never |
| 90 | Ex smoker | Ever |
| 93 | Cigarette smoker | Ever |
| 776 | Stopped smoking | Ever |
| 1822 | Very heavy smoker - 40+cigs/d | Ever |
| 1823 | Smoker | Ever |
| 1878 | Moderate smoker - 10-19 cigs/d | Ever |
| 3568 | Heavy smoker - 20-39 cigs/day | Ever |
| 7622 | Smoking cessation advice | Ever |
| 9045 | Advice on smoking | Ever |
| 10184 | Pregnancy smoking advice | Ever |
| 10211 | Smoking cessation milestones | Ever |
| 10558 | Current smoker | Ever |
| 10742 | Referral to stop-smoking clinic | Ever |
| 11356 | Seen by smoking cessation advisor | Ever |
| 11527 | DNA - Did not attend smoking cessation clinic | Ever |
| 11788 | Non-smoker | Never |
| 12240 | Trying to give up smoking | Ever |
| 12878 | Date ceased smoking | Ever |
| 12941 | Occasional smoker | Ever |
| 12942 | Smoker - amount smoked | Ever |
| 12943 | Cigar smoker | Ever |
| 12944 | Light smoker - 1-9 cigs/day | Ever |
| 12946 | Ex-smoker - amount unknown | Ever |
| 12947 | Pipe smoker | Ever |
| 12951 | Smoking restarted | Ever |
| 12952 | Smoking started | Ever |
| 12953 | Attends stop smoking monitor. | Ever |
| 12955 | Ex-moderate smoker (10-19/day) | Ever |
| 12956 | Ex-heavy smoker (20-39/day) | Ever |
| 12957 | Ex-light smoker (1-9/day) | Ever |
| 12958 | Trivial smoker - < 1 cig/day | Ever |
| 12959 | Ex-very heavy smoker (40+/day) | Ever |
| 12961 | Ex-trivial smoker (<1/day) | Ever |
| 12964 | Keeps trying to stop smoking | Ever |
| 12966 | Smoking reduced | Ever |
| 16717 | Smokers' cough | Ever |
| 18573 | Referral to smoking cessation advisor | Ever |
| 19488 | Ex cigar smoker | Ever |
| 26096 | Smokes drugs | Ever |
| 26470 | Ex pipe smoker | Ever |
| 30423 | Thinking about stopping smoking | Ever |
| 30762 | Not interested in stopping smoking | Ever |
| 31114 | Ready to stop smoking | Ever |
| 34126 | Negotiated date for cessation of smoking | Ever |
| 38112 | Smoking cessation programme start date | Ever |
| 40418 | Refuses stop smoking monitor | Ever |
| 41042 | Smoking cessation advice provided by community pharmacist | Ever |
| 41979 | Smoking restarted | Ever |
| 74907 | Smoking cessation therapy | Ever |
| 90522 | Smoking cessation therapy NOS | Ever |
| 91708 | Other specified smoking cessation therapy | Ever |
| 94958 | Smoking cessation drug therapy | Ever |
| 97210 | Ex-cigarette smoker | Ever |
| 98137 | Brief intervention for smoking cessation | Ever |
| 98154 | Referral to NHS stop smoking service | Ever |
| 98245 | Stop smoking face to face follow-up | Ever |
| 99838 | Recently stopped smoking | Ever |
| 100099 | Smoking cessation advice declined | Ever |
| 100495 | Ex roll-up cigarette smoker | Ever |
| 101338 | Failed attempt to stop smoking | Ever |
| 101764 | Practice based smoking cessation programme start date | Ever |
| 102361 | Referral for smoking cessation service offered | Ever |
| 102951 | Lost to smoking cessation follow-up | Ever |
| 103507 | Stop smoking service opportunity signposted | Ever |
| 104185 | Smoking cessation drug therapy declined | Ever |
| 104230 | Smoking cessation programme declined | Ever |
| 105999 | Smokes drugs in cigarette form | Ever |

Smoking status – Product codes

| **prodcode** | **Product name** |
| --- | --- |
| 42221 | Nicotine 4mg lozenges sugar free (Teva UK Ltd) |
| 38958 | Nicotinell 1mg lozenges (Novartis Consumer Health UK Ltd) |
| 41765 | Nicotinell Mint 2mg medicated chewing gum (Novartis Consumer Health UK Ltd) |
| 5502 | Nicotine 15mg/16hours transdermal patches |
| 39123 | Nicotine 25mg/16hours transdermal patches |
| 58034 | Nicotine 2.5mg orodispersible films sugar free |
| 5784 | Nicotine 4mg lozenges sugar free |
| 42047 | Nicotinell Liquorice 4mg medicated chewing gum (Novartis Consumer Health UK Ltd) |
| 5457 | Nicotine 5mg/16hours transdermal patches |
| 41860 | Nicotine bitartrate 2mg Sublingual tablet |
| 27414 | Varenicline 1mg tablets |
| 41923 | Nicotine 15mg/16 hours transdermal patches and Nicotine 2mg medicated chewing gum sugar free |
| 5515 | Nicotine 1mg Lozenge |
| 46717 | Nicotine 15mg inhalation cartridges with device |
| 14556 | TRANSDERMAL NICOTINE PATCH 10 MG |
| 5606 | Nicotinell tts 20 sq cm Transdermal patch (Novartis Consumer Health UK Ltd) |
| 40617 | Nicotinell TTS 20 patches (Novartis Consumer Health UK Ltd) |
| 3818 | Nicotinell tts 30 sq cm Transdermal patch (Novartis Consumer Health UK Ltd) |
| 25510 | Nicotine 2mg mint flavour chewing-gum |
| 37646 | Nicotine 1.5mg lozenges sugar free |
| 41879 | Nicotinell Liquorice 2mg medicated chewing gum (Novartis Consumer Health UK Ltd) |
| 5946 | Nicotinell 2mg Medicated chewing-gum (Novartis Consumer Health UK Ltd) |
| 45504 | Nicotine 1mg/dose oromucosal spray sugar free |
| 6448 | Nicotine 21mg/24hours transdermal patches |
| 8297 | NICOTINE TRANSDERMAL PATCH 20CM |
| 42048 | Nicotine bitartrate 1mg lozenges sugar free |
| 33392 | Nicotine 22mg/24 hr Transdermal patch |
| 41909 | Nicotinell Mint 4mg medicated chewing gum (Novartis Consumer Health UK Ltd) |
| 8534 | TRANSDERMAL NICOTINE PATCH 5 MG |
| 5944 | Nicotine 10mg inhalation cartridges with device |
| 6698 | Nicotinell 2mg lozenges (Novartis Consumer Health UK Ltd) |
| 41881 | Nicotinell Classic 2mg medicated chewing gum (Novartis Consumer Health UK Ltd) |
| 9591 | Nicotine 14mg/24hours transdermal patches |
| 5758 | Nicotine 4mg medicated chewing gum sugar free |
| 116 | NICOTINE TRANSDERMAL PATCH 30CM |
| 8571 | Nicotine 500micrograms/dose nasal spray |
| 27412 | Varenicline 1mg tablets and Varenicline 500microgram tablets |
| 11718 | Nicotine 2mg sublingual tablets sugar free |
| 13048 | Nicotinell 4mg Medicated chewing-gum (Novartis Consumer Health UK Ltd) |
| 5531 | Nicotinell 1mg Lozenge (Novartis Consumer Health UK Ltd) |
| 41808 | Nicotinell Fruit 4mg medicated chewing gum (Novartis Consumer Health UK Ltd) |
| 5115 | Bupropion 150mg modified-release tablets |
| 7303 | Nicotinell tts 10 sq cm Transdermal patch (Novartis Consumer Health UK Ltd) |
| 25516 | Nicotine 4mg mint flavour chewing-gum |
| 42011 | Nicotinell Classic 4mg medicated chewing gum (Novartis Consumer Health UK Ltd) |
| 46588 | Nicotinell Icemint 2mg medicated chewing gum (Novartis Consumer Health UK Ltd) |
| 40683 | Nicotinell TTS 10 patches (Novartis Consumer Health UK Ltd) |
| 46701 | Nicotinell Icemint 4mg medicated chewing gum (Novartis Consumer Health UK Ltd) |
| 41931 | Nicotinell Fruit 2mg medicated chewing gum (Novartis Consumer Health UK Ltd) |
| 9806 | Nicotine 2mg lozenges sugar free |
| 35089 | Varenicline 500microgram tablets |
| 9804 | Nicotine 7mg/24hours transdermal patches |
| 42286 | Nicotine bitartrate 2mg lozenges sugar free |
| 6323 | Nicotine 2mg medicated chewing gum sugar free |
| 40620 | Nicotinell TTS 30 patches (Novartis Consumer Health UK Ltd) |
| 55590 | Nicotine 11mg/24 hr Transdermal patch |
| 5479 | Nicotine 10mg/16hours transdermal patches |

Diabetes mellitus (type 2) - medcodes

| medcode | readterm |
| --- | --- |
| 506 | Non-insulin dependent diabetes mellitus |
| 608 | Follow-up diabetic assessment |
| 711 | Diabetes mellitus |
| 758 | Type 2 diabetes mellitus |
| 1038 | Insulin dependent diabetes mellitus |
| 1173 | Pure hyperglyceridaemia |
| 1323 | Diabetic retinopathy |
| 1407 | Insulin treated Type 2 diabetes mellitus |
| 1647 | Insulin dependent diabetes mellitus |
| 1682 | Diabetes mellitus with ketoacidosis |
| 1684 | Diabetic on oral treatment |
| 1789 | [D]Hyperglycaemia |
| 2340 | Diabetic amyotrophy |
| 2342 | Diabetic neuropathy |
| 2378 | Diabetic - poor control |
| 2379 | Seen in diabetic clinic |
| 2471 | Nephrotic syndrome in diabetes mellitus |
| 2475 | Diabetic nephropathy |
| 2478 | Brittle diabetes |
| 2664 | Gestational diabetes mellitus |
| 2986 | Preproliferative diabetic retinopathy |
| 3286 | Proliferative diabetic retinopathy |
| 3295 | [D]Impaired glucose tolerance test |
| 3550 | Diabetic monitoring |
| 3837 | Diabetic maculopathy |
| 4513 | Non-insulin dependent diabetes mellitus |
| 5002 | Diabetic polyneuropathy |
| 5884 | NIDDM - Non-insulin dependent diabetes mellitus |
| 6125 | Diabetic annual review |
| 6430 | Attending diabetes clinic |
| 6509 | Insulin dependent diabetes mellitus with retinopathy |
| 6791 | Insulin dependent diabetes mellitus - poor control |
| 6813 | H/O: diabetes mellitus |
| 7045 | H/O: Admission in last year for diabetes foot problem |
| 7059 | Admit diabetic emergency |
| 7069 | Background diabetic retinopathy |
| 7328 | Cellulitis in diabetic foot |
| 7563 | Diabetic on diet only |
| 7777 | Referral to diabetologist |
| 7795 | Diabetes mellitus with neuropathy |
| 8306 | Referral to diabetes nurse |
| 8403 | Non-insulin dependent diabetes mellitus - poor control |
| 8414 | Pt advised re diabetic diet |
| 8446 | Gestational diabetes mellitus |
| 8618 | Seen by diabetic liaison nurse |
| 8836 | Diabetes management plan given |
| 8842 | Diabetic on insulin |
| 9013 | Unstable diabetes |
| 9145 | DNA - Did not attend diabetic clinic |
| 9835 | O/E - diabetic maculopathy present both eyes |
| 9881 | Mixed diabetic ulcer - foot |
| 9897 | Diabetes monitoring admin. |
| 9958 | Hb. A1C - diabetic control |
| 9974 | Seen in diabetic eye clinic |
| 10042 | [D]Impaired glucose tolerance |
| 10098 | Other specified diabetes mellitus with other spec comps |
| 10099 | Advanced diabetic maculopathy |
| 10278 | Diabetes mellitus arising in pregnancy |
| 10642 | Dietary advice for diabetes mellitus |
| 10659 | Diabetic cataract |
| 10755 | Non proliferative diabetic retinopathy |
| 10791 | [D]Impaired fasting glycaemia |
| 10824 | Seen in diabetic foot clinic |
| 10977 | Diabetic peripheral neuropathy screening |
| 10983 | Impaired fasting glycaemia |
| 11018 | Diabetic retinopathy 12 month review |
| 11041 | Excepted from diabetes qual indicators: Patient unsuitable |
| 11094 | Under care of diabetic foot screener |
| 11129 | O/E - left eye background diabetic retinopathy |
| 11348 | Excepted from diabetes quality indicators: Informed dissent |
| 11359 | Diabetes mellitus during pregnancy/childbirth/puerperium |
| 11433 | O/E - right eye background diabetic retinopathy |
| 11471 | Diabetes medication review |
| 11551 | Diabetes mellitus induced by steroids |
| 11599 | Pan retinal photocoagulation for diabetes |
| 11626 | Diabetic retinopathy NOS |
| 11663 | Neuropathic diabetic ulcer - foot |
| 11677 | Refer to diabetic foot screener |
| 11818 | [D]Glucose tolerance test abnormal |
| 11848 | Renal diabetes |
| 11930 | Under care of diabetes specialist nurse |
| 11977 | Referral to diabetes nurse |
| 12030 | Diabetes monitoring 3rd letter |
| 12213 | Patient on maximal tolerated therapy for diabetes |
| 12225 | Refer, diabetic liaison nurse |
| 12247 | Diabetic foot examination not indicated |
| 12262 | Diabetic retinopathy screening refused |
| 12307 | Diabetes care by hospital only |
| 12506 | Diabetes: practice programme |
| 12507 | Seen by diabetic liaison nurse |
| 12640 | Type 2 diabetes mellitus with nephropathy |
| 12675 | Diabetes: shared care programme |
| 12682 | Patient offered diabetes structured education programme |
| 12703 | Education score - diabetes |
| 12736 | Type 2 diabetes mellitus with gangrene |
| 13057 | Health education - diabetes |
| 13067 | Diabetic monitoring NOS |
| 13069 | Has seen dietician - diabetes |
| 13070 | Initial diabetic assessment |
| 13071 | Diabetic - good control |
| 13074 | Diabetic diet |
| 13078 | Diabetic weight reducing diet |
| 13097 | O/E - right eye proliferative diabetic retinopathy |
| 13099 | O/E - right eye preproliferative diabetic retinopathy |
| 13101 | O/E - left eye proliferative diabetic retinopathy |
| 13102 | O/E - right eye diabetic maculopathy |
| 13103 | O/E - left eye preproliferative diabetic retinopathy |
| 13108 | O/E - left eye diabetic maculopathy |
| 13191 | Diabetes clinic administration |
| 13192 | Diabetes monitor. check done |
| 13194 | Diabetes monitoring 1st letter |
| 13195 | Diabetes monitoring 2nd letter |
| 13196 | Fundoscopy - diabetic check |
| 13197 | Attends diabetes monitoring |
| 13279 | Other specified diabetes mellitus with renal complications |
| 13597 | Hb. A1C < 7% - good control |
| 13604 | Hb. A1C > 10% - bad control |
| 13678 | Referral to diabetic liaison nurse |
| 14049 | Hb. A1C - diabetic control NOS |
| 14050 | HbA1 - diabetic control |
| 14803 | Diabetes mellitus, adult onset, no mention of complication |
| 14889 | Maturity onset diabetes |
| 14980 | Renal glycosuria |
| 15690 | Diabetes mellitus with ketoacidotic coma |
| 16230 | Diabetes mellitus with neurological manifestation |
| 16490 | Diabetic treatment changed |
| 16491 | Diabetes mellitus with polyneuropathy |
| 16502 | Diabetes mellitus with renal manifestation |
| 16881 | [V]Dietary counselling in diabetes mellitus |
| 17067 | Autonomic neuropathy due to diabetes |
| 17095 | O/E - Right diabetic foot at risk |
| 17236 | H/O: insulin therapy |
| 17247 | Diabetic mononeuritis NOS |
| 17262 | Non-insulin-dependent diabetes mellitus with retinopathy |
| 17313 | Diabetic iritis |
| 17859 | Type 2 diabetes mellitus |
| 17869 | Diabetic-uncooperative patient |
| 17886 | Diabetic - follow-up default |
| 18056 | Foot abnormality - diabetes related |
| 18142 | Diabetic cheiroarthropathy |
| 18143 | Type II diabetes mellitus with arthropathy |
| 18167 | Annual diabetic blood test |
| 18209 | Type 2 diabetes mellitus with renal complications |
| 18219 | Type II diabetes mellitus |
| 18264 | Insulin treated Type II diabetes mellitus |
| 18278 | Insulin treated Type 2 diabetes mellitus |
| 18311 | Diabetic retinopathy screening |
| 18390 | Type 2 diabetes mellitus with persistent microalbuminuria |
| 18396 | Adverse reaction to metformin hydrochloride |
| 18425 | Type 2 diabetes mellitus with polyneuropathy |
| 18496 | Type 2 diabetes mellitus with retinopathy |
| 18505 | IDDM-Insulin dependent diabetes mellitus |
| 18662 | Diabetic retinopathy 6 month review |
| 18747 | Diabetic retinopathy screening not indicated |
| 18777 | Type 2 diabetes mellitus with renal complications |
| 18824 | Diabetic foot examination declined |
| 19381 | Referral to diabetic eye clinic |
| 19739 | Diabetic retinopathy screening offered |
| 20696 | Injection sites - diabetic |
| 20900 | Diabetes monitored |
| 21472 | Neonatal diabetes mellitus |
| 21482 | Diabetes mellitus with hyperosmolar coma |
| 21689 | Diabetic lipid lowering diet |
| 22023 | Diabetic - poor control NOS |
| 22130 | Diabetes monitoring default |
| 22305 | Adverse reaction to gliclazide |
| 22487 | Secondary diabetes mellitus |
| 22573 | Diabetes mellitus NOS with neurological manifestation |
| 22823 | Diabetic foot examination |
| 22884 | Type II diabetes mellitus |
| 22959 | Chronic hyperglycaemia |
| 22967 | Retinal abnormality - diabetes related |
| 23340 | Insulin poisoning |
| 24327 | Ischaemic ulcer diabetic foot |
| 24363 | Diabetic stabilisation |
| 24458 | Type II diabetes mellitus - poor control |
| 24490 | Diabetes mellitus, juvenile type, no mention of complication |
| 24571 | Asymptomatic diabetic neuropathy |
| 24693 | Non-insulin dependent diabetes mellitus with arthropathy |
| 24694 | Insulin dependent diabetes mellitus with mononeuropathy |
| 24836 | Type 2 diabetes mellitus with nephropathy |
| 25041 | Dietary advice for type II diabetes |
| 25446 | [D]Elevated blood glucose level |
| 25591 | Type 2 diabetes mellitus with exudative maculopathy |
| 25627 | Type 2 diabetes mellitus - poor control |
| 25636 | Diabetic diet - poor compliance |
| 26054 | Type 2 diabetes mellitus with persistent proteinuria |
| 26108 | Steroid induced diabetes mellitus without complication |
| 26603 | Refuses diabetes monitoring |
| 26604 | Diabetic diet - good compliance |
| 26605 | Attended diabetes structured education programme |
| 26664 | O/E - Left diabetic foot at risk |
| 26666 | O/E - Right diabetic foot at low risk |
| 26667 | O/E - Left diabetic foot at low risk |
| 26855 | Unstable insulin dependent diabetes mellitus |
| 27891 | Diabetic Charcot arthropathy |
| 27921 | Foot abnormality - diabetes related |
| 28574 | Exception reporting: diabetes quality indicators |
| 28769 | Diabetic on insulin and oral treatment |
| 28856 | Transition of diabetes care options discussed |
| 28873 | Diabetic 6 month review |
| 29041 | Date diabetic treatment start |
| 29218 | Hb. A1C 7-10% - borderline |
| 29979 | Non-insulin-dependent diabetes mellitus without complication |
| 30127 | H/O: Admission in last year for hyperglycaemic disorder |
| 30205 | [D]Drug induced hyperglycaemia |
| 30247 | Adverse reaction to insulins |
| 30477 | High risk proliferative diabetic retinopathy |
| 30648 | Did not attend diabetic retinopathy clinic |
| 31053 | [D]Widespread diabetic foot gangrene |
| 31141 | Diabetes monitor.phone invite |
| 31156 | O/E - Left diabetic foot at moderate risk |
| 31157 | O/E - Right diabetic foot at moderate risk |
| 31161 | [D]Impaired fasting glucose |
| 31171 | O/E - Right diabetic foot at high risk |
| 31172 | O/E - Left diabetic foot at high risk |
| 31240 | Diabetes monitor.verbal invite |
| 31241 | Diabetes monitoring admin.NOS |
| 31310 | Insulin dependent diabetes maturity onset |
| 31790 | Polyneuropathy in diabetes |
| 32193 | Steroid induced diabetes |
| 32359 | Perceived control of insulin-dependent diabetes |
| 32403 | Diabetes mellitus with gangrene |
| 32556 | Diabetes with gangrene |
| 32619 | Patient diabetes education review |
| 32627 | Type 2 diabetes mellitus with ketoacidosis |
| 32739 | Seen in community diabetes specialist clinic |
| 32770 | Glucose tol. test diabetic |
| 33254 | Diabetes mellitus with ophthalmic manifestation |
| 33343 | Diabetes mellitus with other specified manifestation |
| 33807 | Diabetes mellitus, adult with gangrene |
| 33969 | Malnutrition-related diabetes mellitus with ketoacidosis |
| 34152 | Diabetic peripheral angiopathy |
| 34268 | Type 2 diabetes mellitus with neurological complications |
| 34283 | Diabetes mellitus NOS with ophthalmic manifestation |
| 34450 | Hyperosmolar non-ketotic state in type 2 diabetes mellitus |
| 34528 | Diabetes well being questionnaire |
| 34541 | Private referral to diabetologist |
| 34639 | Diabetes mellitus during pregnancy - baby delivered |
| 34912 | Non-insulin dependent diabetes mellitus with ulcer |
| 35105 | Diabetes mellitus, adult onset, with renal manifestation |
| 35107 | Diabetes mellitus with nephropathy NOS |
| 35116 | O/E - Left diabetic foot - ulcerated |
| 35316 | O/E - Right diabetic foot - ulcerated |
| 35321 | Non-urgent diabetic admission |
| 35383 | Diabetic patient unsuitable for digital retinal photography |
| 35385 | Type 2 diabetes mellitus with neuropathic arthropathy |
| 35397 | Insulin resistance |
| 35399 | Diabetes mellitus with peripheral circulatory disorder |
| 35785 | Chronic painful diabetic neuropathy |
| 36633 | Hyperosmolar non-ketotic state in type 2 diabetes mellitus |
| 36669 | Diabetic monitoring not required |
| 36695 | Diabetes mellitus autosomal dominant type 2 |
| 37315 | Diabetic mononeuropathy |
| 37648 | Insulin treated non-insulin dependent diabetes mellitus |
| 37806 | Type 2 diabetes mellitus with peripheral angiopathy |
| 38078 | Understands diet - diabetes |
| 38103 | Seen in diabetic nurse consultant clinic |
| 38129 | Seen in community diabetic specialist nurse clinic |
| 38130 | Diabetes wellbeing questionnaire |
| 38617 | Other specified diabetes mellitus with ketoacidosis |
| 38986 | Diabetes mellitus with no mention of complication |
| 39317 | Diabetes mellitus, adult onset, + neurological manifestation |
| 39406 | Reaven's syndrome |
| 39420 | Myasthenic syndrome due to diabetic amyotrophy |
| 39481 | Metabolic syndrome X |
| 39809 | Insulin dependent diab mell with neuropathic arthropathy |
| 40023 | Diabetes mellitus, juvenile type, with hyperosmolar coma |
| 40401 | Non-insulin dependent diabetes mellitus with gangrene |
| 40463 | HbA1 7 - 10% - borderline control |
| 40962 | Non-insulin dependent d m with neuropathic arthropathy |
| 41389 | Diabetes mellitus, adult onset, + ophthalmic manifestation |
| 41686 | [X]Other specified diabetes mellitus |
| 41716 | Insulin dependent diabetes mellitus with polyneuropathy |
| 42360 | HbA1 < 7% - good control |
| 42505 | Diabetes mellitus NOS with ketoacidosis |
| 42567 | Diabetes mellitus, juvenile type, with ketoacidotic coma |
| 42762 | Type 2 diabetes mellitus with retinopathy |
| 43139 | Diabetes mellitus, adult onset, with hyperosmolar coma |
| 43227 | Type II diabetes mellitus with multiple complications |
| 43453 | Diabetes mellitus autosomal dominant |
| 43785 | Non-insulin dependent diabetes mellitus with hypoglyca coma |
| 43857 | Lipoatrophic diabetes mellitus |
| 43951 | Diabetic - cooperative patient |
| 44033 | Diabetic mononeuritis multiplex |
| 44260 | Insulin dependent diabetes mellitus with diabetic cataract |
| 44312 | Informed dissent for diabetes national audit |
| 44440 | Insulin dependent diabetes mellitus with hypoglycaemic coma |
| 44443 | Insulin dependent diabetes mellitus with ulcer |
| 44779 | Type 2 diabetes mellitus with diabetic cataract |
| 44982 | Type 2 diabetes mellitus with diabetic cataract |
| 45250 | Under care of diabetic liaison nurse |
| 45276 | Insulin dependent diabetes mellitus with multiple complicat |
| 45467 | Non-insulin dependent diabetes mellitus with polyneuropathy |
| 45491 | Diabetes mellitus with unspecified complication |
| 45913 | Type 2 diabetes mellitus - poor control |
| 45919 | Type 2 diabetes mellitus with neurological complications |
| 46079 | HbA1 > 10% - bad control |
| 46150 | Type 2 diabetes mellitus with gangrene |
| 46290 | Other specified diabetes mellitus with multiple comps |
| 46521 | Seen by diabetologist |
| 46577 | Diabetes: shared care in pregnancy - diabetol and obstet |
| 46624 | Maturity onset diabetes in youth |
| 46917 | Type 2 diabetes mellitus with hypoglycaemic coma |
| 46963 | Insulin-dependent diabetes mellitus with renal complications |
| 47011 | Referral to diabetes structured education programme |
| 47032 | Diabetes care plan agreed |
| 47058 | Discharged from care of diabetes specialist nurse |
| 47144 | O/E - diabetic maculopathy absent both eyes |
| 47315 | Type II diabetes mellitus - poor control |
| 47321 | Type 2 diabetes mellitus with ophthalmic complications |
| 47328 | O/E - right eye stable treated prolif diabetic retinopathy |
| 47341 | Diabetic crisis monitoring |
| 47370 | Diabetology D.V. done |
| 47377 | Other specified diabetes mellitus with ophthalmic complicatn |
| 47409 | Type II diabetes mellitus with polyneuropathy |
| 47584 | Advanced diabetic retinal disease |
| 47816 | Type II diabetes mellitus with neuropathic arthropathy |
| 47954 | Type 2 diabetes mellitus without complication |
| 48078 | Acute painful diabetic neuropathy |
| 48192 | Type II diabetes mellitus with diabetic cataract |
| 49074 | Type 2 diabetes mellitus with ulcer |
| 49276 | Insulin-dependent diabetes mellitus with ophthalmic comps |
| 49559 | Diabetes mellitus during pregnancy - baby not yet delivered |
| 49640 | O/E - left chronic diabetic foot ulcer |
| 49655 | Type II diabetes mellitus with retinopathy |
| 49686 | Adverse reaction to tolbutamide |
| 49869 | Type 2 diabetes mellitus with arthropathy |
| 49884 | Diabetic pre-pregnancy counselling |
| 50175 | Diabetic foot risk assessment |
| 50225 | Type II diabetes mellitus with renal complications |
| 50429 | Non-insulin-dependent diabetes mellitus with ophthalm comps |
| 50527 | Type II diabetes mellitus with polyneuropathy |
| 50609 | Pre-existing diabetes mellitus, non-insulin-dependent |
| 50813 | Type II diabetes mellitus with mononeuropathy |
| 50937 | Referral to diabetes preconception counselling clinic |
| 50960 | Pre-existing diabetes mellitus, insulin-dependent |
| 50972 | Diabetes mellitus NOS with no mention of complication |
| 51261 | Insulin dependent diabetes mellitus |
| 51697 | Secondary pancreatic diabetes mellitus |
| 51756 | Type 2 diabetes mellitus with ketoacidotic coma |
| 52041 | O/E - left eye stable treated prolif diabetic retinopathy |
| 52104 | Insulin dependent diabetes mellitus with multiple complicatn |
| 52212 | [X]Diabetes mellitus |
| 52236 | Malnutrition-related diabetes mellitus |
| 52237 | Patient held diabetic record issued |
| 52283 | Insulin-dependent diabetes mellitus with neurological comps |
| 52303 | Non-insulin-dependent diabetes mellitus with renal comps |
| 52630 | O/E - sight threatening diabetic retinopathy |
| 53200 | Diabetes mellitus, juvenile type, with ketoacidosis |
| 53238 | Diabetic drug side effects |
| 53392 | Type II diabetes mellitus without complication |
| 53634 | [D]Gangrene of toe in diabetic |
| 54212 | Non-insulin-dependent d m with peripheral angiopath |
| 54419 | Diabetes key contact |
| 54600 | Unstable insulin dependent diabetes mellitus |
| 54601 | Under care of diabetologist |
| 54773 | Reaven's syndrome |
| 54856 | Diabetes mellitus, adult onset, with ketoacidosis |
| 54899 | Type II diabetes mellitus with peripheral angiopathy |
| 55075 | Type II diabetes mellitus with ulcer |
| 55123 | Date diabetic treatment stopp. |
| 55431 | Pre-existing diabetes mellitus, unspecified |
| 55842 | Non-insulin-dependent diabetes mellitus with neuro comps |
| 56268 | Type II diabetes mellitus with hypoglycaemic coma |
| 56448 | Insulin-dependent diabetes without complication |
| 56803 | NIDDM with peripheral circulatory disorder |
| 57278 | Type II diabetes mellitus with renal complications |
| 57333 | Diabetic cheiropathy |
| 57389 | Patient consent given for addition to diabetic register |
| 57621 | Insulin dependent diabetes mellitus with nephropathy |
| 57723 | Referral to diabetic register |
| 58133 | Discharge by diabetic liaison nurse |
| 58159 | Insulin therapy declined |
| 58604 | Type II diabetes mellitus with retinopathy |
| 58639 | Patient held diabetic record declined |
| 59253 | Type 2 diabetes mellitus with arthropathy |
| 59288 | Other specified diabetes mellitus with coma |
| 59365 | Non-insulin dependent diabetes mellitus with nephropathy |
| 59725 | Type II diabetes mellitus with ophthalmic complications |
| 59903 | Diabetic amyotrophy |
| 59991 | Maturity onset diabetes in youth type 2 |
| 60499 | Insulin dependent diabetes mellitus with gangrene |
| 60699 | Type 2 diabetes mellitus with peripheral angiopathy |
| 60796 | Type II diabetes mellitus with persistent proteinuria |
| 61021 | Diabetic digital retinopathy screening offered |
| 61071 | Type 2 diabetes mellitus with hypoglycaemic coma |
| 61122 | Diabetes mellitus induced by non-steroid drugs |
| 61210 | Adverse reaction to insulins and antidiabetic agents NOS |
| 61461 | Informed consent for diabetes national audit |
| 61470 | Diabetic monitoring - higher risk albumin excretion |
| 61523 | Other specified diabetes mellitus with neurological comps |
| 61557 | Diabetology D.V. requested |
| 61670 | Diab mellit insulin-glucose infus acute myocardial infarct |
| 62107 | Type II diabetes mellitus with gangrene |
| 62146 | Non-insulin-dependent diabetes mellitus with multiple comps |
| 62384 | O/E - right chronic diabetic foot ulcer |
| 62674 | Type 2 diabetes mellitus with mononeuropathy |
| 63357 | Diabetes mellitus, adult, + peripheral circulatory disorder |
| 63364 | [X] Adverse reaction to insulins |
| 63371 | Diabetes mellitus, adult, + other specified manifestation |
| 63412 | Diabetes clinical management plan |
| 63690 | Type 2 diabetes mellitus with gastroparesis |
| 63762 | Diabetes mellitus, adult onset, + unspecified complication |
| 64142 | Referral for diabetic retinopathy screening |
| 64283 | Other specified diabetes mellitus with unspecified comps |
| 64357 | Diabetes mellitus NOS with unspecified complication |
| 64384 | Diabetes mellitus in pregnancy/childbirth/puerperium NOS |
| 64446 | Insulin dependent diab mell with peripheral angiopathy |
| 64449 | Unspecified diabetes mellitus with multiple complications |
| 64571 | Type II diabetes mellitus with nephropathy |
| 64668 | Insulin treated Type II diabetes mellitus |
| 65025 | Diabetes mellitus NOS with peripheral circulatory disorder |
| 65062 | Diabetes mellitus NOS with ketoacidotic coma |
| 65171 | [D]Drug induced hyperglycaemia |
| 65267 | Type 2 diabetes mellitus with multiple complications |
| 65463 | High risk non proliferative diabetic retinopathy |
| 65616 | Insulin dependent diabetes mellitus with arthropathy |
| 65684 | [X] Adverse reaction to insulins and antidiabetic agents |
| 65704 | Type 2 diabetes mellitus with ulcer |
| 66475 | Diabetic monitoring - lower risk albumin excretion |
| 66675 | Malnutrition-related diabetes mellitus with coma |
| 66689 | [X] Adverse reaction to gliclazide |
| 66946 | Adverse reaction to tolazamide |
| 66965 | Type 2 diabetes mellitus with neuropathic arthropathy |
| 67212 | DM induced by non-steroid drugs without complication |
| 67635 | Diabetes mellitus - unspec whether in pregnancy/puerperium |
| 67664 | Education score - diabetes |
| 67853 | Diabetes mellitus, juvenile, + neurological manifestation |
| 67905 | Type II diabetes mellitus with neurological complications |
| 68517 | Insulin autoimmune syndrome |
| 68546 | Diabetes clinic satisfaction questionnaire |
| 68714 | Insulins and antidiabetic poisoning |
| 68792 | Diabetes mellitus, juvenile type, + unspecified complication |
| 68818 | DTSQ - Diabetes treatment satisfaction questionnaire |
| 68843 | Diabetes mellitus, adult onset, with ketoacidotic coma |
| 68928 | Adverse reaction to insulins and antidiabetic agents |
| 69124 | IDDM with peripheral circulatory disorder |
| 69163 | Referral to multidisciplinary diabetic clinic |
| 69278 | Non-insulin depend diabetes mellitus with diabetic cataract |
| 69748 | Diabetes mellitus, juvenile type, + ophthalmic manifestation |
| 70316 | Type 2 diabetes mellitus with ophthalmic complications |
| 70448 | Diabetes mellitus, juvenile +peripheral circulatory disorder |
| 70821 | Diabetes mellitus NOS with other specified manifestation |
| 71476 | [X] Adverse reaction to gliquidone |
| 71486 | Adverse reaction to chlorpropamide |
| 72320 | Non-insulin dependent diabetes mellitus with mononeuropathy |
| 72333 | Listed for Diabetology admissn |
| 72345 | Diabetes mellitus NOS with hyperosmolar coma |
| 72702 | Insulin dependent diabetes mellitus - poor control |
| 82474 | Referral to community diabetes specialist nurse |
| 83485 | Insulin dose changed |
| 83532 | Diabetes type 2 review |
| 85991 | Type II diabetes mellitus with persistent microalbuminuria |
| 89737 | Adverse reaction to glipizide |
| 91164 | CSQ - Diabetes clinic satisfaction questionnaire |
| 91646 | Type II diabetes mellitus with ulcer |
| 93380 | Cystic fibrosis related diabetes mellitus |
| 93390 | Attended DAFNE diabetes structured education programme |
| 93491 | DAFNE diabetes structured education programme completed |
| 93529 | DESMOND diabetes structured education programme completed |
| 93631 | XPERT diabetes structured education programme completed |
| 93657 | Referral to DESMOND diabetes structured education programme |
| 93704 | Referral to DAFNE diabetes structured education programme |
| 93727 | Type II diabetes mellitus with diabetic cataract |
| 93854 | Diabetes structured education programme declined |
| 93870 | Referral to XPERT diabetes structured education programme |
| 93875 | Insulin dependent diabetes mellitus with retinopathy |
| 93922 | Diabetes mellitus, juvenile type, with renal manifestation |
| 94011 | Attended XPERT diabetes structured education programme |
| 94067 | [X] Adverse reaction to glipzide |
| 94186 | Diabetes structured education programme completed |
| 94330 | Referral to diabetes special interest general practitioner |
| 94383 | Secondary diabetes mellitus without complication |
| 94699 | Diabetes treatment satisfaction questionnaire |
| 94777 | [V]Personal history of gestational diabetes mellitus |
| 94955 | Did not attend XPERT diabetes structured education programme |
| 94956 | Did not complete XPERT diabetes structured education program |
| 95093 | Did not complete DESMOND diabetes structured educat program |
| 95094 | Did not complete diabetes structured education programme |
| 95159 | Did not attend DESMOND diabetes structured education program |
| 95539 | Maternally inherited diabetes mellitus |
| 95553 | Did not attend diabetes structured education programme |
| 95636 | Latent autoimmune diabetes mellitus in adult |
| 95813 | Seen in multidisciplinary diabetic clinic |
| 95994 | Diabetic foot screen |
| 96010 | Insulin treatment initiated |

Diabetes mellitus - Product codes

| prodcode | prodname |
| --- | --- |
| 43950 | Humulin I KwikPen 100units/ml suspension for injection 3ml pre-filled pen (Eli Lilly and Company Ltd) |
| 10547 | Humulin Lente 100units/ml suspension for injection 10ml vials (Eli Lilly and Company Ltd) |
| 8390 | Gliquidone 30mg tablets |
| 5276 | Glimepiride 1mg tablets |
| 44250 | Metformin 500mg/5ml Oral solution (Hillcross Pharmaceuticals Ltd) |
| 1592 | Actrapid penfill 100 100iu/ml Penfill (Novo Nordisk Ltd) |
| 28588 | Hypurin Bovine Isophane 100units/ml suspension for injection 3ml cartridges (Wockhardt UK Ltd) |
| 2219 | Glibenclamide 2.5mg tablets |
| 36031 | Insulin isophane biphasic porcine 30/70 100units/ml suspension for injection 3ml cartridges |
| 7912 | Semi-Daonil 2.5mg tablets (Sanofi) |
| 43684 | Janumet 50mg/1000mg tablets (Merck Sharp & Dohme Ltd) |
| 7400 | Insulin glargine 100units/ml solution for injection 3ml pre-filled disposable devices |
| 41120 | Insulin isophane biphasic human 50/50 100units/ml suspension for injection 3ml pre-filled disposable devices |
| 17706 | Minodiab 2.5mg tablets (Pfizer Ltd) |
| 13622 | Hypurin porcine neutral 100unit/ml Injection (C P Pharmaceuticals Ltd) |
| 5953 | Insulin glargine 100iu/ml Injection |
| 10277 | Humulin M3 100units/ml suspension for injection 3ml cartridges (Eli Lilly and Company Ltd) |
| 3740 | Guar gum 90% granules |
| 18224 | Humalog 100units/ml solution for injection 10ml vials (Eli Lilly and Company Ltd) |
| 39598 | Metformin 1g modified-release tablets |
| 22945 | Insuman rapid 100iu/ml Injection (Aventis Pharma) |
| 10207 | Insulin isophane human 100units/ml suspension for injection 3ml cartridges |
| 39560 | Bolamyn SR 500mg tablets (Teva UK Ltd) |
| 14928 | Insulatard 100units/ml suspension for injection 10ml vials (Novo Nordisk Ltd) |
| 29953 | Apidra 100units/ml solution for injection 3ml OptiClik cartridges (Sanofi) |
| 30316 | Metformin with pioglitazone 850mg + 15mg Tablet |
| 34957 | Tolbutamide 500mg tablets (A A H Pharmaceuticals Ltd) |
| 10225 | Lantus 100units/ml solution for injection 3ml OptiClik cartridges (Sanofi) |
| 30236 | Isophane insulin 100iu/ml Injection |
| 13628 | Romozin 400mg Tablet (Glaxo Wellcome UK Ltd) |
| 10910 | Humaject m2 100iu/ml M2 pen (Eli Lilly and Company Ltd) |
| 12297 | Hypurin bovine neutral 100unit/ml Injection (C P Pharmaceuticals Ltd) |
| 2221 | Mixtard 30 NovoLet 100units/ml suspension for injection (Novo Nordisk Ltd) |
| 16142 | Insulin aspart 100units/ml solution for injection 3ml cartridges |
| 1588 | Actrapid 100iu/ml Injection (Novo Nordisk Ltd) |
| 11604 | Rosiglitazone 1mg / Metformin 500mg tablets |
| 16602 | Calabren 2.5mg Tablet (Berk Pharmaceuticals Ltd) |
| 10572 | Insulin soluble bovine 100unit/ml Injection |
| 26118 | Dimelor 500mg Tablet (Eli Lilly and Company Ltd) |
| 36066 | Insulin isophane bovine 100units/ml suspension for injection 3ml cartridges |
| 21892 | Diaglyk 80mg tablets (Ashbourne Pharmaceuticals Ltd) |
| 8118 | Humaject i 100iu/ml Pen (Eli Lilly and Company Ltd) |
| 1847 | Chlorpropamide 250mg tablets |
| 8203 | Penmix 50/50 100iu/ml Penfill (Novo Nordisk Ltd) |
| 1965 | Tolbutamide 500mg tablets |
| 21583 | Apidra 100units/ml solution for injection 3ml pre-filled OptiSet pen (Sanofi) |
| 32 | Gliclazide 80mg tablets |
| 14619 | Insulin isophane biphasic porcine 30/70 100units/ml suspension for injection 1.5ml cartridges |
| 31205 | Insuman Comb 50 100units/ml suspension for injection 3ml pre-filled OptiSet pen (Sanofi) |
| 22697 | Insulin isophane biphasic human 50/50 100units/ml suspension for injection 1.5ml cartridges |
| 18592 | Insulin soluble bovine 100units/ml solution for injection 10ml vials |
| 26218 | Calabren 5mg Tablet (Berk Pharmaceuticals Ltd) |
| 15374 | Gliclazide 40mg/5ml oral suspension |
| 34917 | Metformin 500mg tablets (Teva UK Ltd) |
| 7318 | Humalog 100units/ml solution for injection 3ml cartridges (Eli Lilly and Company Ltd) |
| 6958 | Levemir FlexPen 100units/ml solution for injection 3ml pre-filled pen (Novo Nordisk Ltd) |
| 44251 | Insulin zinc suspension mixed porcine 100unit/ml Injection |
| 35468 | Insuman Basal 100units/ml suspension for injection 5ml vials (Sanofi) |
| 11695 | Diamicron 30mg MR tablets (Servier Laboratories Ltd) |
| 41558 | Glibenclamide 5mg tablets (Teva UK Ltd) |
| 10067 | Insulin biphasic aspart human pyr 30:70; 100 units/ml Injection |
| 28101 | Insulin glulisine 100units/ml solution for injection 10ml vials |
| 27396 | Insulin soluble porcine 100units/ml solution for injection 10ml vials |
| 4199 | Humulin m1 100unit/ml M1 injection (Eli Lilly and Company Ltd) |
| 9376 | Insulin zinc suspension crystalline human pyr 100unit/ml long acting Injection |
| 21424 | Glibenclamide 5mg/5ml oral suspension |
| 45215 | Gliclazide 80mg Tablet (Neo Laboratories Ltd) |
| 10915 | Humaject m1 100iu/ml M1 pen (Eli Lilly and Company Ltd) |
| 18931 | Insulin zinc crystalline human 100units/ml suspension for injection 10ml vials |
| 31212 | Gliclazide 80mg tablets (Actavis UK Ltd) |
| 11284 | Amaryl 4mg tablets (Zentiva) |
| 24002 | Insuman Comb 25 100units/ml suspension for injection 5ml vials (Sanofi) |
| 9565 | HumaJect S Pen 100units/ml solution for injection (Eli Lilly and Company Ltd) |
| 9737 | Insulatard innolet 100iu/ml Injection (Novo Nordisk Ltd) |
| 21554 | Insuman comb 50 100iu/ml Injection (Aventis Pharma) |
| 26060 | Insulin lispro 100units/ml solution for injection 10ml vials |
| 11107 | Humulin m4 100unit/ml M4 injection (Eli Lilly and Company Ltd) |
| 11609 | Metformin with rosiglitazone 500mg + 1mg Tablet |
| 35149 | Exenatide 10micrograms/0.04ml solution for injection 2.4ml pre-filled disposable devices |
| 11737 | Metformin with rosiglitazone 1000mg + 4mg Tablet |
| 34004 | Metformin 500mg tablets (IVAX Pharmaceuticals UK Ltd) |
| 43065 | Gliclazide 40mg tablets |
| 10887 | Penmix 40/60 100iu/ml Penfill (Novo Nordisk Ltd) |
| 34507 | Glibenclamide 2.5mg tablets (Wockhardt UK Ltd) |
| 16682 | Tempulin 100unit/ml Injection (Knoll Ltd) |
| 20889 | Actos 30mg tablets (Takeda UK Ltd) |
| 5214 | Insulin lispro 100units/ml solution for injection 1.5ml cartridges |
| 735 | Metformin 100mg/ml Oral solution |
| 548 | Pioglitazone 15mg tablets |
| 3551 | Mixtard 20 penfill 100 100iu/ml Penfill (Novo Nordisk Ltd) |
| 38551 | Eucreas 50mg/1000mg tablets (Novartis Pharmaceuticals UK Ltd) |
| 5250 | Insulin biphasic lispro human prb 25:75; 100 units/ml Injection |
| 22155 | Humaject m5 100iu/ml M5 pen (Eli Lilly and Company Ltd) |
| 37874 | Vildagliptin 50mg / Metformin 850mg tablets |
| 11056 | Insulin biphasic isophane human pyr 30:70; 100 units/ml Injection |
| 5933 | Mixtard 50 NovoLet 100units/ml suspension for injection (Novo Nordisk Ltd) |
| 35144 | Byetta 5micrograms/0.02ml solution for injection 1.2ml pre-filled disposable devices (Bristol-Myers Squibb Pharmaceuticals Ltd) |
| 40693 | Liraglutide 6mg/ml solution for injection 3ml pre-filled disposable devices |
| 20995 | Hypurin Porcine 30/70 Mix 100units/ml suspension for injection 3ml cartridges (Wockhardt UK Ltd) |
| 7166 | Glucophage 500mg tablets (Merck Serono Ltd) |
| 547 | Glipizide 2.5mg tablets |
| 8034 | Diabinese 100mg Tablet (Pfizer Ltd) |
| 5892 | NovoRapid FlexPen 100units/ml solution for injection 3ml pre-filled pen (Novo Nordisk Ltd) |
| 7349 | Actrapid 100units/ml solution for injection 10ml vials (Novo Nordisk Ltd) |
| 40110 | Glucophage 500mg oral powder sachets (Merck Serono Ltd) |
| 9865 | Repaglinide 500microgram tablets |
| 1649 | Human actraphane 100iu/ml Injection (Novo Nordisk Ltd) |
| 34697 | Metformin 850mg tablets (Wockhardt UK Ltd) |
| 21564 | Gliclazide 80mg tablets (Wockhardt UK Ltd) |
| 9707 | Repaglinide 1mg tablets |
| 15232 | Avandia 8mg tablets (GlaxoSmithKline UK Ltd) |
| 12259 | Glibornuride 25mg Tablet |
| 17343 | Gliclazide 80mg tablets (A A H Pharmaceuticals Ltd) |
| 22058 | Pur-in mix 15/85 Injection (C P Pharmaceuticals Ltd) |
| 11601 | Rosiglitazone 2mg / Metformin 500mg tablets |
| 4307 | Guarina Sachets (Norgine Pharmaceuticals Ltd) |
| 7325 | Avandamet 4mg/1000mg tablets (GlaxoSmithKline UK Ltd) |
| 14938 | Insulin soluble bovine cartridge 100unit/ml Solution for injection |
| 2459 | Pork Mixtard 30 100units/ml suspension for injection 10ml vials (Novo Nordisk Ltd) |
| 25678 | Glucamet 500mg Tablet (Opus Pharmaceuticals Ltd) |
| 14649 | Insulin biphasic isophane human pyr 10:90; 100 units/ml Injection |
| 39149 | Galvus 50mg tablets (Novartis Pharmaceuticals UK Ltd) |
| 16160 | Humulin M3 Pen 100units/ml suspension for injection 3ml pre-filled pen (Eli Lilly and Company Ltd) |
| 41431 | Onglyza 5mg tablets (Bristol-Myers Squibb Pharmaceuticals Ltd) |
| 2929 | Mixtard 30 100iu/ml GE injection (Novo Nordisk Ltd) |
| 34676 | Glibenclamide 2.5mg tablets (A A H Pharmaceuticals Ltd) |
| 43465 | Zicron 40mg tablets (Bristol Laboratories Ltd) |
| 41593 | Glibenclamide 2.5mg tablets (Teva UK Ltd) |
| 27461 | Insuman Basal 100units/ml suspension for injection 3ml cartridges (Sanofi) |
| 7409 | Amaryl 3mg tablets (Zentiva) |
| 17731 | Penmix 50/50 100iu/ml Injection (Novo Nordisk Ltd) |
| 33966 | Insulatard 100unit/ml Injection (Novo Nordisk Ltd) |
| 9699 | Pioglitazone 30mg tablets |
| 35260 | Levemir InnoLet 100units/ml solution for injection 3ml pre-filled pen (Novo Nordisk Ltd) |
| 6965 | Levemir Penfill 100units/ml solution for injection 3ml cartridges (Novo Nordisk Ltd) |
| 40233 | Metformin 1g oral powder sachets sugar free |
| 34135 | Metformin 500mg Tablet (M & A Pharmachem Ltd) |
| 479 | Acarbose 50mg tablets |
| 24800 | Hypurin Porcine 30/70 Mix 100units/ml suspension for injection 10ml vials (Wockhardt UK Ltd) |
| 11337 | NovoRapid Novolet 100units/ml solution for injection (Novo Nordisk Ltd) |
| 6061 | Novomix 30 30/70 100units/ml Injection (Novo Nordisk Ltd) |
| 33232 | Insulin isophane biphasic human 50/50 100units/ml suspension for injection 5ml vials |
| 38355 | Metformin 750mg modified-release tablets |
| 2812 | Mixtard 40 NovoLet 100units/ml suspension for injection (Novo Nordisk Ltd) |
| 4760 | Humulin i 100unit/ml Injection (Eli Lilly and Company Ltd) |
| 469 | Rosiglitazone 4mg tablets |
| 14313 | Insulin lispro 100units/ml solution for injection 3ml cartridges |
| 35150 | Byetta 10micrograms/0.04ml solution for injection 2.4ml pre-filled disposable devices (Bristol-Myers Squibb Pharmaceuticals Ltd) |
| 1594 | Actrapid NovoLet 100units/ml solution for injection (Novo Nordisk Ltd) |
| 2454 | Mixtard 30 penfill 100 100iu/ml Penfill (Novo Nordisk Ltd) |
| 15955 | Starlix 120mg tablets (Novartis Pharmaceuticals UK Ltd) |
| 27125 | Starlix 180mg tablets (Novartis Pharmaceuticals UK Ltd) |
| 1806 | Penmix 30/70 100iu/ml Penfill (Novo Nordisk Ltd) |
| 27280 | Insulin isophane biphasic porcine 30/70 100units/ml suspension for injection 10ml vials |
| 5627 | Gliclazide 30mg modified-release tablets |
| 34399 | Gliclazide 80mg tablets (IVAX Pharmaceuticals UK Ltd) |
| 14918 | Humulin I 100units/ml suspension for injection 10ml vials (Eli Lilly and Company Ltd) |
| 13729 | Insulin isophane human emp 100unit/ml Injection |
| 25736 | Insulin isophane biphasic human 10/90 100units/ml suspension for injection 3ml cartridges |
| 8322 | Insulin zinc suspension mixed human pyr 100unit/ml Injection |
| 14301 | Insulin detemir 100units/ml solution for injection 3ml cartridges |
| 6337 | Glimepiride 3mg tablets |
| 1843 | Pork Insulatard 100units/ml suspension for injection 10ml vials (Novo Nordisk Ltd) |
| 16700 | Insulin zinc mixed bovine vial 100unit/ml Sterile suspension injection |
| 34598 | Metformin 500mg tablets (Generics (UK) Ltd) |
| 40642 | Victoza 6mg/ml solution for injection 3ml pre-filled pen (Novo Nordisk Ltd) |
| 23 | Metformin 500mg tablets |
| 36853 | Lantus 100units/ml solution for injection 3ml pre-filled SoloStar pen (Sanofi) |
| 26403 | Pur-in mix 25/75 Injection (C P Pharmaceuticals Ltd) |
| 29567 | Insulin aspart 100units/ml solution for injection 10ml vials |
| 10484 | Penmix 20/80 Penfill (Novo Nordisk Ltd) |
| 14339 | Hypurin Bovine Neutral 100units/ml solution for injection 10ml vials (Wockhardt UK Ltd) |
| 23231 | Hypurin Bovine Neutral 100units/ml solution for injection 3ml cartridges (Wockhardt UK Ltd) |
| 33087 | Metformin 500mg tablets (Actavis UK Ltd) |
| 1254 | Glibenclamide 5mg tablets |
| 18593 | Humalog Mix50 100units/ml suspension for injection 3ml cartridges (Eli Lilly and Company Ltd) |
| 322 | Humalog 100units/ml solution for injection 1.5ml cartridges (Eli Lilly and Company Ltd) |
| 14505 | Insulin protamine zinc bovine 100units/ml suspension for injection 10ml vials |
| 7266 | Lantus 100units/ml solution for injection 3ml cartridges (Sanofi) |
| 5678 | Nateglinide 120mg tablets |
| 3550 | Mixtard 40 penfill 100 100iu/ml Penfill (Novo Nordisk Ltd) |
| 26098 | Hypurin Porcine Neutral 100units/ml solution for injection 10ml vials (Wockhardt UK Ltd) |
| 24848 | Glymidine sodium 500mg Tablet |
| 34031 | Monotard mc 100unit/ml Injection (Novo Nordisk Ltd) |
| 16152 | Insulin isophane biphasic human 30/70 100units/ml suspension for injection 3ml cartridges |
| 28442 | Insulin glulisine 100unit/ml Solution for injection |
| 15484 | Insulin isophane bovine 100units/ml suspension for injection 1.5ml cartridges |
| 6209 | NovoRapid 100units/ml solution for injection 10ml vials (Novo Nordisk Ltd) |
| 18590 | Insulin isophane bovine 100units/ml suspension for injection 10ml vials |
| 38400 | Glucophage SR 750mg tablets (Merck Serono Ltd) |
| 34323 | Metformin 500mg tablets (A A H Pharmaceuticals Ltd) |
| 12818 | Human Mixtard 50 100units/ml suspension for injection 10ml vials (Novo Nordisk Ltd) |
| 17698 | Minodiab 5mg tablets (Pfizer Ltd) |
| 6447 | Insulin aspart human pyr 100 iu/ml Injection |
| 5227 | Rosiglitazone 8mg tablets |
| 35701 | Insulin lispro biphasic 50/50 100units/ml suspension for injection 3ml pre-filled disposable devices |
| 10001 | Humalog Mix50 Pen 100units/ml suspension for injection 3ml pre-filled pen (Eli Lilly and Company Ltd) |
| 7284 | Amaryl 2mg tablets (Zentiva) |
| 21422 | Insulin isophane biphasic human 40/60 100units/ml suspension for injection 3ml cartridges |
| 14930 | Hypurin Porcine Neutral 100units/ml solution for injection 3ml cartridges (Wockhardt UK Ltd) |
| 35253 | Insuman Comb 50 100units/ml suspension for injection 3ml cartridges (Sanofi) |
| 7350 | Insulin isophane porcine 100units/ml suspension for injection 10ml vials |
| 21489 | Tolanase 250mg Tablet (Pharmacia Ltd) |
| 33562 | Duclazide 80mg Tablet (Dumex Ltd) |
| 36356 | Insulin human 3mg inhalation powder blisters |
| 4198 | Humulin m3 100unit/ml M3 injection (Eli Lilly and Company Ltd) |
| 34706 | Glibenclamide 2.5mg tablets (IVAX Pharmaceuticals UK Ltd) |
| 27969 | Glymese 250mg Tablet (DDSA Pharmaceuticals Ltd) |
| 17809 | Humaject m4 100iu/ml M4 pen (Eli Lilly and Company Ltd) |
| 20287 | Actos 15mg tablets (Takeda UK Ltd) |
| 31465 | Exubera 1mg inhalation powder blisters (Pfizer Ltd) |
| 11760 | Metformin with rosiglitazone 1000mg + 2mg Tablet |
| 45821 | Onglyza 2.5mg tablets (Bristol-Myers Squibb Pharmaceuticals Ltd) |
| 7610 | Glucophage 850mg tablets (Merck Serono Ltd) |
| 4862 | Diabetamide 2.5mg tablets (Ashbourne Pharmaceuticals Ltd) |
| 26498 | Insulin zinc suspension mixed bovine and porcine 100unit/ml Injection |
| 40007 | Glucophage 1000mg oral powder sachets (Merck Serono Ltd) |
| 25479 | Insulin soluble porcine 100units/ml solution for injection 3ml cartridges |
| 1587 | Monotard 100units/ml suspension for injection 10ml vials (Novo Nordisk Ltd) |
| 10245 | Mixtard 10 Penfill 100units/ml suspension for injection 3ml cartridges (Novo Nordisk Ltd) |
| 41559 | Glibenclamide 5mg tablets (A A H Pharmaceuticals Ltd) |
| 41959 | Penject 100unit/ml Injection device (Hypoguard Ltd) |
| 5636 | Glipizide 5mg tablets |
| 10259 | Insulin glargine 100units/ml solution for injection 10ml vials |
| 14644 | Insulin biphasic isophane human prb 20:80; 100 units/ml Injection |
| 21110 | Insulin biphasic isophane human prb 50:50; 100 units/ml Injection |
| 23992 | Insuman Basal 100units/ml suspension for injection 3ml pre-filled OptiSet pen (Sanofi) |
| 43953 | Insulin lispro biphasic 25/75 100units/ml suspension for injection 10ml vials |
| 39086 | Humalog Mix50 KwikPen 100units/ml suspension for injection 3ml pre-filled pen (Eli Lilly and Company Ltd) |
| 13819 | Hypurin Porcine Isophane 100units/ml suspension for injection 1.5ml cartridges (C P Pharmaceuticals Ltd) |
| 7267 | NovoMix 30 Penfill 100units/ml suspension for injection 3ml cartridges (Novo Nordisk Ltd) |
| 11321 | NovoNorm 1mg tablets (Novo Nordisk Ltd) |
| 43991 | Humulin M3 KwikPen 100units/ml suspension for injection 3ml pre-filled pen (Eli Lilly and Company Ltd) |
| 10051 | Pioglitazone 45mg tablets |
| 34802 | Glipizide 5mg tablets (IVAX Pharmaceuticals UK Ltd) |
| 36194 | Insulin isophane biphasic human 25/75 100units/ml suspension for injection 3ml cartridges |
| 21232 | Insulin isophane biphasic human 30/70 100units/ml suspension for injection 10ml vials |
| 24846 | Pur-in neutral 100unit/ml Injection (C P Pharmaceuticals Ltd) |
| 19336 | Tolazamide 100mg Tablet |
| 14362 | Insulin lispro 100units/ml solution for injection 3ml pre-filled disposable devices |
| 1842 | Pork velosulin 100unit/ml Injection (Novo Nordisk Ltd) |
| 13277 | Mixtard 50 Penfill 100units/ml suspension for injection 3ml cartridges (Novo Nordisk Ltd) |
| 25636 | Libanil 2.5mg Tablet (Approved Prescription Services Ltd) |
| 7393 | Insulin glargine 100units/ml solution for injection 3ml cartridges |
| 8976 | Euglucon 2.5mg tablets (Aventis Pharma) |
| 31146 | Metsol 500mg/5ml oral solution (Kappin Ltd) |
| 35462 | Januvia 100mg tablets (Merck Sharp & Dohme Ltd) |
| 27501 | Orabet 500mg Tablet (Lagap) |
| 37902 | Vildagliptin 50mg / Metformin 1g tablets |
| 31474 | Libanil 5mg Tablet (Approved Prescription Services Ltd) |
| 44473 | Edicil MR 30mg tablets (Ratiopharm UK Ltd) |
| 7695 | Guarem Sachets (Shire Pharmaceuticals Ltd) |
| 14925 | Insulin isophane human vial 100unit/ml Sterile suspension injection |
| 23099 | Insulin aspart biphasic 30/70 100units/ml suspension for injection 3ml pre-filled disposable devices |
| 7537 | Humulin Zn 100units/ml suspension for injection 10ml vials (Eli Lilly and Company Ltd) |
| 5989 | Nateglinide 180mg tablets |
| 40425 | Nazdol MR 30mg tablets (Teva UK Ltd) |
| 11483 | Nateglinide 60mg tablets |
| 28183 | Hypurin Porcine Isophane 100units/ml suspension for injection 10ml vials (Wockhardt UK Ltd) |
| 7319 | Mixtard 20 Penfill 100units/ml suspension for injection 3ml cartridges (Novo Nordisk Ltd) |
| 25735 | Insulin isophane biphasic human 20/80 100units/ml suspension for injection 3ml cartridges |
| 19658 | Glurenorm 30mg tablets (Sanofi) |
| 34097 | Human initard 50/50 100unit/ml Injection (Novo Nordisk Ltd) |
| 41204 | Saxagliptin 5mg tablets |
| 36774 | Prandin 1mg tablets (Novo Nordisk Ltd) |
| 14345 | Apidra 100units/ml solution for injection 3ml cartridges (Sanofi) |
| 12654 | Insulin soluble human prb 100unit/ml Injection |
| 14330 | Insulin detemir 100units/ml solution for injection 3ml pre-filled disposable devices |
| 17336 | Novopen 100unit/ml Injection device (Novo Nordisk Ltd) |
| 9503 | Hypurin Bovine Protamine Zinc 100units/ml suspension for injection 10ml vials (Wockhardt UK Ltd) |
| 30686 | Insulin isophane porcine 100units/ml suspension for injection 3ml cartridges |
| 9341 | Insulin biphasic isophane human prb 30:70; 100 units/ml Injection |
| 36856 | Gliclazide 80mg tablets (Sandoz Ltd) |
| 14164 | Avandamet 2mg/1000mg tablets (GlaxoSmithKline UK Ltd) |
| 21590 | Insulin glulisine 100units/ml solution for injection 3ml pre-filled disposable devices |
| 24993 | Insuman Comb 25 100units/ml suspension for injection 3ml cartridges (Sanofi) |
| 34742 | Metformin 850mg tablets (Teva UK Ltd) |
| 10427 | Tolazamide 250mg Tablet |
| 31258 | Insulin lispro biphasic 25/75 100units/ml suspension for injection 3ml pre-filled disposable devices |
| 10243 | Humalog Mix25 100units/ml suspension for injection 3ml cartridges (Eli Lilly and Company Ltd) |
| 34932 | Gliclazide 80mg tablets (Genus Pharmaceuticals Ltd) |
| 28096 | Insulin isophane biphasic human 50/50 100units/ml suspension for injection 3ml cartridges |
| 36513 | Velosulin cartridge 100unit/ml Injection (Novo Nordisk Ltd) |
| 16129 | Insulin soluble human 100units/ml solution for injection 3ml cartridges |
| 5353 | Glimepiride 2mg tablets |
| 44480 | Insuman Comb 25 100units/ml suspension for injection 3ml pre-filled SoloStar pen (Sanofi) |
| 5845 | Mixtard 30 InnoLet 100units/ml suspension for injection 3ml pre-filled pen (Novo Nordisk Ltd) |
| 11366 | NovoNorm 2mg tablets (Novo Nordisk Ltd) |
| 34504 | Metformin 500mg tablets (Wockhardt UK Ltd) |
| 34020 | Metformin 850mg tablets (IVAX Pharmaceuticals UK Ltd) |
| 6855 | Avandamet 2mg/500mg tablets (GlaxoSmithKline UK Ltd) |
| 7771 | Human protaphane penfill 100 100unit/ml Penfill (Novo Nordisk Ltd) |
| 33167 | Insulin biphasic isophane human crb 25:75; 100 units/ml Injection |
| 18461 | Insulin zinc mixed human 100units/ml suspension for injection 10ml vials |
| 7231 | Mixtard 30 Penfill 100units/ml suspension for injection 3ml cartridges (Novo Nordisk Ltd) |
| 35251 | Exenatide 5micrograms/0.02ml solution for injection 1.2ml pre-filled disposable devices |
| 4163 | Rapitard MC 100unit/ml Injection (Novo Nordisk Ltd) |
| 7332 | Amaryl 1mg tablets (Zentiva) |
| 42395 | Humalog Mix25 100units/ml suspension for injection 10ml vials (Eli Lilly and Company Ltd) |
| 9105 | Glucobay 100mg tablets (Bayer Plc) |
| 3439 | Penmix 10/90 Pen (Novo Nordisk Ltd) |
| 19877 | Insulin aspart 100units/ml solution for injection 3ml pre-filled disposable devices |
| 16044 | Glucophage SR 500mg tablets (Merck Serono Ltd) |
| 38986 | Humalog KwikPen 100units/ml solution for injection 3ml pre-filled pen (Eli Lilly and Company Ltd) |
| 4784 | Lentard mc 100unit/ml Injection (Novo Nordisk Ltd) |
| 41834 | Insulin zinc suspension lente 100iu/ml Injection (Celltech Pharma Europe Ltd) |
| 29326 | Glipizide 5mg tablets (Generics (UK) Ltd) |
| 6057 | Lantus 100iu/ml Injection (Aventis Pharma) |
| 23945 | Starlix 60mg tablets (Novartis Pharmaceuticals UK Ltd) |
| 1840 | Humulin s 100unit/ml Injection (Eli Lilly and Company Ltd) |
| 29939 | Gliclazide 80mg tablets (Generics (UK) Ltd) |
| 1964 | Diamicron 80mg tablets (Servier Laboratories Ltd) |
| 8895 | Initard 50/50 100unit/ml Injection (Novo Nordisk Ltd) |
| 4715 | Humalog mix 25 25/75 100units/ml Injection (Eli Lilly and Company Ltd) |
| 18220 | Pioglitazone 15mg / Metformin 850mg tablets |
| 35022 | Sitagliptin 100mg tablets |
| 21347 | Penmix 40/60 100iu/ml Injection (Novo Nordisk Ltd) |
| 13837 | Insulin biphasic isophane human prb 10:90; 100 units/ml Injection |
| 5255 | Mixtard 10 penfill 100 100iu/ml Penfill (Novo Nordisk Ltd) |
| 30460 | Malix 5mg Tablet (Lagap) |
| 1593 | Insulatard penfill 100 100iu/ml Penfill (Novo Nordisk Ltd) |
| 17580 | Avandamet 1mg/500mg tablets (GlaxoSmithKline UK Ltd) |
| 30819 | Insuman Comb 15 100units/ml suspension for injection 3ml pre-filled OptiSet pen (Sanofi) |
| 28185 | Insulin lispro biphasic 25/75 100units/ml suspension for injection 3ml cartridges |
| 14340 | Hypurin Bovine Isophane 100units/ml suspension for injection 10ml vials (Wockhardt UK Ltd) |
| 15199 | Insuman comb 25 100iu/ml Injection (Aventis Pharma) |
| 11080 | Insulin isophane human prb 100iu/ml Injection |
| 20422 | Insuman comb 15 100iu/ml Injection (Aventis Pharma) |
| 15710 | Insulin soluble human emp 100unit/ml Injection |
| 37875 | Vildagliptin 50mg tablets |
| 21395 | Insulin biphasic isophane human pyr 40:60; 100 units/ml Injection |
| 45581 | Metabet SR 500mg tablets (Morningside Healthcare Ltd) |
| 11316 | NovoNorm 500microgram tablets (Novo Nordisk Ltd) |
| 21374 | Insulin biphasic isophane human prb 40:60; 100 units/ml Injection |
| 42790 | Gliclazide 80mg Tablet (Merck Generics (UK) Ltd) |
| 1805 | Mixtard 30/70 100unit/ml Injection (Novo Nordisk Ltd) |
| 7375 | Rosiglitazone 4mg / Metformin 1g tablets |
| 44738 | Niddaryl 1mg tablets (Dee Pharmaceuticals Ltd) |
| 10244 | Mixtard 40 Penfill 100units/ml suspension for injection 3ml cartridges (Novo Nordisk Ltd) |
| 36146 | Insulin lispro biphasic 50/50 100units/ml suspension for injection 3ml cartridges |
| 11055 | Insulin biphasic isophane human pyr 20:80; 100 units/ml Injection |
| 14270 | Humalog Mix25 Pen 100units/ml suspension for injection 3ml pre-filled pen (Eli Lilly and Company Ltd) |
| 45158 | Insuman Comb 15 100units/ml suspension for injection 3ml cartridges (Sanofi) |
| 4129 | Insulin soluble porcine 100units/ml solution for injection 1.5ml cartridges |
| 22983 | Insuman Rapid 100units/ml solution for injection 3ml cartridges (Sanofi) |
| 7237 | Lantus 100units/ml solution for injection 3ml pre-filled OptiSet pen (Sanofi) |
| 5021 | NovoRapid Penfill 100units/ml solution for injection 3ml cartridges (Novo Nordisk Ltd) |
| 7300 | Mixtard 30 100units/ml suspension for injection 10ml vials (Novo Nordisk Ltd) |
| 33674 | Metformin 850mg tablets (A A H Pharmaceuticals Ltd) |
| 31077 | Competact 15mg/850mg tablets (Takeda UK Ltd) |
| 12299 | Semitard mc 100unit/ml Injection (Novo Nordisk Ltd) |
| 13331 | Euglucon 5mg tablets (Sanofi) |
| 5501 | Insuman basal 100iu/ml Injection (Aventis Pharma) |
| 5174 | Acarbose 100mg tablets |
| 5316 | Glimepiride 4mg tablets |
| 12245 | Glutril 25mg Tablet (Roche Products Ltd) |
| 22145 | Tolanase 100mg Tablet (Pharmacia Ltd) |
| 7793 | HumaJect M3 Pen 100units/ml suspension for injection (Eli Lilly and Company Ltd) |
| 10175 | Insulin isophane human 100units/ml suspension for injection 1.5ml cartridges |
| 7744 | Daonil 5mg tablets (Sanofi) |
| 4247 | Insulin isophane porcine 100units/ml suspension for injection 1.5ml cartridges |
| 39203 | Eucreas 50mg/850mg tablets (Novartis Pharmaceuticals UK Ltd) |
| 8841 | Humulin M5 100units/ml suspension for injection 10ml vials (Eli Lilly and Company Ltd) |
| 42161 | Orabet 500mg Tablet (Sandoz Ltd) |
| 4093 | Humulin M2 100units/ml suspension for injection 3ml cartridges (Eli Lilly and Company Ltd) |
| 24593 | Neutral insulin bovine 100unit/ml Injection |
| 7772 | Human protaphane 100unit/ml Injection (Novo Nordisk Ltd) |
| 7228 | NovoMix 30 FlexPen 100units/ml suspension for injection 3ml pre-filled pen (Novo Nordisk Ltd) |
| 34836 | Metformin 850mg tablets (Actavis UK Ltd) |
| 1595 | Insulatard NovoLet 100units/ml suspension for injection (Novo Nordisk Ltd) |
| 11610 | Metformin with rosiglitazone 500mg + 2mg Tablet |
| 1886 | Insulatard 100iu/ml GE injection (Novo Nordisk Ltd) |
| 24795 | Insulin aspart biphasic 30/70 100units/ml suspension for injection 3ml cartridges |
| 43270 | Metformin 500mg/5ml oral solution sugar free (Rosemont Pharmaceuticals Ltd) |
| 7402 | Lantus 100units/ml solution for injection 10ml vials (Sanofi) |
| 27177 | Insulin biphasic lispro human prb 50:50; 100 units/ml Injection |
| 25133 | Insuman Comb 25 100units/ml suspension for injection 3ml pre-filled OptiSet pen (Sanofi) |
| 30209 | Actrapid mc 100unit/ml Injection (Arun Products Ltd) |
| 10184 | Insulin detemir 100 iu/ml Solution for injection |
| 12638 | Insulin soluble human pyr 100unit/ml Injection |
| 46001 | Insuman Basal 100units/ml suspension for injection 3ml pre-filled SoloStar pen (Sanofi) |
| 13516 | Hypurin bovine isophane 100unit/ml Injection (C P Pharmaceuticals Ltd) |
| 36920 | Apidra 100units/ml solution for injection 3ml pre-filled SoloStar pen (Sanofi) |
| 1844 | Ultratard 100units/ml suspension for injection 10ml vials (Novo Nordisk Ltd) |
| 22858 | Acetohexamide 500mg tablets |
| 5891 | Insulatard FlexPen 100units/ml suspension for injection (Novo Nordisk Ltd) |
| 23993 | Insuman Rapid 100units/ml solution for injection 3ml pre-filled OptiSet pen (Sanofi) |
| 5621 | Glucobay 50mg tablets (Bayer Plc) |
| 9748 | Repaglinide 2mg tablets |
| 17712 | Hypurin Bovine Lente 100units/ml suspension for injection 10ml vials (Wockhardt UK Ltd) |
| 93 | Metformin 850mg tablets |
| 14357 | Humulin I 100units/ml suspension for injection 3ml cartridges (Eli Lilly and Company Ltd) |
| 36355 | Insulin human 1mg inhalation powder blisters |
| 11717 | Rosiglitazone 2mg / Metformin 1g tablets |
| 2456 | Mixtard 10 NovoLet 100units/ml suspension for injection (Novo Nordisk Ltd) |
| 13416 | Insulin biphasic 100 units/ml Injection |
| 4706 | Velosulin 100units/ml solution for injection 10ml vials (Novo Nordisk Ltd) |
| 44378 | Insulin isophane biphasic human 25/75 100units/ml suspension for injection 3ml pre-filled disposable devices |
| 14933 | Hypurin Porcine Isophane 100units/ml suspension for injection 3ml cartridges (Wockhardt UK Ltd) |
| 9662 | Avandia 4mg tablets (GlaxoSmithKline UK Ltd) |
| 12513 | Glibenese 5mg tablets (Pfizer Ltd) |
| 10264 | Humalog Pen 100units/ml solution for injection 3ml pre-filled pen (Eli Lilly and Company Ltd) |
| 12035 | Insulin zinc mixed bovine 100units/ml suspension for injection 10ml vials |
| 14944 | Humulin S 100units/ml solution for injection 3ml cartridges (Eli Lilly and Company Ltd) |
| 15961 | Insulin isophane human crb 100iu/ml Injection |
| 43619 | Metformin 1g / Sitagliptin 50mg tablets |
| 38422 | Isophane 100iu/ml Injection (Celltech Pharma Europe Ltd) |
| 8168 | Diabinese 250mg Tablet (Pfizer Ltd) |
| 42954 | Insulin isophane biphasic human 25/75 100units/ml suspension for injection 5ml vials |
| 12897 | Guar gum 5g granules sachets sugar free |
| 9521 | Pork Actrapid 100units/ml solution for injection 10ml vials (Novo Nordisk Ltd) |
| 27614 | Penmix 30/70 100iu/ml Injection (Novo Nordisk Ltd) |
| 12455 | Rastinon 500mg Tablet (Hoechst Marion Roussel) |
| 27402 | Insulin soluble human 100units/ml solution for injection 10ml vials |
| 36430 | Insulin soluble human 100units/ml solution for injection 3ml pre-filled disposable devices |
| 14290 | Insulatard Penfill 100units/ml suspension for injection 3ml cartridges (Novo Nordisk Ltd) |
| 33673 | Tolbutamide 500mg tablets (Actavis UK Ltd) |
| 28708 | Malix 2.5mg Tablet (Lagap) |
| 35561 | Prandin 2mg tablets (Novo Nordisk Ltd) |
| 19491 | Apidra 100units/ml solution for injection 10ml vials (Sanofi) |
| 39729 | Glucophage SR 1000mg tablets (Merck Serono Ltd) |
| 45775 | Saxagliptin 2.5mg tablets |
| 25812 | Insulin isophane human 100units/ml suspension for injection 3ml pre-filled disposable devices |
| 26621 | Insulin soluble human crb 100iu/ml Injection |
| 14299 | Insulin glulisine 100units/ml solution for injection 3ml cartridges |
| 34563 | Glibenclamide 5mg tablets (Wockhardt UK Ltd) |
| 36948 | Prandin 0.5mg tablets (Novo Nordisk Ltd) |
| 9618 | Hypurin Porcine 30/70 Mix 100units/ml suspension for injection 1.5ml cartridges (C P Pharmaceuticals Ltd) |
| 3396 | Penmix 10/90 Penfill (Novo Nordisk Ltd) |
| 31467 | Exubera 3mg inhalation powder blisters (Pfizer Ltd) |
| 19513 | Humulin M3 100units/ml suspension for injection 10ml vials (Eli Lilly and Company Ltd) |
| 21832 | Diabetamide 5mg tablets (Ashbourne Pharmaceuticals Ltd) |
| 26258 | Glucamet 850mg Tablet (Opus Pharmaceuticals Ltd) |
| 7048 | Metformin 500mg modified-release tablets |
| 21235 | Humulin S 100units/ml solution for injection 10ml vials (Eli Lilly and Company Ltd) |
| 11946 | Tolbutamide 50mg/ml Injection |
| 40365 | Glimepiride 1mg tablets (Actavis UK Ltd) |
| 2220 | Penmix 20/80 Pen (Novo Nordisk Ltd) |
| 37617 | Rosiglitazone 2mg tablet |
| 45831 | Dacadis MR 30mg tablets (Generics (UK) Ltd) |
| 39006 | Humalog Mix25 KwikPen 100units/ml suspension for injection 3ml pre-filled pen (Eli Lilly and Company Ltd) |
| 2455 | Mixtard 20 NovoLet 100units/ml suspension for injection (Novo Nordisk Ltd) |
| 1253 | Chlorpropamide 100mg tablets |
| 39988 | Metformin 500mg oral powder sachets sugar free |
| 10229 | Humulin I Pen 100units/ml suspension for injection 3ml pre-filled pen (Eli Lilly and Company Ltd) |
| 29837 | Insulin biphasic isophane human prb 25:75; 100 units/ml Injection |
| 44304 | Glyconon 500mg Tablet (DDSA Pharmaceuticals Ltd) |
| 11990 | Metformin 500mg/5ml oral solution sugar free |
| 4790 | Mixtard 50 penfill 100 100iu/ml Penfill (Novo Nordisk Ltd) |
| 10208 | Insulatard InnoLet 100units/ml suspension for injection 3ml pre-filled pen (Novo Nordisk Ltd) |
| 19472 | Actos 45mg tablets (Takeda UK Ltd) |
| 19878 | Insulin isophane biphasic human 30/70 100units/ml suspension for injection 3ml pre-filled disposable devices |

Glucocorticoids – product codes

| prodcode | productname |
| --- | --- |
| 44 | Prednisolone 5mg gastro-resistant tablets |
| 95 | Prednisolone 5mg tablets |
| 186 | Dexamethasone 500micrograms/5ml oral solution |
| 229 | Cortisone 25mg tablets |
| 557 | Prednisolone 2.5mg gastro-resistant tablets |
| 578 | Prednisolone 1mg tablets |
| 955 | Prednisolone 5mg soluble tablets |
| 1063 | Prednesol 5mg Tablet (Sovereign Medical Ltd) |
| 1280 | Dexamethasone 2mg tablets |
| 1380 | Entocort CR 3mg capsules (AstraZeneca UK Ltd) |
| 1971 | Betnesol 500microgram soluble tablets (Focus Pharmaceuticals Ltd) |
| 2130 | Methylprednisolone 4mg tablets |
| 2368 | Prednisolone 2.5mg tablet |
| 2704 | Prednisolone 25mg tablets |
| 2949 | Prednisone 5mg tablets |
| 3345 | Sintisone Tablet (Pharmacia Ltd) |
| 3418 | Hydrocortisone 10mg tablets |
| 3557 | Prednisone 1mg tablets |
| 3898 | Budesonide 3mg gastro-resistant modified-release capsules |
| 3992 | Deflazacort 6mg tablets |
| 4535 | Hydrocortisone 20mg tablets |
| 4779 | Dexamethasone 500microgram tablets |
| 4943 | Dexamethasone 2mg/5ml oral solution sugar free |
| 5157 | Dexamethasone 2mg/5ml oral solution |
| 5490 | Deltacortril 5mg gastro-resistant tablets (Alliance Pharmaceuticals Ltd) |
| 5913 | Deltacortril 2.5mg gastro-resistant tablets (Alliance Pharmaceuticals Ltd) |
| 6095 | Budesonide 3mg gastro-resistant capsules |
| 6098 | Hydrocortone 10mg tablets (Auden McKenzie (Pharma Division) Ltd) |
| 6339 | Hydrocortisone 2.5mg muco-adhesive buccal tablets sugar free |
| 7286 | Betamethasone 500microgram soluble tablets sugar free |
| 7548 | Cortisone 5mg capsules |
| 8261 | Medrone 16mg tablets (Pfizer Ltd) |
| 9375 | Deflazacort 1mg tablets |
| 9727 | Prednisolone 50mg tablets |
| 9994 | Decadron 500microgram tablets (Merck Sharp & Dohme Ltd) |
| 10552 | Methylprednisolone 16mg tablets |
| 10574 | Cortisone acetate 5mg tablets |
| 10683 | Medrone 2mg tablets (Pfizer Ltd) |
| 10684 | Methylprednisolone 2mg tablets |
| 10754 | Hydrocortistab 20mg Tablet (Waymade Healthcare Plc) |
| 10864 | Betamethasone 500microgram tablets |
| 11149 | Betnelan 500microgram tablets (Focus Pharmaceuticals Ltd) |
| 12398 | Cortelan 25mg Tablet (Glaxo Laboratories Ltd) |
| 12400 | Cortisyl 25mg Tablet (Aventis Pharma) |
| 13043 | Hydrocortone 20mg tablets (Auden McKenzie (Pharma Division) Ltd) |
| 14076 | Hydrocortisone 5mg/5ml Oral solution |
| 14172 | Methylprednisolone 100mg tablets |
| 15555 | Medrone 4mg tablets (Pfizer Ltd) |
| 15617 | Ledercort 4mg Tablet (Wyeth Pharmaceuticals) |
| 16525 | Budenofalk 3mg gastro-resistant capsules (Dr. Falk Pharma UK Ltd) |
| 17410 | Deflazacort 30mg tablets |
| 18042 | Medrone 100mg tablets (Pfizer Ltd) |
| 18637 | Cortistab 25mg Tablet (Waymade Healthcare Plc) |
| 19141 | Prednisolone 5mg soluble tablets (AMCo) |
| 19908 | Triamcinolone 2mg Tablet |
| 20095 | Precortisyl forte 25mg Tablet (Aventis Pharma) |
| 20577 | Calcort 6mg Tablet (Shire Pharmaceuticals Ltd) |
| 21218 | Dexsol 2mg/5ml oral solution (Rosemont Pharmaceuticals Ltd) |
| 21417 | Prednisolone 5mg tablets (A A H Pharmaceuticals Ltd) |
| 21833 | Decortisyl 5mg Tablet (Roussel Laboratories Ltd) |
| 21903 | Oradexon-organon 2mg Tablet (Organon Laboratories Ltd) |
| 22555 | Calcort 1mg tablets (Shire Pharmaceuticals Ltd) |
| 23111 | Triamcinolone 4mg Tablet |
| 23210 | Cortistab 5mg Tablet (Waymade Healthcare Plc) |
| 23512 | Precortisyl 5mg Tablet (Hoechst Marion Roussel) |
| 24014 | Ledercort 2mg Tablet (Wyeth Pharmaceuticals) |
| 25272 | Precortisyl 1mg Tablet (Hoechst Marion Roussel) |
| 27962 | Deltastab 1mg Tablet (Waymade Healthcare Plc) |
| 28375 | Prednisolone 2.5mg gastro-resistant tablets (A A H Pharmaceuticals Ltd) |
| 28376 | Prednisolone 2.5mg Gastro-resistant tablet (Biorex Laboratories Ltd) |
| 28859 | Deltastab 5mg Tablet (Waymade Healthcare Plc) |
| 29112 | Calcort 30mg tablets (Shire Pharmaceuticals Ltd) |
| 29333 | Prednisolone 5mg tablets (Actavis UK Ltd) |
| 31327 | Prednisolone steaglate 6.65mg tablet |
| 31532 | Prednisolone 5mg gastro-resistant tablets (A A H Pharmaceuticals Ltd) |
| 32803 | Prednisolone 5mg gastro-resistant tablets (Actavis UK Ltd) |
| 32835 | Prednisolone 5mg tablets (Wockhardt UK Ltd) |
| 33691 | Prednisolone 5mg Gastro-resistant tablet (Biorex Laboratories Ltd) |
| 33988 | Prednisolone 5mg Tablet (Co-Pharma Ltd) |
| 33990 | Prednisolone 5mg Tablet (IVAX Pharmaceuticals UK Ltd) |
| 34109 | Prednisolone 5 mg gastro-resistant tablet |
| 34393 | Prednisolone 5mg gastro-resistant tablets (Teva UK Ltd) |
| 34404 | Prednisolone 1mg tablets (Actavis UK Ltd) |
| 34452 | Prednisolone 1mg tablets (A A H Pharmaceuticals Ltd) |
| 34461 | Prednisolone 2.5mg gastro-resistant tablets (Actavis UK Ltd) |
| 34631 | Prednisolone 1mg Tablet (Co-Pharma Ltd) |
| 34660 | Prednisolone 1mg tablets (Kent Pharmaceuticals Ltd) |
| 34748 | Prednisolone 1mg tablets (Teva UK Ltd) |
| 34781 | Prednisolone 5mg tablets (Kent Pharmaceuticals Ltd) |
| 34801 | Dexamethasone 0.5mg/5ml Oral solution (Rosemont Pharmaceuticals Ltd) |
| 34880 | Dexamethasone 2mg tablets (Aspen Pharma Trading Ltd) |
| 34914 | Prednisolone 1mg Tablet (Celltech Pharma Europe Ltd) |
| 34915 | Dexamethasone 500microgram tablets (Organon Laboratories Ltd) |
| 34978 | Prednisolone 1mg tablets (Wockhardt UK Ltd) |
| 36055 | Dexamethasone 2mg Tablet (Hillcross Pharmaceuticals Ltd) |
| 37203 | Beclometasone 5mg gastro-resistant modified-release tablets |
| 38022 | Hydrocortisone 10mg/5ml oral suspension |
| 38054 | Hydrocortisone Tablet |
| 38407 | Prednisolone 20mg tablet |
| 39067 | Clipper 5mg gastro-resistant modified-release tablets (Chiesi Ltd) |
| 41335 | Calcort 6mg tablets (Sanofi) |
| 41515 | Prednisolone 5mg tablets (Teva UK Ltd) |
| 41745 | Prednisolone 25mg tablets (Zentiva) |
| 43544 | Prednisone 5mg Tablet (Knoll Ltd) |
| 44380 | Prednisone 1mg modified-release tablets |
| 44723 | Prednisone 5mg modified-release tablets |
| 44802 | Lodotra 5mg modified-release tablets (Napp Pharmaceuticals Ltd) |
| 44803 | Lodotra 2mg modified-release tablets (Napp Pharmaceuticals Ltd) |
| 45234 | Dexamethasone 100microgram capsules |
| 45302 | Prednisolone 5mg Tablet (Biorex Laboratories Ltd) |
| 46280 | Hydrocortisone 2.5mg muco-adhesive buccal tablets sugar free (Auden McKenzie (Pharma Division) Ltd) |
| 46711 | Prednisone 2mg modified-release tablets |
| 47142 | Prednisolone 5mg Soluble tablet (Amdipharm Plc) |
| 47225 | Budesonide 9mg gastro-resistant granules sachets |
| 48088 | Budenofalk 9mg gastro-resistant granules sachets (Dr. Falk Pharma UK Ltd) |
| 50225 | Betnesol 500microgram soluble tablets (Waymade Healthcare Plc) |
| 51722 | Hydrocortisone 5mg/5ml oral suspension |
| 51753 | Prednisolone 1mg tablets (Co-Pharma Ltd) |
| 51824 | Hydrocortisone 5mg/5ml oral suspension sugar free |
| 51849 | Hydrocortisone 1mg/5ml oral suspension |
| 51871 | Hydrocortisone 2mg capsules |
| 51872 | Hydrocortisone 2.5mg capsules |
| 51997 | Budesonide 9mg gastro-resistant granules sachets |
| 52053 | Hydrocortisone 3mg/5ml oral suspension |
| 52396 | Dexamethasone 1mg/5ml oral solution |
| 53143 | Cortisone 25mg tablets (A A H Pharmaceuticals Ltd) |
| 53207 | Dexamethasone tablets |
| 53313 | Prednisolone 20mg/5ml oral suspension |
| 53336 | Prednisolone 25mg tablets (A A H Pharmaceuticals Ltd) |
| 53705 | Cortisone acetate 5mg Capsule (Martindale Pharmaceuticals Ltd) |
| 53953 | Hydrocortisone 5mg modified-release tablets |
| 54118 | Prednisolone 25mg/5ml oral suspension |
| 54432 | Lodotra 1mg modified-release tablets (Napp Pharmaceuticals Ltd) |
| 54434 | Prednisolone 2.5mg/5ml oral suspension |
| 54793 | Dexamethasone 2mg/5ml oral suspension |
| 54794 | Hydrocortisone 20mg modified-release tablets |
| 55024 | Prednisolone 5mg/5ml oral solution |
| 55401 | Dexamethasone 500microgram tablets (A A H Pharmaceuticals Ltd) |
| 55480 | Prednisolone 2.5mg gastro-resistant tablets (Alliance Pharmaceuticals Ltd) |
| 56144 | Budenofalk 9mg gastro-resistant granules sachets (Dr. Falk Pharma UK Ltd) |
| 56319 | Hydrocortisone 2.5mg muco-adhesive buccal tablets sugar free (A A H Pharmaceuticals Ltd) |
| 56347 | Dexamethasone 5mg/5ml oral solution |
| 56443 | Dexamethasone 10mg/5ml oral solution |
| 56891 | Prednisolone 1mg tablets (Waymade Healthcare Plc) |
| 57931 | Hydrocortisone 20mg tablets (Teva UK Ltd) |
| 58000 | Prednisolone 5mg tablets (Almus Pharmaceuticals Ltd) |
| 58061 | Prednisone 50mg tablets |
| 58234 | Prednisolone 10mg/5ml oral solution |
| 58369 | Prednisolone 5mg tablets (Boston Healthcare Ltd) |
| 58384 | Prednisolone 1mg tablets (Almus Pharmaceuticals Ltd) |
| 58474 | Dexamethasone 2mg/5ml oral solution sugar free (A A H Pharmaceuticals Ltd) |
| 58592 | Plenadren 20mg modified-release tablets (ViroPharma Ltd) |
| 58987 | Prednisolone 5mg gastro-resistant tablets (Phoenix Healthcare Distribution Ltd) |
| 59229 | Dilacort 5mg gastro-resistant tablets (Auden McKenzie (Pharma Division) Ltd) |
| 59283 | Dilacort 2.5mg gastro-resistant tablets (Auden McKenzie (Pharma Division) Ltd) |
| 59338 | Prednisolone 1mg/5ml oral solution |
| 59418 | Plenadren 5mg modified-release tablets (ViroPharma Ltd) |
| 59912 | Prednisolone 5mg gastro-resistant tablets (Waymade Healthcare Plc) |
| 60064 | Dexamethasone 10mg/5ml oral solution sugar free |
| 60120 | Dexamethasone 2mg tablets (Alliance Healthcare (Distribution) Ltd) |
| 60421 | Prednisolone 5mg tablets (Co-Pharma Ltd) |
| 60946 | Entocort CR 3mg capsules (Waymade Healthcare Plc) |
| 61132 | Prednisolone 1mg tablets (Boston Healthcare Ltd) |
| 61162 | Prednisolone 5mg tablets (Waymade Healthcare Plc) |
| 61689 | Prednisolone 5mg soluble tablets (A A H Pharmaceuticals Ltd) |
| 61791 | Hydrocortisone 2.5mg muco-adhesive buccal tablets sugar free (Waymade Healthcare Plc) |
| 62656 | Prednisone 5mg Tablet (Hillcross Pharmaceuticals Ltd) |
| 62909 | Dexamethasone 2mg tablets (A A H Pharmaceuticals Ltd) |
| 63066 | Prednisolone 2.5mg tablets |
| 63082 | Prednisolone 20mg tablets |
| 63138 | Hydrocortisone 5mg/5ml oral solution |
| 63172 | Prednisolone 10mg tablets |
| 63214 | Prednisolone 5mg soluble tablets (Alliance Healthcare (Distribution) Ltd) |
| 63549 | Prednisolone 1mg/ml oral solution (Logixx Pharma Solutions Ltd) |
| 63791 | Prednisolone 5mg/5ml oral solution unit dose |
| 63893 | Budesonide 9mg modified-release tablets |
| 64007 | Pevanti 10mg tablets (AMCo) |
| 64008 | Pevanti 2.5mg tablets (AMCo) |
| 64009 | Pevanti 20mg tablets (AMCo) |
| 64050 | Martapan 2mg/5ml oral solution (Martindale Pharmaceuticals Ltd) |
| 64059 | Hydrocortisone 2.5mg/5ml oral suspension |
| 64128 | Pevanti 5mg tablets (AMCo) |
| 64221 | Prednisolone 5mg/5ml oral suspension |
| 64235 | Betamethasone 500microgram soluble tablets sugar free (Alliance Healthcare (Distribution) Ltd) |
| 64416 | Prednisolone 10mg/ml oral solution sugar free |
| 64557 | Cortiment 9mg modified-release tablets (Ferring Pharmaceuticals Ltd) |
| 64747 | Dexamethasone 2mg/5ml oral solution |
| 64766 | Dexamethasone 20mg/5ml oral solution sugar free |
| 64787 | Hydrocortisone 10mg tablets (Almus Pharmaceuticals Ltd) |

NSAIDs – product codes

| prodcode | prodname |
| --- | --- |
| 33669 | Diclofenac 50mg Gastro-resistant tablet (Genus Pharmaceuticals Ltd) |
| 32601 | Econac 100mg suppositories (Mercury Pharma Group Ltd) |
| 3216 | Indometacin 25mg modified-release tablets |
| 33801 | Opustan 250mg Capsule (Opus Pharmaceuticals Ltd) |
| 3182 | Froben 50mg tablets (Abbott Laboratories Ltd) |
| 14678 | Defanac sr 100mg Modified-release tablet (Ranbaxy (UK) Ltd) |
| 50602 | Diclofenac potassium 50mg tablets (Alliance Healthcare (Distribution) Ltd) |
| 29037 | Valdic 100 Retard tablets (Fannin UK Ltd) |
| 36543 | Aspirin 100mg effervescent tablets |
| 2387 | Arthrotec 75 gastro-resistant tablets (Pfizer Ltd) |
| 50266 | Ibuprofen 200mg caplets (The Boots Company Plc) |
| 30164 | Lemsip Cold and Flu Sinus 12 Hr Ibuprofen + Pseudoephedrine modified-release capsules (Reckitt Benckiser Healthcare (UK) Ltd) |
| 13606 | Flexin-25 Continus tablets (Napp Pharmaceuticals Ltd) |
| 46967 | Mefenamic acid 250mg Capsule (Sandoz Ltd) |
| 17532 | Dicloflex Retard 100mg tablets (Kent Pharmaceuticals Ltd) |
| 7490 | Froben 100mg suppositories (Abbott Laboratories Ltd) |
| 35653 | Etopan XL 600mg tablets (Taro Pharmaceuticals (UK) Ltd) |
| 46848 | Naproxen 500mg Gastro-resistant tablet (Almus Pharmaceuticals Ltd) |
| 53700 | Naproxen 250mg gastro-resistant tablets (Alliance Healthcare (Distribution) Ltd) |
| 3326 | Oruvail 100mg Modified-release capsule (Hawgreen Ltd) |
| 35935 | Meloxicam 7.5mg tablets (Somex Pharma) |
| 15767 | Ibuprofen 5% foam |
| 57006 | Diclofenac sodium 25mg gastro-resistant tablets (Phoenix Healthcare Distribution Ltd) |
| 51293 | Diclofenac potassium 50mg tablets (Phoenix Healthcare Distribution Ltd) |
| 42108 | Ibuprofen 200mg tablets (OBG Pharmaceuticals Ltd) |
| 387 | Surgam 200mg tablets (Sanofi) |
| 18527 | Mandafen 400mg tablets (M & A Pharmachem Ltd) |
| 2386 | Voltarol Retard 100mg tablets (Novartis Pharmaceuticals UK Ltd) |
| 12766 | Flurbiprofen 8.75mg lozenges |
| 612 | Dicloflex 25mg gastro-resistant tablets (Dexcel-Pharma Ltd) |
| 48059 | Diclofenac potassium 50mg tablets (A A H Pharmaceuticals Ltd) |
| 736 | Indometacin 50mg capsules |
| 50058 | Voltarol 50mg dispersible tablets (Doncaster Pharmaceuticals Ltd) |
| 52905 | Aspirin 300mg tablets (Lloyds Pharmacy Ltd) |
| 51306 | Parecoxib 40mg powder for solution for injection vials |
| 29759 | Aspro Tablet (Roche Consumer Health) |
| 20016 | Tolmetin 400mg Capsule |
| 18261 | Aspirin 500mg with Papaveretum 7.71mg dispersible tablets |
| 34527 | Ibuprofen 200mg tablets (Zentiva) |
| 44483 | Nurofen Express 512mg tablets (Reckitt Benckiser Healthcare (UK) Ltd) |
| 53622 | Aspirin 300mg Tablet (M & A Pharmachem Ltd) |
| 8672 | Feldene 20mg suppositories (Pfizer Ltd) |
| 31429 | Timpron 250mg Gastro-resistant tablet (Berk Pharmaceuticals Ltd) |
| 28816 | Rheuflex 500mg Tablet (Goldshield Pharmaceuticals Ltd) |
| 34290 | Naproxen 250mg gastro-resistant tablets (Teva UK Ltd) |
| 33935 | Nurofen Maximum Strength Migraine Pain 684mg caplets (Reckitt Benckiser Healthcare (UK) Ltd) |
| 4254 | Cytotec 200microgram tablets (Pfizer Ltd) |
| 27055 | Diclofenac sodium 50mg gastro-resistant tablets (Kent Pharmaceuticals Ltd) |
| 36577 | Indometacin 50mg Capsule (Meridian Healthcare (UK) Ltd) |
| 21815 | Arthrofen 600 tablets (Ashbourne Pharmaceuticals Ltd) |
| 5175 | Celebrex 100mg capsules (Pfizer Ltd) |
| 30327 | Jomethid XL 200mg capsules (Actavis UK Ltd) |
| 33568 | Ketoprofen 200mg Modified-release capsule (Actavis UK Ltd) |
| 18151 | Voltarol Pain-eze 1% Emulgel (Novartis Consumer Health UK Ltd) |
| 5085 | Voltarol Rapid 50mg tablets (Novartis Pharmaceuticals UK Ltd) |
| 360 | Brufen 100mg/5ml syrup (Abbott Laboratories Ltd) |
| 34434 | Aspirin 75mg dispersible tablets (Thornton & Ross Ltd) |
| 55009 | Brufen 600mg effervescent granules sachets (Necessity Supplies Ltd) |
| 7481 | Lederfen 450mg Tablet (Wyeth Pharmaceuticals) |
| 24111 | Ketorolac 10mg/1ml solution for injection ampoules |
| 49132 | Voltarol 1% Emulgel (Necessity Supplies Ltd) |
| 393 | Disprin 300mg dispersible tablets (Reckitt Benckiser Healthcare (UK) Ltd) |
| 53804 | Aspirin 300mg gastro-resistant tablets (Alliance Healthcare (Distribution) Ltd) |
| 13882 | Imazin XL tablets (Napp Pharmaceuticals Ltd) |
| 42793 | Diclofenac 100mg Modified-release tablet (IVAX Pharmaceuticals UK Ltd) |
| 45988 | Ibuprofen 200mg / Phenylephrine 5mg tablets |
| 43434 | Aspirin 300mg gastro-resistant tablets (A A H Pharmaceuticals Ltd) |
| 349 | VOLTAROL 75 MG INJ |
| 2366 | Flurbiprofen 100mg tablets |
| 34595 | Mefenamic acid 500mg tablets (Zentiva) |
| 48165 | Aspirin 300mg tablets (Aspar Pharmaceuticals Ltd) |
| 42604 | Mobiflex 20mg tablets (Meda Pharmaceuticals Ltd) |
| 28172 | Ibuprofen 300mg / Pseudoephedrine 45mg modified-release capsules |
| 40381 | Aspirin 75mg Soluble tablet (C P Pharmaceuticals Ltd) |
| 526 | Aceclofenac 100mg tablets |
| 11522 | Pennsaid 1.50% Cutaneous solution (Provalis Healthcare Ltd) |
| 51614 | Ibuprofen 200mg caplets (Lloyds Pharmacy Ltd) |
| 16286 | Lofensaid Retard 75 tablets (Opus Pharmaceuticals Ltd) |
| 11554 | Ibuprofen 200mg / Codeine 12.8mg tablets |
| 1866 | Naprosyn 500mg tablets (Roche Products Ltd) |
| 25433 | Radian B Muscle lotion (Thornton & Ross Ltd) |
| 31469 | Apsifen -f 600mg Tablet (Approved Prescription Services Ltd) |
| 17818 | Ketovail 100mg modified-release capsules (Teva UK Ltd) |
| 40141 | Ketoprofen 100mg capsules (A A H Pharmaceuticals Ltd) |
| 57007 | Mefenamic acid 250mg capsules (Essential Generics Ltd) |
| 10785 | Fenbid 300mg Spansules (Mercury Pharma Group Ltd) |
| 26575 | Streflam 8.75mg Lozenge (Crookes Healthcare Ltd) |
| 57297 | Mefenamic acid 500mg tablets (Alliance Healthcare (Distribution) Ltd) |
| 26083 | Indolar 100mg Suppository (Lagap) |
| 34536 | Ibuprofen 400mg tablets (IVAX Pharmaceuticals UK Ltd) |
| 8882 | Feldene 0.50% Sports gel (Pfizer Ltd) |
| 14084 | Diclovol 75mg SR tablets (Arun Pharmaceuticals Ltd) |
| 3496 | Nycopren 250mg gastro-resistant tablets (Ardern Healthcare Ltd) |
| 56441 | Calprofen 100mg/5ml oral suspension 5ml sachets (McNeil Products Ltd) |
| 50080 | Dynastat 40mg powder and solvent for solution for injection vials (Pfizer Ltd) |
| 52856 | Co-codaprin 8mg/400mg tablets |
| 21821 | Lidifen f 600mg Tablet (Berk Pharmaceuticals Ltd) |
| 377 | Aspirin 300mg dispersible tablets |
| 54284 | Aspirin 75mg dispersible tablets (Almus Pharmaceuticals Ltd) |
| 54137 | Ibuprofen 400mg tablets (Aspar Pharmaceuticals Ltd) |
| 5254 | Celecoxib 200mg capsules |
| 32105 | Mefenamic acid 500mg tablets (A A H Pharmaceuticals Ltd) |
| 34898 | Mefenamic acid 250mg Capsule (Berk Pharmaceuticals Ltd) |
| 43032 | Inoven 200mg Tablet (Janssen-Cilag Ltd) |
| 54660 | Diclofenac sodium 50mg capsules |
| 13459 | Dysman 500 tablets (Ashbourne Pharmaceuticals Ltd) |
| 14333 | Ibuprofen 400mg capsules |
| 43426 | Ibuprofen 5% gel (A A H Pharmaceuticals Ltd) |
| 10310 | Aspirin powder |
| 21045 | Ibumetin 400mg Tablet (Alfred Benzon (UK) Ltd) |
| 13347 | Alrheumat 50mg Capsule (Bayer Plc) |
| 37541 | Aspirin 227mg medicated chewing-gum |
| 254 | Aspirin 300mg tablets |
| 928 | Diclofenac sodium 25mg tablets |
| 18820 | Fenpaed 100mg/5ml Oral suspension (Pinewood Healthcare) |
| 31954 | Aspirin 75mg dispersible tablets (Teva UK Ltd) |
| 53576 | Arcoxia 120mg tablets (Doncaster Pharmaceuticals Ltd) |
| 36260 | Mendys 250mg Capsule (Kent Pharmaceuticals Ltd) |
| 45851 | Aspirin 300mg Soluble tablet (Ranbaxy (UK) Ltd) |
| 41365 | Axorid 200mg/20mg modified-release capsules (Meda Pharmaceuticals Ltd) |
| 4965 | Piroxicam 20mg orodispersible tablets sugar free |
| 34961 | Ibuprofen 600mg tablets (Sandoz Ltd) |
| 50166 | Generic Anadin Extra tablets |
| 43541 | Piroxicam 10mg capsules (Actavis UK Ltd) |
| 20709 | MEFENAMIC ACID DISPERSIBLE |
| 26231 | Timpron 500mg Gastro-resistant tablet (Berk Pharmaceuticals Ltd) |
| 27362 | Diclofenac 100mg Modified-release tablet (Actavis UK Ltd) |
| 13380 | Clinoril 200mg tablets (Merck Sharp & Dohme Ltd) |
| 55582 | Celebrex 200mg capsules (Lexon (UK) Ltd) |
| 12964 | Aspirin 600mg / Caffeine 50mg oral powder sachets sugar free |
| 14385 | Cuprofen 200mg Tablet (SSL International Plc) |
| 18798 | Lofensaid 50mg gastro-resistant tablets (Opus Pharmaceuticals Ltd) |
| 9822 | Arcoxia 120mg tablets (Merck Sharp & Dohme Ltd) |
| 41623 | Piroxicam 20mg capsules (IVAX Pharmaceuticals UK Ltd) |
| 9886 | Dicloflex 50mg Gastro-resistant tablet (Ratiopharm UK Ltd) |
| 3043 | Ketoprofen 200mg modified-release capsules |
| 21843 | Pranoxen continus 375mg Tablet (Napp Pharmaceuticals Ltd) |
| 17491 | Dicloflex sr 75mg Tablet (Ratiopharm UK Ltd) |
| 10898 | Voltarol Ophtha 0.1% eye drops 0.3ml unit dose (Spectrum Thea Pharmaceuticals Ltd) |
| 48562 | Ibuprofen 100mg/5ml oral suspension 5ml sachets sugar free |
| 49788 | Voltarol 1% Emulgel (Doncaster Pharmaceuticals Ltd) |
| 25283 | Valenac ec 50mg Gastro-resistant tablet (Shire Pharmaceuticals Ltd) |
| 36650 | Nurofen 200mg tablets (Reckitt Benckiser Healthcare (UK) Ltd) |
| 30806 | Rhumalgan 50mg Tablet (Lagap) |
| 36787 | Nurofen Express 684mg caplets (Reckitt Benckiser Healthcare (UK) Ltd) |
| 1156 | Ibugel 5% gel (Dermal Laboratories Ltd) |
| 10978 | Voltarol 25mg Suppository (Novartis Pharmaceuticals UK Ltd) |
| 56762 | Naproxen 100mg/5ml oral suspension |
| 25718 | Angettes 75 tablets (Bristol-Myers Squibb Pharmaceuticals Ltd) |
| 5200 | Voltarol 50mg suppositories (Novartis Pharmaceuticals UK Ltd) |
| 33321 | Indometacin 50mg capsules (Actavis UK Ltd) |
| 10169 | Brexidol 20mg tablets (Chiesi Ltd) |
| 3972 | Naprosyn EC 250mg tablets (Roche Products Ltd) |
| 18640 | Tolectin 200mg Capsule (Cilag Pharmaceuticals Ltd) |
| 29704 | Paxofen 200mg Tablet (M A Steinhard Ltd) |
| 920 | Indocid 100mg suppositories (Merck Sharp & Dohme Ltd) |
| 40484 | Orudis 100mg capsules (Sanofi) |
| 162 | Arthrotec 50 gastro-resistant tablets (Pfizer Ltd) |
| 10305 | Aspirin 162.5mg capsules |
| 49685 | Aspirin 75mg dispersible tablets (Sigma Pharmaceuticals Plc) |
| 31001 | Cullens headache powders Sachets (Cullen and Davidson) |
| 41364 | Ketoprofen 100mg / Omeprazole 20mg modified-release capsules |
| 1086 | Ibuprofen 600mg tablets |
| 1496 | Indocid R 75mg capsules (Merck Sharp & Dohme Ltd) |
| 29465 | Piroxicam 20mg capsules (Actavis UK Ltd) |
| 46920 | Ketoprofen 200mg Modified-release capsule (Generics (UK) Ltd) |
| 661 | Naproxen 250mg tablets |
| 32036 | Aspirin 75mg dispersible tablets (Actavis UK Ltd) |
| 12122 | Orudis 50mg Capsule (Hawgreen Ltd) |
| 14994 | Clotam Rapid 200mg tablets (Galen Ltd) |
| 22305 | Disprin Extra dispersible tablets (Reckitt Benckiser Healthcare (UK) Ltd) |
| 34386 | Aspirin 300mg tablets (Actavis UK Ltd) |
| 7840 | Oruvail 150mg Modified-release capsule (Hawgreen Ltd) |
| 23204 | Pardelprin MR 75mg capsules (Actavis UK Ltd) |
| 38511 | Feminax Ultra 250mg gastro-resistant tablets (Bayer Plc) |
| 24193 | Imbrilon 25mg Capsule (Berk Pharmaceuticals Ltd) |
| 37648 | Nurofen Express 400mg liquid capsules (Reckitt Benckiser Healthcare (UK) Ltd) |
| 4506 | Volsaid Retard 75 tablets (Chiesi Ltd) |
| 48568 | Boots Rapid Ibuprofen lysine 342mg tablets (The Boots Company Plc) |
| 6115 | Diclofenac sodium 3% gel |
| 8544 | Fenbufen 450mg tablets |
| 7913 | Tiaprofenic acid 200mg tablets |
| 43456 | Anadin LiquiFast 400mg capsules (Pfizer Consumer Healthcare Ltd) |
| 1902 | Aspirn 600mg gastro-resistant tablets |
| 34744 | Diclofenac 100mg Modified-release capsule (Sandoz Ltd) |
| 40215 | Oruvail 100 modified-release capsules (Sanofi) |
| 31945 | Naproxen 500mg Gastro-resistant tablet (Sterwin Medicines) |
| 31944 | Diclofenac sodium 25mg gastro-resistant tablets (Generics (UK) Ltd) |
| 1049 | Nu-seals aspirin 600mg Tablet (Eli Lilly and Company Ltd) |
| 16001 | Ibuprofen 200mg tablets (A A H Pharmaceuticals Ltd) |
| 21770 | Paracetamol 200mg with aspirin 300mg dispersible tablet |
| 37816 | Cuprofen PLUS tablets (SSL International Plc) |
| 55434 | Ibuprofen 400mg tablets (Bristol Laboratories Ltd) |
| 56898 | Rhumalgan SR 75mg capsules (Actavis UK Ltd) |
| 2606 | Ketoprofen 2.5% gel |
| 23932 | Aspro Clear 300mg effervescent tablets (Bayer Plc) |
| 52044 | Aspirin 300mg caplets (The Boots Company Plc) |
| 40756 | Dicloflex 25mg gastro-resistant tablets (Almus Pharmaceuticals Ltd) |
| 259 | Mefenamic acid 250mg capsules |
| 26967 | Alka-Seltzer XS effervescent tablets (Bayer Plc) |
| 51827 | Mefenamic acid 500mg tablets (Sigma Pharmaceuticals Plc) |
| 24617 | Tiloket 2.5% gel (Tillomed Laboratories Ltd) |
| 47501 | Rhumalgan SR 75mg capsules (Almus Pharmaceuticals Ltd) |
| 9044 | Codis 500 dispersible tablets (Reckitt Benckiser Healthcare (UK) Ltd) |
| 58048 | Diclofenac sodium 50mg gastro-resistant tablets (Waymade Healthcare Plc) |
| 474 | Celecoxib 100mg capsules |
| 1755 | Piroxicam 20mg capsules |
| 8969 | Lodine 300mg Capsule (Shire Pharmaceuticals Ltd) |
| 5407 | Naproxen 125mg/5ml oral suspension |
| 13083 | Deep Relief gel (The Mentholatum Company Ltd) |
| 35749 | Radian B Ibuprofen Massage stick (Thornton & Ross Ltd) |
| 53791 | Aspirin 150mg suppositories (Alliance Healthcare (Distribution) Ltd) |
| 18329 | Enprin 75mg gastro-resistant tablets (Galpharm International Ltd) |
| 48218 | Dicloflex sr 100mg Tablet (Teva UK Ltd) |
| 29068 | Nurofen Extra Strength 400mg capsules (Reckitt Benckiser Healthcare (UK) Ltd) |
| 4216 | Brufen 600mg tablets (Abbott Laboratories Ltd) |
| 26216 | Timpron 500mg Tablet (Berk Pharmaceuticals Ltd) |
| 55153 | Nurofen Express Soluble 400mg oral powder sachets (Reckitt Benckiser Healthcare (UK) Ltd) |
| 26970 | Ibuprofen 100mg/5ml oral suspension sugar free (Teva UK Ltd) |
| 2293 | Voltarol 25mg/ml Injection (Novartis Pharmaceuticals UK Ltd) |
| 51237 | Voltarol 1% Emulgel (Waymade Healthcare Plc) |
| 52617 | Ibuprofen 100mg/5ml oral suspension sugar free (Sigma Pharmaceuticals Plc) |
| 1051 | Indometacin 75mg modified-release tablets |
| 34743 | Naproxen 500mg gastro-resistant tablets (A A H Pharmaceuticals Ltd) |
| 33645 | Diclofenac 75mg Modified-release tablet (IVAX Pharmaceuticals UK Ltd) |
| 1984 | Diclofenac sodium 100mg modified-release tablets |
| 31383 | Dexomon 75mg SR tablets (Hillcross Pharmaceuticals Ltd) |
| 3421 | Diclomax sr 75mg Modified-release capsule (Provalis Healthcare Ltd) |
| 44313 | Indoflex 25mg Capsule (Unimed Pharmaceuticals Ltd) |
| 4679 | Asasantin Retard capsules (Boehringer Ingelheim Ltd) |
| 57112 | Ibuprofen 400mg tablets (Alliance Healthcare (Distribution) Ltd) |
| 39823 | Dicloflex 50mg gastro-resistant tablets (Almus Pharmaceuticals Ltd) |
| 33559 | Diclofenac 50mg Tablet (C P Pharmaceuticals Ltd) |
| 29352 | Ibuprofen 100mg/5ml oral suspension sugar free (Vantage) |
| 41367 | Ketoprofen 200mg / Omeprazole 20mg modified-release capsules |
| 34362 | Diclofenac 25mg Gastro-resistant tablet (Genus Pharmaceuticals Ltd) |
| 18662 | Indomod 75mg modified-release capsules (Pfizer Ltd) |
| 4911 | Ibuprofen 400mg Granules |
| 45840 | Aspirin 300mg Dispersible tablet (Numark Management Ltd) |
| 3710 | Piroxicam 20mg dispersible tablets |
| 12075 | Mobiflex 20mg Tablet (Roche Products Ltd) |
| 849 | Ibumed 400mg Tablet (Medipharma Ltd) |
| 15363 | Nurofen Cold and Flu tablets (Reckitt Benckiser Healthcare (UK) Ltd) |
| 10149 | Ibuprofen 200mg capsules |
| 27778 | Solpaflex 2.50% Gel (GlaxoSmithKline Consumer Healthcare) |
| 34385 | Aspirin 75mg Soluble tablet (Co-operative) |
| 50343 | Feldene 0.5% gel (Doncaster Pharmaceuticals Ltd) |
| 33180 | Ketoprofen cr 200mg Capsule (Bristol-Myers Squibb Pharmaceuticals Ltd) |
| 20384 | Flamatak MR 100mg tablets (Actavis UK Ltd) |
| 31950 | Diclofenac sodium 50mg gastro-resistant tablets (Sterwin Medicines) |
| 54783 | Naproxen 250mg tablets (Teva UK Ltd) |
| 3409 | Feldene 20mg Orodispersible tablet (Pfizer Ltd) |
| 6696 | Micropirin 75mg gastro-resistant tablets (Dexcel-Pharma Ltd) |
| 15180 | Naproxen and misoprostol 500mgwith200microgram combined Tablet |
| 31787 | Econac SR 75mg tablets (Mercury Pharma Group Ltd) |
| 46342 | Medifen 3with months 100mg/5ml Oral suspension (SSL International Plc) |
| 55486 | Naproxen 500mg tablets (Teva UK Ltd) |
| 1446 | Voltarol 50mg Tablet (Novartis Pharmaceuticals UK Ltd) |
| 3597 | Nurofen 200mg Soluble tablet (Crookes Healthcare Ltd) |
| 7539 | Beechams Powders oral powder sachets (SmithKline Beecham Plc) |
| 40185 | Oruvail 200 modified-release capsules (Sanofi) |
| 4762 | Ibuleve Sports 5% gel (Dendron Ltd) |
| 50059 | Celebrex 100mg capsules (Necessity Supplies Ltd) |
| 32992 | Aspirin 75mg gastro-resistant tablets (Generics (UK) Ltd) |
| 22283 | Lemsip flu 12 hr Modified-release capsule (Reckitt Benckiser Healthcare (UK) Ltd) |
| 55913 | Voltarol 50mg suppositories (Lexon (UK) Ltd) |
| 24025 | Caprin 300mg gastro-resistant tablets (Pinewood Healthcare) |
| 784 | Ibuprofen 300mg modified-release capsules |
| 14707 | Defanac Retard 100mg tablets (Ranbaxy (UK) Ltd) |
| 53617 | Ibuprofen and codeine 200mg+12.8mg Tablet (Almus Pharmaceuticals Ltd) |
| 37688 | Diclofenac sodium 1% gel |
| 31064 | Mobiflex 20mg Granules (Roche Products Ltd) |
| 16637 | Ketorolac 10mg tablets |
| 44730 | Mentholatum Ibuprofen 5% gel (The Mentholatum Company Ltd) |
| 31959 | Indometacin 50mg capsules (A A H Pharmaceuticals Ltd) |
| 597 | Diclofenac potassium 50mg tablets |
| 41512 | Aspirin 75mg gastro-resistant tablets (Teva UK Ltd) |
| 44112 | Voltarol Joint Pain 12.5mg tablets (Novartis Consumer Health UK Ltd) |
| 11999 | Orudis 100mg Capsule (Hawgreen Ltd) |
| 5812 | Etoricoxib 90mg tablets |
| 34 | Aspirin 75mg gastro-resistant tablets |
| 53178 | Aspirin 75mg gastro-resistant tablets (Wockhardt UK Ltd) |
| 5938 | Etoricoxib 120mg tablets |
| 18217 | Aspirin 300mg orodispersible tablets sugar free |
| 55894 | Naproxen 500mg gastro-resistant tablets (Phoenix Healthcare Distribution Ltd) |
| 52141 | Mobilan 25mg Capsule (Galen Ltd) |
| 11540 | Diclofenac 16mg/ml topical solution |
| 650 | Etoricoxib 60mg tablets |
| 54870 | Piroxicam 0.5% gel (Alliance Healthcare (Distribution) Ltd) |
| 50117 | Brufen 100mg/5ml syrup (Lexon (UK) Ltd) |
| 20907 | Sudafed Sinus Pressure & Pain tablets (McNeil Products Ltd) |
| 5401 | Voltarol Rapid 25mg tablets (Novartis Pharmaceuticals UK Ltd) |
| 47401 | Ibuprofen 5% gel (Galpharm International Ltd) |
| 29330 | Diclofenac sodium 50mg gastro-resistant tablets (Sandoz Ltd) |
| 9144 | Caprin 75mg gastro-resistant tablets (Wockhardt UK Ltd) |
| 55230 | Aspirin 300mg dispersible tablets (Kent Pharmaceuticals Ltd) |
| 48546 | Ibuprofen 400mg caplets (Bristol Laboratories Ltd) |
| 31777 | Piroxicam 20mg dispersible tablets (Generics (UK) Ltd) |
| 5080 | Celebrex 200mg capsules (Pfizer Ltd) |
| 33113 | Artracin 50mg Capsule (DDSA Pharmaceuticals Ltd) |
| 14776 | Surgam 300mg tablets (Sanofi) |
| 12607 | KETOROLAC TROMETAMOL 30 MG/ML INJ |
| 10589 | Fenopron 600 tablets (Typharm Ltd) |
| 34212 | Diclofenac 75mg Modified-release tablet (Genus Pharmaceuticals Ltd) |
| 26522 | Meflam 500mg Tablet (Trinity Pharmaceuticals Ltd) |
| 56554 | Naproxen 250mg/5ml oral suspension |
| 30923 | Diclofenac 100mg suppositories (A A H Pharmaceuticals Ltd) |
| 27782 | Ibuprofen 400mg tablets (Teva UK Ltd) |
| 2197 | Naproxen 375mg Tablet |
| 28168 | Nurofen Recovery 200mg orodispersible tablets (Reckitt Benckiser Healthcare (UK) Ltd) |
| 37805 | Polyurethane foam Film dressing 15cmx15cm |
| 50555 | Aspirin 300mg dispersible tablets (Doncaster Pharmaceuticals Ltd) |
| 28900 | Indometacin 25mg Capsule (Generics (UK) Ltd) |
| 53331 | Ibuprofen 100mg/5ml oral suspension sugar free (Alliance Healthcare (Distribution) Ltd) |
| 50317 | Voltarol 75mg SR tablets (Lexon (UK) Ltd) |
| 48161 | Naproxen 500mg Tablet (Almus Pharmaceuticals Ltd) |
| 22138 | Aspirin 324mg modified-release tablets |
| 34447 | Ibuprofen 200mg tablets (Thornton & Ross Ltd) |
| 24007 | Valrox 500mg Tablet (Shire Pharmaceuticals Ltd) |
| 6666 | Dipyridamole 200mg modified-release / Aspirin 25mg capsules |
| 41569 | Aspirin 300mg tablets (A A H Pharmaceuticals Ltd) |
| 31858 | Caspac xl 162.5mg Capsule (Pharmacia Ltd) |
| 31210 | Aspirin 300mg Tablet (Co-operative) |
| 52229 | Voltarol 1% Emulgel (Sigma Pharmaceuticals Plc) |
| 6881 | Solaraze 3% gel (Almirall Ltd) |
| 57045 | Voltarol 50mg dispersible tablets (Waymade Healthcare Plc) |
| 1739 | Brufen 400mg tablets (Abbott Laboratories Ltd) |
| 177 | Indometacin 25mg capsules |
| 1231 | Ketoprofen 100mg capsules |
| 7424 | Fenbufen 300mg capsules |
| 39019 | Brufen Retard 800mg tablets (Abbott Laboratories Ltd) |
| 33994 | Diclofenac sodium 25mg gastro-resistant tablets (IVAX Pharmaceuticals UK Ltd) |
| 34910 | Mefenamic acid 500mg Tablet (Berk Pharmaceuticals Ltd) |
| 17030 | Rhumalgan SR 75mg capsules (Sandoz Ltd) |
| 51339 | Indometacin 25mg capsules (Genesis Pharmaceuticals Ltd) |
| 17029 | Rhumalgan CR 75 tablets (Sandoz Ltd) |
| 1233 | Diclofenac sodium 75mg modified-release tablets |
| 8645 | Aspirin 300mg effervescent tablets |
| 57370 | Meloxicam 15mg orodispersible tablets sugar free |
| 42821 | Nabumetone 500mg tablets (A A H Pharmaceuticals Ltd) |
| 1983 | Mefenamic acid 250mg Dispersible tablet |
| 42397 | Nurofen Express 256mg tablets (Reckitt Benckiser Healthcare (UK) Ltd) |
| 1246 | Ponstan 250mg Dispersible tablet (Chemidex Pharma Ltd) |
| 15005 | Indomod 25mg modified-release capsules (Pfizer Ltd) |
| 53345 | Voltarol Rapid 50mg tablets (Lexon (UK) Ltd) |
| 34485 | Aspirin 75mg gastro-resistant tablets (IVAX Pharmaceuticals UK Ltd) |
| 586 | Ibuprofen 200mg Capsule |
| 41366 | Axorid 100mg/20mg modified-release capsules (Meda Pharmaceuticals Ltd) |
| 3817 | Synflex 275mg tablets (Roche Products Ltd) |
| 26351 | Rheumatac Retard 75 tablets (Amdipharm Plc) |
| 13818 | Nabumetone 500mg tablets (Actavis UK Ltd) |
| 4648 | Ibuspray 5% spray (Dermal Laboratories Ltd) |
| 14422 | Fenbufen 450mg Effervescent tablet |
| 41621 | Piroxicam 20mg capsules (A A H Pharmaceuticals Ltd) |
| 2257 | Surgam SA 300mg capsules (Sanofi) |
| 8062 | Motifene 75mg modified-release capsules (Daiichi Sankyo UK Ltd) |
| 32854 | Diclofenac sodium 75mg modified-release capsules (A A H Pharmaceuticals Ltd) |
| 51874 | Arcoxia 30mg tablets (Lexon (UK) Ltd) |
| 48871 | Diclofenac potassium 25mg tablets (Actavis UK Ltd) |
| 3897 | Sulindac 100mg tablets |
| 3334 | Ketorolac 0.5% eye drops |
| 26242 | Timpron 250mg Tablet (Berk Pharmaceuticals Ltd) |
| 52154 | Ibuprofen 200mg tablets (Galpharm International Ltd) |
| 23026 | Artracin sr 75mg Modified-release capsule (Trinity Pharmaceuticals Ltd) |
| 645 | Aspirin 300mg suppositories |
| 32090 | Mefenamic acid 500mg tablets (Actavis UK Ltd) |
| 10209 | Ibufem 200mg tablets (Galpharm International Ltd) |
| 37850 | Ibucalm Ibuprofen Pain Relief 5% gel (Aspar Pharmaceuticals Ltd) |
| 55233 | Ibuprofen 400mg Tablet (Nucare Plc) |
| 1073 | Mefenamic acid 500mg tablets |
| 45814 | First Resort Double Action Pain Relief 12.5mg tablets (Actavis UK Ltd) |
| 4469 | Fenoprofen 300mg tablets |
| 19382 | Slofenac 75mg SR tablets (Sterwin Medicines) |
| 499 | Diclofenac 50mg suppositories |
| 11970 | Meloxicam 7.5mg suppositories |
| 13639 | Flexin-50 Continus tablets (Napp Pharmaceuticals Ltd) |
| 44800 | Naproxen 500mg / Esomeprazole 20mg modified-release tablets |
| 17680 | Indomax 75 SR capsules (Ashbourne Pharmaceuticals Ltd) |
| 24137 | Indometacin 25mg capsules (Actavis UK Ltd) |
| 36329 | Ibuprofen 10% gel (Thornton & Ross Ltd) |
| 13627 | Mobic 15mg suppositories (Boehringer Ingelheim Ltd) |
| 11168 | Volsaid Retard 100 tablets (Chiesi Ltd) |
| 4045 | Naprosyn EC 375mg tablets (Roche Products Ltd) |
| 12776 | Ibumousse 5% (Dermal Laboratories Ltd) |
| 11215 | Voltarol 25mg suppositories (Novartis Pharmaceuticals UK Ltd) |
| 26247 | Opustan 500mg Tablet (Opus Pharmaceuticals Ltd) |
| 22230 | Meflam 250mg Capsule (Trinity Pharmaceuticals Ltd) |
| 40516 | Anadin LiquiFast 200mg capsules (Pfizer Consumer Healthcare Ltd) |
| 14517 | Robaxisal forte Tablet (Shire Pharmaceuticals Ltd) |
| 52618 | Aspirin 75mg dispersible tablets (Bristol Laboratories Ltd) |
| 33785 | Galprofen 200mg tablets (Galpharm International Ltd) |
| 32875 | Ibuprofen 400mg tablets (Sandoz Ltd) |
| 30942 | Diclofenac 50mg Tablet (Regent Laboratories Ltd) |
| 31916 | Tiloket CR 100mg capsules (Tillomed Laboratories Ltd) |
| 33293 | Aspirin 75mg gastro-resistant tablets (Sterwin Medicines) |
| 54906 | Diclofenac 50mg/5ml oral suspension |
| 56213 | Ibuprofen 400mg tablets sugar coated (Kent Pharmaceuticals Ltd) |
| 10265 | Fenbid 5% gel (Mercury Pharma Group Ltd) |
| 48644 | Ibuprofen 400mg caplets (Lloyds Pharmacy Ltd) |
| 6249 | Froben 100mg tablets (Abbott Laboratories Ltd) |
| 332 | Ibuprofen 5% gel |
| 55505 | Naproxen 250mg gastro-resistant tablets (Kent Pharmaceuticals Ltd) |
| 8401 | Motrin 400mg tablets (Pfizer Ltd) |
| 41624 | Piroxicam 10mg capsules (IVAX Pharmaceuticals UK Ltd) |
| 1115 | Diclofenac sodium 100mg modified-release capsules |
| 12992 | Aspirin 500mg / Codeine 8mg dispersible tablets sugar free |
| 23425 | Nurofen Migraine Pain 342mg tablets (Reckitt Benckiser Healthcare (UK) Ltd) |
| 25342 | Arthrosin EC 500 tablets (Ashbourne Pharmaceuticals Ltd) |
| 32210 | Aspirin 300mg dispersible tablets (Actavis UK Ltd) |
| 54760 | Parecoxib 40mg powder and solvent for solution for injection vials |
| 20059 | Tiaprofenic acid 300mg sachets |
| 18647 | Fenoket 200mg modified-release capsules (Opus Pharmaceuticals Ltd) |
| 32097 | Indometacin 75mg Modified-release capsule (Actavis UK Ltd) |
| 34289 | Naproxen 250mg gastro-resistant tablets (Generics (UK) Ltd) |
| 28348 | Ibuprofen 200mg tablets (Teva UK Ltd) |
| 49432 | Calprofen 100mg/5ml oral suspension (McNeil Products Ltd) |
| 10558 | Flexin-75 Continus tablets (Napp Pharmaceuticals Ltd) |
| 9474 | Preservex 100mg tablets (Almirall Ltd) |
| 32862 | Ibuprofen 100mg/5ml oral suspension sugar free (Thornton & Ross Ltd) |
| 17201 | Motrin 600mg tablets (Pfizer Ltd) |
| 35882 | Diclofenac 0.1% eye drops 0.3ml unit dose preservative free |
| 2827 | Feldene 10mg dispersible tablets (Pfizer Ltd) |
| 21380 | Aspirin 75mg / Isosorbide mononitrate 60mg modified-release tablets |
| 16170 | Fenbufen 300mg capsules (Genus Pharmaceuticals Ltd) |
| 48071 | Piroxicam 0.50% Gel (Manx Pharma Ltd) |
| 25341 | Arthrosin EC 250 tablets (Ashbourne Pharmaceuticals Ltd) |
| 57943 | Valket 200 Retard capsules (Tillomed Laboratories Ltd) |
| 2258 | Emflex 60mg capsules (Merck Serono Ltd) |
| 2628 | Nu-seals aspirin ec 75mg Gastro-resistant tablet (Eli Lilly and Company Ltd) |
| 31499 | Paracetamol with aspirin tablet |
| 21949 | Toradol 30mg/1ml solution for injection ampoules (Roche Products Ltd) |
| 34911 | Ibuprofen 200mg Tablet (Celltech Pharma Europe Ltd) |
| 14476 | Indolar SR 75mg capsules (Sandoz Ltd) |
| 38770 | Lodine SR 600mg tablets (Almirall Ltd) |
| 1470 | Mobic 15mg tablets (Boehringer Ingelheim Ltd) |
| 3599 | Ibuprofen 600mg effervescent granules sachets |
| 2288 | Naprosyn 250mg tablets (Roche Products Ltd) |
| 51808 | Diclofenac 12.5mg/5ml oral solution |
| 34309 | Aspirin 300mg dispersible tablets (A A H Pharmaceuticals Ltd) |
| 3852 | Diclomax 100mg Modified-release capsule (Provalis Healthcare Ltd) |
| 3266 | Flurbiprofen 50mg tablets |
| 2463 | Piroxicam 10mg dispersible tablets |
| 30920 | Aspirin 300mg Dispersible tablet (M & A Pharmachem Ltd) |
| 37562 | Arcoxia 30mg tablets (Merck Sharp & Dohme Ltd) |
| 57057 | Aspirin 75mg dispersible tablets (Wockhardt UK Ltd) |
| 42218 | Co-codaprin 8mg/400mg dispersible tablets (A A H Pharmaceuticals Ltd) |
| 30297 | Diclofenac 50mg Gastro-resistant tablet (Pharmacia Ltd) |
| 32136 | Ibular 200mg Tablet (Lagap) |
| 34354 | Ibuprofen 200mg tablets (Vantage) |
| 39876 | Mobigel 4% spray (Mercury Pharma Group Ltd) |
| 30724 | Galprofen 100mg/5ml oral suspension (Galpharm International Ltd) |
| 14570 | Ibuleve 5% Mousse (Dendron Ltd) |
| 4095 | Voltarol 12.5mg Suppository (Novartis Pharmaceuticals UK Ltd) |
| 52931 | Naproxen 500mg gastro-resistant tablets (Kent Pharmaceuticals Ltd) |
| 33668 | Aspirin 300mg Dispersible tablet (Rusco Ltd) |
| 54997 | Aspirin 75mg dispersible tablets (Dowelhurst Ltd) |
| 32227 | Larafen CR 200mg capsules (Ennogen Pharma Ltd) |
| 38817 | Diclofenac potassium 12.5mg tablets |
| 57475 | Meloxicam 7.5mg orodispersible tablets sugar free |
| 54476 | Naproxen 500mg gastro-resistant tablets (Genesis Pharmaceuticals Ltd) |
| 24121 | Diclofenac sodium 25mg gastro-resistant tablets (Actavis UK Ltd) |
| 3311 | Etodolac 200mg capsules |
| 34487 | Diclofenac sodium 50mg gastro-resistant tablets (IVAX Pharmaceuticals UK Ltd) |
| 53711 | Aspirin 300mg Tablet (Nucare Plc) |
| 37972 | Ibuleve Speed Relief 5% gel (Dendron Ltd) |
| 39708 | Diclofenac 4% cutaneous spray |
| 17126 | Fenactol SR 75mg tablets (Discovery Pharmaceuticals Ltd) |
| 1116 | Diclofenac 100mg suppositories |
| 8600 | Piroxicam 20mg suppositories |
| 25619 | Nurofen 400mg Tablet (Crookes Healthcare Ltd) |
| 10625 | Indocid 5mg/ml oral suspension (Merck Sharp & Dohme Ltd) |
| 570 | Dynastat 40mg Powder for solution for injection (Pharmacia Ltd) |
| 52338 | Diclofenac potassium 50mg tablets (Focus Pharmaceuticals Ltd) |
| 50949 | Aspirin 75mg tablets (A A H Pharmaceuticals Ltd) |
| 47350 | Voltarol Active 4% spray (Novartis Consumer Health UK Ltd) |
| 1708 | Codafen Continus tablets (Napp Pharmaceuticals Ltd) |
| 56078 | Rhumalgan XL 100mg capsules (Almus Pharmaceuticals Ltd) |
| 21824 | Flamrase 50 EC tablets (Teva UK Ltd) |
| 25205 | Ibuprofen 100mg/5ml oral suspension 5ml sachets sugar free (Thornton & Ross Ltd) |
| 48000 | Aspirin 300mg tablets (Sigma Pharmaceuticals Plc) |
| 21813 | Lidifen 400mg Tablet (Berk Pharmaceuticals Ltd) |
| 21955 | Ketozip 200 XL capsules (Ashbourne Pharmaceuticals Ltd) |
| 35292 | Nurofen 200mg liquid capsules (Reckitt Benckiser Healthcare (UK) Ltd) |
| 2200 | Indometacin 25mg modified-release capsules |
| 28553 | Diclofenac sodium 50mg gastro-resistant tablets (Teva UK Ltd) |
| 23841 | Safapryn Tablet (Pfizer Ltd) |
| 43679 | Flamasacard 162.5mg Modified-release capsule (Abbey Pharmaceuticals Ltd) |
| 42455 | Dicloflex Retard 100mg tablets (Teva UK Ltd) |
| 33676 | Aspirin 75mg dispersible tablets (Kent Pharmaceuticals Ltd) |
| 51343 | Voltarol Rapid 25mg tablets (Doncaster Pharmaceuticals Ltd) |
| 29332 | Ibuprofen 100mg/5ml oral suspension sugar free (Sandoz Ltd) |
| 11495 | Piroxicam betadex 20mg tablets |
| 16193 | Motrin 800mg tablets (Pfizer Ltd) |
| 17128 | Fenactol 50mg gastro-resistant tablets (Discovery Pharmaceuticals Ltd) |
| 3335 | Acular 0.5% eye drops (Allergan Ltd) |
| 45213 | Diclofenac 10mg dispersible tablets |
| 580 | Diclofenac sodium 75mg modified-release tablets |
| 18921 | Fenactol 25mg gastro-resistant tablets (Discovery Pharmaceuticals Ltd) |
| 20466 | Voltarol Ophtha Multidose 0.1% eye drops (Spectrum Thea Pharmaceuticals Ltd) |
| 3170 | Meloxicam 15mg suppositories |
| 45262 | Naproxen Oral solution |
| 19575 | Proflex 200mg Tablet (Novartis Consumer Health UK Ltd) |
| 8185 | Disprin CV 300mg modified-release tablets (Reckitt Benckiser Healthcare (UK) Ltd) |
| 29054 | Methocarbamol with aspirin Tablet |
| 48675 | Ibuprofen Pain Relief Maximum Strength 10% gel (Numark Management Ltd) |
| 28390 | Valenac ec 25mg Gastro-resistant tablet (Shire Pharmaceuticals Ltd) |
| 34850 | Ibuprofen 600mg tablets (Teva UK Ltd) |
| 51828 | Ibuprofen 100mg/5ml oral suspension sugar free (Kent Pharmaceuticals Ltd) |
| 49799 | Aspirin 150mg suppositories (A A H Pharmaceuticals Ltd) |
| 55454 | Naproxen 500mg tablets (Kent Pharmaceuticals Ltd) |
| 10481 | Lederfen f 450mg Tablet (Wyeth Pharmaceuticals) |
| 56925 | Naproxen 250mg tablets (Actavis UK Ltd) |
| 18371 | Digenac xl 100mg Modified-release tablet (Genus Pharmaceuticals Ltd) |
| 34621 | Ibuprofen 200mg Tablet (Nucare Plc) |
| 37235 | Ibuprofen 100mg/5ml / Pseudoephedrine 15mg/5ml oral suspension sugar free |
| 49059 | Voltarol 50mg dispersible tablets (Lexon (UK) Ltd) |
| 30282 | Diclofenac 75mg Modified-release tablet (Galen Ltd) |
| 49060 | Aspirin 75mg dispersible tablets (Alliance Healthcare (Distribution) Ltd) |
| 27200 | Diclovol Retard 100mg tablets (Generics (UK) Ltd) |
| 2243 | Meloxicam 7.5mg tablets |
| 34143 | Naprosyn 375 Tablet (Roche Products Ltd) |
| 9630 | Feldene P 0.5% gel (Pfizer Ltd) |
| 47816 | Tenoxicam 20mg Tablet (Sovereign Medical Ltd) |
| 1043 | Naproxen sodium 275mg tablets |
| 1137 | Nu-seals aspirin ec 300mg Gastro-resistant tablet (Eli Lilly and Company Ltd) |
| 10792 | Voltarol 50mg Suppository (Novartis Pharmaceuticals UK Ltd) |
| 10939 | Toradol 10mg/1ml solution for injection ampoules (Roche Products Ltd) |
| 25362 | Defanac 25mg gastro-resistant tablets (Ranbaxy (UK) Ltd) |
| 14251 | Ketorolac 30mg/1ml solution for injection ampoules |
| 54734 | Aspirin 300mg tablets (Wockhardt UK Ltd) |
| 49277 | Ibuprofen 200mg caplets (Bristol Laboratories Ltd) |
| 1210 | Indometacin 75mg modified-release capsules |
| 36606 | Manorfen 400mg tablets (The Manor Drug Company (Nottingham) Ltd) |
| 41823 | Indometacin sr 75mg Modified-release capsule (Generics (UK) Ltd) |
| 56282 | Diclofenac 2% gel |
| 157 | Voltarol 100mg Suppository (Novartis Pharmaceuticals UK Ltd) |
| 1096 | Diclofenac sodium 25mg gastro-resistant tablets |
| 40664 | Oruvail 150 modified-release capsules (Sanofi) |
| 838 | Oruvail 200mg Modified-release capsule (Hawgreen Ltd) |
| 1075 | Diclofenac sodium 50mg gastro-resistant tablets |
| 16272 | Lofensaid Retard 100 tablets (Opus Pharmaceuticals Ltd) |
| 34942 | Aspirin 75mg Dispersible tablet (Nucare Plc) |
| 9201 | Ibuleve 5% spray (Dendron Ltd) |
| 33589 | Ibuprofen 400mg tablets (Thornton & Ross Ltd) |
| 46919 | Ketoprofen sr 200mg Capsule (Approved Prescription Services Ltd) |
| 16611 | Anadin Tablet (Wyeth Consumer Healthcare) |
| 50269 | Arthrotec 75 gastro-resistant tablets (Mawdsley-Brooks & Company Ltd) |
| 32704 | Advil cold and sinus 200mg+30mg Tablet (Wyeth Consumer Healthcare) |
| 39502 | Ibuprofen sodium dihydrate 200mg tablets |
| 42406 | Diclofenac 50mg Gastro-resistant tablet (Almus Pharmaceuticals Ltd) |
| 43096 | Ibuleve Speed Relief 5% spray (Dendron Ltd) |
| 25790 | Rhumalgan 25mg Tablet (Lagap) |
| 18030 | Imazin XL forte tablets (Napp Pharmaceuticals Ltd) |
| 33111 | Prosaid 250mg Tablet (BHR Pharmaceuticals Ltd) |
| 2105 | Solprin 300mg Tablet (Reckitt Benckiser Healthcare (UK) Ltd) |
| 16194 | Lodine 200mg Tablet (Shire Pharmaceuticals Ltd) |
| 28332 | Mobiflex 20mg powder and solvent for solution for injection vials (Roche Products Ltd) |
| 2938 | Ibuprofen 100mg/5ml Oral suspension |
| 7222 | Tolfenamic acid 200mg tablets |
| 389 | Ketoprofen 50mg capsules |
| 37253 | Anadin ultra double strength 400mg Capsule (Wyeth Consumer Healthcare) |
| 56106 | Naproxen 500mg/5ml oral suspension |
| 58112 | Alka-Seltzer effervescent tablets original (Bayer Plc) |
| 1778 | Surgam 300mg Tablet (Sanofi) |
| 14901 | Diclofenac 1% transdermal patches |
| 24212 | Imbrilon 50mg Capsule (Berk Pharmaceuticals Ltd) |
| 29587 | Ebufac 400mg Tablet (DDSA Pharmaceuticals Ltd) |
| 11466 | Nabumetone 500mg/5ml oral suspension sugar free |
| 685 | Aspav dispersible tablets (Actavis UK Ltd) |
| 21811 | Lidifen 200mg Tablet (Berk Pharmaceuticals Ltd) |
| 24960 | Aspirin 300mg tablets (Vantage) |
| 31953 | Aspirin 75mg dispersible tablets (IVAX Pharmaceuticals UK Ltd) |
| 40144 | Aspirin 300mg Dispersible tablet (Thornton & Ross Ltd) |
| 36486 | Econac XL 100mg tablets (Mercury Pharma Group Ltd) |
| 48084 | Ibuprofen 200mg/5ml oral suspension |
| 15068 | Arthrofen 400 tablets (Ashbourne Pharmaceuticals Ltd) |
| 43911 | Ibuprofen 600mg Tablet (C P Pharmaceuticals Ltd) |
| 4692 | Dicloflex 50mg gastro-resistant tablets (Dexcel-Pharma Ltd) |
| 46440 | Naproxen 500mg Tablet (M & A Pharmachem Ltd) |
| 54353 | Generic Anadin Extra soluble tablets sugar free |
| 51099 | Voltarol Rapid 50mg tablets (Mawdsley-Brooks & Company Ltd) |
| 21421 | Seractil 400mg tablets (Genus Pharmaceuticals Ltd) |
| 5767 | Ibuprofen 10% gel |
| 24128 | Diclofenac sodium 25mg gastro-resistant tablets (A A H Pharmaceuticals Ltd) |
| 141 | Piroxicam 10mg capsules |
| 26214 | Fenbuzip 450mg Tablet (Ashbourne Pharmaceuticals Ltd) |
| 8789 | Dicloflex retard tabs 100 100mg Modified-release tablet (Dexcel-Pharma Ltd) |
| 6208 | Voltarol 1% Emulgel P (Novartis Consumer Health UK Ltd) |
| 21807 | Flamrase 25 EC tablets (Teva UK Ltd) |
| 45320 | Ibuprofen 200mg tablets (Sandoz Ltd) |
| 14380 | Lederfen 300mg capsules (Mercury Pharma Group Ltd) |
| 26404 | Tolmetin 200mg Capsule |
| 2904 | Diclofenac sodium 75mg gastro-resistant modified-release capsules |
| 1621 | Brufen 200mg tablets (Abbott Laboratories Ltd) |
| 54514 | Ibuprofen lysine 400mg oral powder sachets |
| 8385 | Ketoprofen 150mg modified-release capsules |
| 34762 | Aspirin 300mg Gastro-resistant tablet (Galen Ltd) |
| 4710 | Mefenamic acid 250mg Capsule (Actavis UK Ltd) |
| 4564 | Fenoprofen 200mg Tablet |
| 21444 | Volraman 25mg gastro-resistant tablets (LPC Medical (UK) Ltd) |
| 39738 | Aspirin 162.5mg modified-release capsules |
| 24236 | Slofenac 100mg Modified-release tablet (Sterwin Medicines) |
| 9939 | Aspirin 500mg effervescent tablets sugar free |
| 46860 | Anadin LiquiFast 200mg effervescent tablets (Pfizer Consumer Healthcare Ltd) |
| 11951 | Original Phensic Aspirin tablets (Merck Consumer Health Products) |
| 19036 | Arthrofen 200 tablets (Ashbourne Pharmaceuticals Ltd) |
| 47820 | Voltarol Pain-eze Extra Strength 25mg tablets (Novartis Consumer Health UK Ltd) |
| 19320 | Piroflam 20mg Capsule (Opus Pharmaceuticals Ltd) |
| 17754 | Progesic 200mg Tablet (Eli Lilly and Company Ltd) |
| 11995 | Orudis 100mg Suppository (Hawgreen Ltd) |
| 28810 | Aspirin 300mg with Glycine 133mg soluble tablets |
| 34924 | Mefenamic acid 250mg Capsule (Teva UK Ltd) |
| 43806 | Aspirin 300mg gastro-resistant tablets (Sandoz Ltd) |
| 497 | Voltarol 25mg gastro-resistant tablets (Novartis Pharmaceuticals UK Ltd) |
| 18448 | Voltarol 12.5mg suppositories (Novartis Pharmaceuticals UK Ltd) |
| 54518 | Diclofenac sodium 50mg gastro-resistant tablets (Phoenix Healthcare Distribution Ltd) |
| 32366 | Relcofen 200mg Tablet (Actavis UK Ltd) |
| 31178 | Ketoprofen 2.5% gel (A A H Pharmaceuticals Ltd) |
| 20442 | Nurofen 5% gel (Reckitt Benckiser Healthcare (UK) Ltd) |
| 21864 | Pirozip 10 capsules (Ashbourne Pharmaceuticals Ltd) |
| 55579 | Aspirin 300mg tablets (Almus Pharmaceuticals Ltd) |
| 22618 | Solprin 75mg Tablet (Reckitt Benckiser Healthcare (UK) Ltd) |
| 3432 | Naproxen 375mg gastro-resistant tablets |
| 4043 | Froben sr 200mg Modified-release capsule (Abbott Laboratories Ltd) |
| 26159 | Fenbid Forte 10% gel (Mercury Pharma Group Ltd) |
| 37502 | Ibuprofen 10mg/2ml solution for infusion ampoules |
| 43616 | Celecoxib 400mg capsules |
| 9500 | Diclotard 75mg modified-release tablets (Galen Ltd) |
| 26888 | Difenor xl 100mg Modified-release tablet (IVAX Pharmaceuticals UK Ltd) |
| 15367 | Anadin Extra tablets (Pfizer Consumer Healthcare Ltd) |
| 16192 | Motrin 200mg Tablet (Pharmacia Ltd) |
| 48810 | Dysman 250 capsules (Ashbourne Pharmaceuticals Ltd) |
| 7522 | Lederfen 300mg Tablet (Wyeth Pharmaceuticals) |
| 30243 | Ibuprofen 200mg effervescent tablets |
| 52956 | Voltarol 1% Emulgel (Stephar (U.K.) Ltd) |
| 56651 | Piroxicam 0.5% gel (Sigma Pharmaceuticals Plc) |
| 46968 | Mefenamic acid 250mg capsules (Generics (UK) Ltd) |
| 53384 | Voltarol 50mg dispersible tablets (Mawdsley-Brooks & Company Ltd) |
| 34610 | Naproxen 500mg gastro-resistant tablets (Generics (UK) Ltd) |
| 26994 | Fenbuzip 300mg Tablet (Ashbourne Pharmaceuticals Ltd) |
| 33704 | Ibuprofen 100mg/5ml oral suspension sugar free (A A H Pharmaceuticals Ltd) |
| 25358 | Defanac 50mg gastro-resistant tablets (Ranbaxy (UK) Ltd) |
| 402 | Nurofen 200mg Tablet (Crookes Healthcare Ltd) |
| 29345 | Ibuprofen 100mg/5ml Oral suspension (Hillcross Pharmaceuticals Ltd) |
| 344 | Acemetacin 60mg capsules |
| 676 | Diclofenac 75mg/3ml solution for injection ampoules |
| 34611 | Aspirin 75mg gastro-resistant tablets (C P Pharmaceuticals Ltd) |
| 34663 | Ibuprofen 100mg/5ml Oral suspension (Neo Laboratories Ltd) |
| 42500 | Ketoprofen sr 100mg Capsule (Approved Prescription Services Ltd) |
| 31589 | Diclofenac sodium 75mg modified-release tablets (A A H Pharmaceuticals Ltd) |
| 8663 | Naprosyn S/R 500mg tablets (Roche Products Ltd) |
| 38948 | Diclomax Retard 100mg capsules (Galen Ltd) |
| 44892 | Sudafed sinus pressure & pain Tablet (McNeil Products Ltd) |
| 54430 | Aspirin 75mg tablets (Alliance Healthcare (Distribution) Ltd) |
| 48021 | Aspirin 75mg Tablet (Hillcross Pharmaceuticals Ltd) |
| 41450 | Orudis 100mg suppositories (Sanofi) |
| 11322 | Flamrase sr 75mg Modified-release tablet (APS Berk) |
| 52009 | Ibuprofen 200mg capsules (Galpharm International Ltd) |
| 20386 | Ramodar 200mg Tablet (Wyeth Pharmaceuticals) |
| 850 | Mobic 7.5mg tablets (Boehringer Ingelheim Ltd) |
| 33318 | Indometacin 50mg Capsule (Generics (UK) Ltd) |
| 41817 | Indometacin sr 75mg Modified-release capsule (C P Pharmaceuticals Ltd) |
| 1392 | Ibuprofen 800mg modified-release tablets |
| 15 | Ibuprofen 400mg tablets |
| 25794 | Isisfen 400mg Tablet (Isis Products Ltd) |
| 3053 | Naproxen 500mg gastro-resistant tablets |
| 3168 | Indometacin 25mg/5ml oral suspension sugar free |
| 33656 | Aspirin 75mg dispersible tablets (A A H Pharmaceuticals Ltd) |
| 30849 | Valdic 75 Retard tablets (Fannin UK Ltd) |
| 44639 | Aspirin 300mg Dispersible tablet (Nucare Plc) |
| 19189 | Micropirin 75mg Gastro-resistant tablet (Ratiopharm UK Ltd) |
| 2363 | Dolobid 250mg tablets (Merck Sharp & Dohme Ltd) |
| 7667 | Diclofenac 12.5mg suppositories |
| 34550 | Ibuprofen 400mg tablets film coated (Actavis UK Ltd) |
| 50652 | Junior Ibuprofen 100mg/5ml oral suspension (Numark Management Ltd) |
| 38182 | Orbifen Cold & Flu oral suspension (Orbis Consumer Products Ltd) |
| 30168 | Arthroxen 250mg Tablet (C P Pharmaceuticals Ltd) |
| 48974 | Aspirin 75mg tablets (Phoenix Healthcare Distribution Ltd) |
| 41594 | Aspirin 300mg Dispersible tablet (Teva UK Ltd) |
| 17733 | Condrotec 500mg+200microgram Tablet (Pharmacia Ltd) |
| 4565 | Fenoprofen 600mg tablets |
| 33457 | Isclofen 50mg Gastro-resistant tablet (Isis Products Ltd) |
| 32509 | Anadin Ibuprofen 200mg tablets (Pfizer Consumer Healthcare Ltd) |
| 51360 | Naproxen 250mg tablets (Accord Healthcare Ltd) |
| 42905 | Diclofenac 75mg Modified-release tablet (Actavis UK Ltd) |
| 5173 | Dexketoprofen 25mg tablets |
| 48326 | Ibuprofen 100mg/5ml oral suspension sugar free |
| 773 | Misoprostol 200microgram tablets |
| 39693 | Naproxen 200mg/5ml oral suspension |
| 30982 | Naproxen 500mg gastro-resistant tablets (Actavis UK Ltd) |
| 16 | Aspirin 75mg tablets |
| 44703 | Piroxicam 10mg Capsule (Berk Pharmaceuticals Ltd) |
| 34725 | Flurbiprofen 50mg Tablet (Bristol-Myers Squibb Pharmaceuticals Ltd) |
| 15732 | Diclovol 50mg gastro-resistant tablets (Arun Pharmaceuticals Ltd) |
| 24122 | Diclofenac sodium 50mg gastro-resistant tablets (Actavis UK Ltd) |
| 30811 | Proflex 300mg Modified-release capsule (Novartis Consumer Health UK Ltd) |
| 341 | Feldene 10mg capsules (Pfizer Ltd) |
| 24356 | Eccoxolac 300mg capsules (Meda Pharmaceuticals Ltd) |
| 37002 | Nurofen Express 200mg liquid capsules (Reckitt Benckiser Healthcare (UK) Ltd) |
| 1544 | Piroxicam 0.5% gel |
| 27571 | ORUVAIL S/R |
| 46844 | Dicloflex 75mg SR tablets (Actavis UK Ltd) |
| 25750 | Rheuflex 250mg Tablet (Goldshield Pharmaceuticals Ltd) |
| 2671 | Indometacin 50mg modified-release tablets |
| 35893 | Dicloflex Retard 100mg tablets (Almus Pharmaceuticals Ltd) |
| 24020 | Valrox 250mg Tablet (Shire Pharmaceuticals Ltd) |
| 34931 | Ibuprofen 200mg Tablet (Regent Laboratories Ltd) |
| 3935 | Feldene 20 capsules (Pfizer Ltd) |
| 25701 | Ketovail 200mg modified-release capsules (Teva UK Ltd) |
| 10917 | Flamrase SR 100mg tablets (Teva UK Ltd) |
| 32234 | Mefenamic acid 500mg tablets (IVAX Pharmaceuticals UK Ltd) |
| 34190 | Indometacin 75mg modified-release capsules (A A H Pharmaceuticals Ltd) |
| 35711 | Dicloflex 25mg gastro-resistant tablets (Teva UK Ltd) |
| 36597 | Hedex Ibuprofen 200mg tablets (Omega Pharma Ltd) |
| 5266 | Lodine sr 600mg Modified-release tablet (Shire Pharmaceuticals Ltd) |
| 18812 | Nurofen meltlets lemon 200mg Orodispersible tablet (Reckitt Benckiser Healthcare (UK) Ltd) |
| 17828 | DISPRIN CV 100 MG TAB |
| 28764 | Closteril 100mg Modified-release tablet (Pharmalife Healthcare Services Ltd) |
| 9222 | Dicloflex 75mg SR tablets (Dexcel-Pharma Ltd) |
| 53604 | Ibuprofen 200mg capsules (Numark Management Ltd) |
| 12000 | Ketoprofen 100mg suppositories |
| 31962 | Ketpron XL 200mg capsules (Mercury Pharma Group Ltd) |
| 3974 | Tenoxicam 20mg tablets |
| 53626 | Naproxen 500mg gastro-resistant tablets (Alliance Healthcare (Distribution) Ltd) |
| 50785 | Diclofenac sodium 50mg gastro-resistant tablets (Genesis Pharmaceuticals Ltd) |
| 34199 | Indometacin 100mg suppositories (Actavis UK Ltd) |
| 21610 | Rhumalgan CR 100 tablets (Sandoz Ltd) |
| 27677 | Diclofenac 75mg/3ml Injection (Antigen Pharmaceuticals) |
| 38332 | Ibucalm 200mg tablets (Aspar Pharmaceuticals Ltd) |
| 40401 | Naproxen 250mg gastro-resistant tablets (IVAX Pharmaceuticals UK Ltd) |
| 10336 | Ketoprofen 100mg/2ml solution for injection ampoules |
| 5648 | Ibuprofen 200mg orodispersible tablets sugar free |
| 2986 | Co-codaprin 8mg/400mg dispersible tablets |
| 1468 | Ibuprofen 200mg Soluble tablet |
| 37731 | Nurofen Express 342mg caplets (Reckitt Benckiser Healthcare (UK) Ltd) |
| 25257 | Advil 200mg tablets (Wyeth Consumer Healthcare) |
| 38944 | Froben SR 200mg capsules (Abbott Laboratories Ltd) |
| 29181 | Dicloflex 75mg SR tablets (Almus Pharmaceuticals Ltd) |
| 41521 | Indometacin 25mg Capsule (Approved Prescription Services Ltd) |
| 11977 | Aspro clear maximum strength tablets |
| 57585 | Asasantin Retard capsules (Dowelhurst Ltd) |
| 5482 | Sulindac 200mg tablets |
| 32242 | Ibuprofen 400mg tablets (Sterwin Medicines) |
| 10295 | Relifex 500mg/5ml oral suspension (Meda Pharmaceuticals Ltd) |
| 34980 | Ibuprofen 200mg tablets sugar coated (Actavis UK Ltd) |
| 20105 | Dicloflex 25mg Gastro-resistant tablet (Ratiopharm UK Ltd) |
| 4713 | Voltarol 75mg/3ml solution for injection ampoules (Novartis Pharmaceuticals UK Ltd) |
| 628 | Diclofenac potassium 25mg tablets |
| 24531 | Mobiflex 20mg Effervescent tablet (Roche Products Ltd) |
| 39109 | Feldene Melt 20mg tablets (Pfizer Ltd) |
| 32100 | Ibuprofen 600mg tablets (A A H Pharmaceuticals Ltd) |
| 7432 | Oruvail IM 100mg/2ml solution for injection ampoules (Sanofi) |
| 54021 | Voltarol Retard 100mg tablets (Sigma Pharmaceuticals Plc) |
| 43709 | Aspirin 75mg gastro-resistant tablets (Almus Pharmaceuticals Ltd) |
| 34271 | Diclofenac sodium 100mg modified-release tablets (A A H Pharmaceuticals Ltd) |
| 16473 | Relifex 500mg dispersible tablets (Meda Pharmaceuticals Ltd) |
| 1139 | Voltarol 25mg Tablet (Novartis Pharmaceuticals UK Ltd) |
| 9736 | Mefenamic acid 50mg/5ml oral suspension |
| 2858 | Feldene 0.5% gel (Pfizer Ltd) |
| 52420 | Celebrex 100mg capsules (Mawdsley-Brooks & Company Ltd) |
| 24320 | Indolar 50mg Capsule (Lagap) |
| 46904 | Nuromol 200mg/500mg tablets (Reckitt Benckiser Healthcare (UK) Ltd) |
| 27013 | Tiloket 200mg Modified-release capsule (Tillomed Laboratories Ltd) |
| 19398 | Radian B Ibuprofen 5% gel (Thornton & Ross Ltd) |
| 17920 | Disprin cv 100mg Modified-release tablet (Reckitt Benckiser Healthcare (UK) Ltd) |
| 2693 | Proflex 5% cream (Novartis Consumer Health UK Ltd) |
| 31054 | Phorpain Maximum Strength 10% gel (Mercury Pharma Group Ltd) |
| 6464 | Arcoxia 60mg tablets (Merck Sharp & Dohme Ltd) |
| 54075 | Voltarol 50mg dispersible tablets (Stephar (U.K.) Ltd) |
| 50628 | Ibuprofen 400mg caplets (The Boots Company Plc) |
| 6007 | Nu-Seals 300 gastro-resistant tablets (Alliance Pharmaceuticals Ltd) |
| 54565 | Aspirin 75mg dispersible tablets (Lloyds Pharmacy Ltd) |
| 34889 | Ibuprofen 400mg Tablet (Celltech Pharma Europe Ltd) |
| 2607 | Paynocil Tablet (Beecham Research Laboratories) |
| 16225 | Dexomon retard 100mg Modified-release tablet (Hillcross Pharmaceuticals Ltd) |
| 9421 | Powergel 2.5% gel (A. Menarini Farmaceutica Internazionale SRL) |
| 28255 | Naproxen 250mg tablets (Wockhardt UK Ltd) |
| 120 | Indocid 25mg capsules (Merck Sharp & Dohme Ltd) |
| 23323 | Prosaid 500mg Tablet (BHR Pharmaceuticals Ltd) |
| 53164 | Diclofenac sodium 25mg gastro-resistant tablets (Kent Pharmaceuticals Ltd) |
| 40394 | Advil 400mg Tablet (Wyeth Consumer Healthcare) |
| 41701 | Ibuprofen 600mg tablets (Actavis UK Ltd) |
| 55313 | Ibuprofen 400mg tablets (Boston Healthcare Ltd) |
| 7261 | Cuprofen 5% gel (SSL International Plc) |
| 51242 | Naproxen 500mg tablets (Pfizer Ltd) |
| 6435 | Pennsaid 16mg/ml cutaneous solution (Movianto UK Ltd) |
| 36521 | Aspirin 500mg modified-release tablets |
| 11326 | Meprobamate with ethoheptazine citrate and aspirin Tablet |
| 26095 | Ibuprofen lysine 400mg tablets |
| 417 | Diclofenac 50mg dispersible tablets sugar free |
| 25800 | Feverfen 100mg/5ml oral suspension (Wise Pharmaceuticals Ltd) |
| 42003 | Indometacin sr 75mg Capsule (Lagap) |
| 29232 | Care ibuprofen 5% Gel (Thornton & Ross Ltd) |
| 32536 | Diclofenac 25mg Tablet (Berk Pharmaceuticals Ltd) |
| 49220 | Aspirin 300mg tablets (Kent Pharmaceuticals Ltd) |
| 30382 | Ibuprofen 200mg Tablet (C P Pharmaceuticals Ltd) |
| 21816 | Pranoxen continus 500mg Tablet (Napp Pharmaceuticals Ltd) |
| 33320 | Aspirin 75mg Dispersible tablet (Sovereign Medical Ltd) |
| 34666 | Aspirin ec 300mg Gastro-resistant tablet (A A H Pharmaceuticals Ltd) |
| 11980 | Cuprofen 400mg Tablet (SSL International Plc) |
| 56007 | Aspirin 300mg dispersible tablets (Sigma Pharmaceuticals Plc) |
| 43060 | Aspirin 300mg Soluble tablet (Celltech Pharma Europe Ltd) |
| 21840 | Arthrosin 250 tablets (Ashbourne Pharmaceuticals Ltd) |
| 4880 | Diclofenac sodium 75mg gastro-resistant / Misoprostol 200microgram tablets |
| 25329 | Lofensaid 25mg gastro-resistant tablets (Opus Pharmaceuticals Ltd) |
| 34425 | Ibuprofen 400mg Tablet (Family Health) |
| 381 | Anadin Tablet (Wyeth Consumer Healthcare) |
| 57523 | Feldene 0.5% gel (Dowelhurst Ltd) |
| 4368 | Lodine 200mg Capsule (Shire Pharmaceuticals Ltd) |
| 37763 | Diclofenac 75mg/2ml solution for injection vials |
| 21382 | Aspirin 150mg / Isosorbide mononitrate 60mg modified-release tablets |
| 647 | Ibuprofen 100mg/5ml oral suspension |
| 41513 | Ibuprofen 200mg tablets (IVAX Pharmaceuticals UK Ltd) |
| 17572 | Tenoxicam 20mg powder and solvent for solution for injection vials |
| 24622 | Aspirin 325mg / Caffeine 22mg tablets |
| 140 | Naproxen 500mg suppositories |
| 25335 | PostMI 75 EC tablets (Ashbourne Pharmaceuticals Ltd) |
| 34218 | Diclofenac 25mg Gastro-resistant tablet (Pharmacia Ltd) |
| 129 | Naprosyn 500mg suppositories (Roche Products Ltd) |
| 27366 | Naproxen 500mg gastro-resistant tablets (Teva UK Ltd) |
| 589 | Voltarol 50mg dispersible tablets (Novartis Pharmaceuticals UK Ltd) |
| 1692 | Diclofenac sodium 50mg gastro-resistant / Misoprostol 200microgram tablets |
| 2235 | Relifex 500mg tablets (Meda Pharmaceuticals Ltd) |
| 3336 | Toradol 10mg tablets (Roche Products Ltd) |
| 52714 | Etodolac 600mg modified-release tablets (Alliance Healthcare (Distribution) Ltd) |
| 18234 | Rheumacin LA 75mg capsules (Hillcross Pharmaceuticals Ltd) |
| 15836 | Ocufen 0.03% eye drops 0.4ml unit dose (Allergan Ltd) |
| 56996 | Aspirin 75mg dispersible tablets (Waymade Healthcare Plc) |
| 37094 | Cuprofen 200mg tablets (SSL International Plc) |
| 26234 | Flamatrol 10mg Capsule (Berk Pharmaceuticals Ltd) |
| 4631 | Voltarol 50mg gastro-resistant tablets (Novartis Pharmaceuticals UK Ltd) |
| 39722 | Voltarol Pain-eze 12.5mg tablets (Novartis Consumer Health UK Ltd) |
| 20840 | Acetylsalicylic acid mix |
| 37750 | Piroxicam 20mg capsules (Generics (UK) Ltd) |
| 53803 | Ibuprofen 200mg capsules (Kent Pharmaceuticals Ltd) |
| 4625 | Voltarol 75mg SR tablets (Novartis Pharmaceuticals UK Ltd) |
| 21846 | Pirozip 20 capsules (Ashbourne Pharmaceuticals Ltd) |
| 2382 | Tiaprofenic acid 300mg modified-release capsules |
| 21831 | Dysman 250mg Capsule (Ashbourne Pharmaceuticals Ltd) |
| 56995 | Aspirin 75mg dispersible tablets (Phoenix Healthcare Distribution Ltd) |
| 21814 | ORUVAIL S/R |
| 26205 | Fenbuzip 300mg Capsule (Ashbourne Pharmaceuticals Ltd) |
| 30790 | Dicloflex sr 75mg Tablet (Genus Pharmaceuticals Ltd) |
| 17124 | Dicloflex sr 100mg Tablet (IVAX Pharmaceuticals UK Ltd) |
| 11461 | Ibuprofen 300mg modified-release / Codeine 20mg tablets |
| 9439 | Flurbiprofen 200mg modified-release capsules |
| 16176 | Lederfen 450mg tablets (Mercury Pharma Group Ltd) |
| 24682 | Tenoxicam 20mg effervescent tablets |
| 30389 | Contraflam 250mg Capsule (Berk Pharmaceuticals Ltd) |
| 9301 | Aspirin 100mg modified-release tablets |
| 6006 | Nu-Seals 75 gastro-resistant tablets (Alliance Pharmaceuticals Ltd) |
| 14085 | Diclovol Retard 100mg tablets (Arun Pharmaceuticals Ltd) |
| 2863 | Tiaprofenic acid 300mg tablets |
| 484 | Equagesic Tablet (Wyeth Pharmaceuticals) |
| 31938 | Aspirin 75mg gastro-resistant tablets (Sandoz Ltd) |
| 8145 | Fenbufen 300mg tablets |
| 4309 | Ibuprofen lysine 200mg tablets |
| 33662 | Aspirin 300mg Dispersible tablet (A A H Pharmaceuticals Ltd) |
| 39758 | Nurofen Express 256mg caplets (Reckitt Benckiser Healthcare (UK) Ltd) |
| 44986 | Vimovo 500mg/20mg modified-release tablets (AstraZeneca UK Ltd) |
| 24305 | Ibufac 400mg Tablet (DDSA Pharmaceuticals Ltd) |
| 47992 | Aspirin 75mg gastro-resistant tablets (A A H Pharmaceuticals Ltd) |
| 24887 | Nurofen Advance 200mg tablets (Crookes Healthcare Ltd) |
| 29772 | Ketotard XL 200mg capsules (Galen Ltd) |
| 56883 | Aspirin 75mg tablets (Waymade Healthcare Plc) |
| 21387 | Diclofenac sodium 50mg gastro-resistant tablets (Generics (UK) Ltd) |
| 827 | Voltarol 1% Emulgel (Novartis Consumer Health UK Ltd) |
| 51923 | Ibuprofen 10% gel (A A H Pharmaceuticals Ltd) |
| 7524 | Feldene 20mg dispersible tablets (Pfizer Ltd) |
| 7434 | Clinoril 100mg tablets (Merck Sharp & Dohme Ltd) |
| 34793 | Mefenamic acid 250mg capsules (Zentiva) |
| 34922 | Naproxen 500mg Tablet (Berk Pharmaceuticals Ltd) |
| 447 | Diclofenac sodium 75mg modified-release capsules |
| 8510 | Ibuprofen 5% spray |
| 29848 | Aspirin 300mg with Glycine 150mg chewable tablets |
| 41524 | Mefenamic acid 500mg tablets (Teva UK Ltd) |
| 6853 | Ibutop Ralgex Ibuprofen 5% gel (SSL International Plc) |
| 56584 | Arcoxia 60mg tablets (Lexon (UK) Ltd) |
| 29749 | Ibuprofen 200mg tablets (Ranbaxy (UK) Ltd) |
| 32365 | Relcofen 400mg tablets (Actavis UK Ltd) |
| 47994 | Naproxen 250mg Gastro-resistant tablet (Almus Pharmaceuticals Ltd) |
| 6498 | Arcoxia 90mg tablets (Merck Sharp & Dohme Ltd) |
| 53397 | Brufen 100mg/5ml syrup (Mawdsley-Brooks & Company Ltd) |
| 22206 | Nurofen Long Lasting 300mg capsules (Crookes Healthcare Ltd) |
| 56071 | Voltarol Active 4% spray (Novartis Consumer Health UK Ltd) |
| 56558 | Voltarol 12 Hour 2% Emulgel P (Novartis Consumer Health UK Ltd) |
| 1030 | Junifen 100mg/5ml Oral suspension (Crookes Healthcare Ltd) |
| 1270 | Ibuleve 5% gel (Dendron Ltd) |
| 46921 | Ibuprofen 400mg tablets (Ranbaxy (UK) Ltd) |
| 7426 | Lederfen 300mg Capsule (Wyeth Pharmaceuticals) |
| 52280 | Aspirin 300mg Tablet (Wockhardt UK Ltd) |
| 32108 | Diclofenac sodium 25mg gastro-resistant tablets (Teva UK Ltd) |
| 17525 | Fenactol Retard 100mg tablets (Discovery Pharmaceuticals Ltd) |
| 34797 | Aspirin 75mg gastro-resistant tablets (Actavis UK Ltd) |
| 56275 | Meloxicam 7.5mg tablets (Teva UK Ltd) |
| 3431 | Naproxen 250mg gastro-resistant tablets |
| 3899 | Dolobid 500mg tablets (Merck Sharp & Dohme Ltd) |
| 57162 | Diclofenac 50mg dispersible tablets sugar free (Doncaster Pharmaceuticals Ltd) |
| 38881 | Diclomax SR 75mg capsules (Galen Ltd) |
| 50926 | Aspirin 75mg dispersible tablets (The Boots Company Plc) |
| 361 | DISPRIN TAB |
| 24308 | Slo-Indo 75mg capsules (Generics (UK) Ltd) |
| 15501 | Flurbiprofen 100mg suppositories |
| 27490 | Feldene IM 20mg/1ml solution for injection ampoules (Pfizer Ltd) |
| 55099 | Acoflam 100mg Retard tablets (Mercury Pharma Group Ltd) |
| 8186 | Aspirin 300mg modified-release tablets |
| 20621 | Dicloflex 75mg SR tablets (Kent Pharmaceuticals Ltd) |
| 34738 | Naproxen 250mg gastro-resistant tablets (A A H Pharmaceuticals Ltd) |
| 20967 | Phorpain 5% gel (Mercury Pharma Group Ltd) |
| 50314 | Brufen 600mg effervescent granules sachets (Doncaster Pharmaceuticals Ltd) |
| 20978 | Anadin Ultra liquid capsules (Wyeth Consumer Healthcare) |
| 4806 | Voltarol 100mg suppositories (Novartis Pharmaceuticals UK Ltd) |
| 6226 | Aspirin 500mg / Papaveretum 7.71mg dispersible tablets sugar free |
| 917 | Diclofenac sodium 50mg tablets |
| 34977 | Naproxen 500mg Gastro-resistant tablet (Galen Ltd) |
| 14672 | Defanac 75mg SR tablets (Ranbaxy (UK) Ltd) |
| 28256 | Diclofenac 50mg Tablet (Berk Pharmaceuticals Ltd) |
| 53980 | Naproxen 250mg tablets (Phoenix Healthcare Distribution Ltd) |
| 45256 | Indometacin 25mg Capsule (Meridian Healthcare (UK) Ltd) |
| 3416 | Diclofenac sodium 100mg modified-release tablets |
| 40086 | Acoflam 50mg gastro-resistant tablets (Mercury Pharma Group Ltd) |
| 25211 | Anadin Original tablets (Pfizer Consumer Healthcare Ltd) |
| 39085 | Naproxen 250mg tablets (A A H Pharmaceuticals Ltd) |
| 156 | Diclofenac 1% gel |
| 22232 | Disprin Direct 300mg orodispersible tablets (Reckitt Benckiser Healthcare (UK) Ltd) |
| 29316 | Care ibuprofen 400mg Tablet (Thornton & Ross Ltd) |
| 43904 | Feminax Express 342mg tablets (Bayer Plc) |
| 43045 | Diclofenac potassium 50mg tablets (Actavis UK Ltd) |
| 416 | Ibuprofen 200mg tablets |
| 16474 | Nabumetone 500mg dispersible tablets sugar free |
| 45842 | Ibuprofen 600mg Tablet (Celltech Pharma Europe Ltd) |
| 21050 | Ketonal 100mg Capsule (Lagap) |
| 37587 | Etoricoxib 30mg tablets |
| 34757 | Ibuprofen 400mg Tablet (Unichem) |
| 20395 | Flamatak MR 75mg tablets (Actavis UK Ltd) |
| 34729 | Ibuprofen 400mg tablets (OBG Pharmaceuticals Ltd) |
| 14884 | Voltarol Gel Patch 1% medicated plasters (Novartis Consumer Health UK Ltd) |
| 39264 | Dicloflex Retard 100mg tablets (Dexcel-Pharma Ltd) |
| 13807 | Deep relief ibuprofen 5% Gel (Mentholatum Company) |
| 31870 | Aspirin 320mg tablets |
| 56736 | Aspirin 300mg tablets (Waymade Healthcare Plc) |
| 3492 | Diflunisal 500mg tablets |
| 28522 | Ibuprofen 200mg / Pseudoephedrine hydrochloride 30mg tablets |
| 15023 | Naproxen 375mg Modified-release tablet |
| 27968 | Apsifen 400mg Tablet (Approved Prescription Services Ltd) |
| 34438 | Mefenamic acid 250mg capsules (A A H Pharmaceuticals Ltd) |
| 34670 | Naproxen 250mg Gastro-resistant tablet (Galen Ltd) |
| 37553 | Ibucalm 400mg tablets (Aspar Pharmaceuticals Ltd) |
| 26631 | Rhumalgan XL 100mg capsules (Sandoz Ltd) |
| 112 | Ibuprofen 5% cream |
| 58071 | Voltarol Rapid 50mg tablets (Waymade Healthcare Plc) |
| 22776 | Aspirin 500mg with Cyclizine 25mg effervescent tablets |
| 31211 | Aspirin 75mg Dispersible tablet (A A H Pharmaceuticals Ltd) |
| 28888 | Galprofen Long Lasting 200mg capsules (Galpharm International Ltd) |
| 31482 | Apsifen 200mg Tablet (Approved Prescription Services Ltd) |
| 20650 | Aspirin 300mg / Paracetamol 200mg dispersible tablets sugar free |
| 28695 | Piroflam 10mg Capsule (Opus Pharmaceuticals Ltd) |
| 3077 | Oruvail 2.5% gel (Sanofi) |
| 20036 | Clotam 200mg Capsule (Thames Laboratories Ltd) |
| 41622 | Piroxicam 10mg capsules (A A H Pharmaceuticals Ltd) |
| 5896 | Ibuleve Maximum Strength 10% gel (Dendron Ltd) |
| 21123 | Piroxicam 20mg Capsule (Berk Pharmaceuticals Ltd) |
| 25361 | Diclovol 25mg gastro-resistant tablets (Arun Pharmaceuticals Ltd) |
| 41766 | Maximum Strength Aspro Clear 500mg effervescent tablets (Bayer Plc) |
| 46942 | Ibuprofen 600mg tablets (IVAX Pharmaceuticals UK Ltd) |
| 27901 | VOLTAROL RETARD |
| 2129 | Brufen retard tabs 800mg Modified-release tablet (Abbott Laboratories Ltd) |
| 13893 | Nurofen Plus tablets (Reckitt Benckiser Healthcare (UK) Ltd) |
| 46141 | Nurofen Tension Headache 342mg caplets (Reckitt Benckiser Healthcare (UK) Ltd) |
| 48138 | Ibuprofen 200mg tablets (Aspar Pharmaceuticals Ltd) |
| 392 | Ibuprofen 200mg modified-release capsules |
| 17165 | Nycopren 500mg gastro-resistant tablets (Ardern Healthcare Ltd) |
| 15159 | Tolfenamic acid 200mg Capsule |
| 2622 | Ibuprofen 800mg tablets |
| 3309 | Aspirin 325mg / Caffeine 15mg tablets |
| 35967 | Paramed Extra Power Pain Control tablets (Galpharm International Ltd) |
| 52389 | Voltarol 50mg suppositories (Sigma Pharmaceuticals Plc) |
| 23488 | Claradin 300mg Tablet (Nicholas Laboratories Ltd) |
| 34923 | Naproxen 250mg Tablet (Berk Pharmaceuticals Ltd) |
| 49862 | Voltarol 1% Emulgel (Lexon (UK) Ltd) |
| 32641 | Indometacin 25mg capsules (A A H Pharmaceuticals Ltd) |
| 407 | Brufen 600mg effervescent granules sachets (Abbott Laboratories Ltd) |
| 11907 | Dexibuprofen 400mg tablets |
| 3901 | Naprosyn EC 500mg tablets (Roche Products Ltd) |
| 28479 | Nurofen Back Pain SR 300mg capsules (Reckitt Benckiser Healthcare (UK) Ltd) |
| 27082 | Ketpron XL 100mg capsules (Mercury Pharma Group Ltd) |
| 25330 | Solpaflex tablets (GlaxoSmithKline Consumer Healthcare) |
| 41677 | Mefenamic acid 250mg Capsule (IVAX Pharmaceuticals UK Ltd) |
| 395 | Aspirin mixture |
| 1571 | Ketoprofen 100mg modified-release capsules |
| 657 | Aspirin 500mg granules sachets sugar free |
| 51829 | Naproxen 250mg tablets (Kent Pharmaceuticals Ltd) |
| 18364 | Ibular 400mg Tablet (Lagap) |
| 3 | Aspirin 75mg dispersible tablets |
| 45216 | Ibuprofen 400mg Tablet (C P Pharmaceuticals Ltd) |
| 23878 | Nu-seals cardio ec 75mg Gastro-resistant tablet (Genus Pharmaceuticals Ltd) |
| 25643 | Surgam 300mg Sachets (Sanofi) |
| 23795 | Imbrilon 100mg Suppository (Berk Pharmaceuticals Ltd) |
| 1766 | Voltarol sr 75mg Modified-release tablet (Novartis Pharmaceuticals UK Ltd) |
| 21921 | Postmi ec 300mg Gastro-resistant tablet (Ashbourne Pharmaceuticals Ltd) |
| 919 | Indometacin 100mg suppositories |
| 2234 | Nabumetone 500mg tablets |
| 51474 | Aspirin 150mg suppositories (Martindale Pharmaceuticals Ltd) |
| 35890 | Nurofen 200mg caplets (Reckitt Benckiser Healthcare (UK) Ltd) |
| 14541 | Ponstan 50mg/5ml paediatric Liquid (Chemidex Pharma Ltd) |
| 32916 | Diclofenac 75mg Modified-release capsule (Sandoz Ltd) |
| 34091 | Diclofenac sodium 25mg gastro-resistant tablets (Sandoz Ltd) |
| 46940 | Ketoprofen 100mg capsules (Generics (UK) Ltd) |
| 17750 | Indomax 25mg Capsule (Ashbourne Pharmaceuticals Ltd) |
| 39317 | Naproxen 500mg tablets (Wockhardt UK Ltd) |
| 7141 | Ibugel Forte 10% gel (Dermal Laboratories Ltd) |
| 24086 | Ibuprofen 5% gel (Thornton & Ross Ltd) |
| 10711 | Tolectin 400mg Capsule (Cilag Pharmaceuticals Ltd) |
| 9637 | Keral 25mg tablets (A. Menarini Farmaceutica Internazionale SRL) |
| 27783 | Ibuprofen 400mg tablets sugar coated (Actavis UK Ltd) |
| 17704 | Platet 100mg Effervescent tablet (Roche Products Ltd) |
| 57545 | Voltarol 1% Emulgel (Dowelhurst Ltd) |
| 10325 | Dexibuprofen 300mg tablets |
| 807 | Naproxen 500mg tablets |
| 10678 | Fenopron 300 tablets (Typharm Ltd) |
| 5455 | Etodolac 600mg modified-release tablets |
| 21150 | Strefen 8.75mg lozenges (Reckitt Benckiser Healthcare (UK) Ltd) |
| 40 | Diclofenac sodium 50mg gastro-resistant tablets |
| 51284 | Arcoxia 60mg tablets (Sigma Pharmaceuticals Plc) |
| 37053 | Migrafen 200mg tablets (Chatfield Laboratories) |
| 15930 | Ibuprofen 5% / Levomenthol 3% gel |
| 296 | Ponstan Forte 500mg tablets (Chemidex Pharma Ltd) |
| 11550 | Nurofen Meltlets 200mg tablets (Reckitt Benckiser Healthcare (UK) Ltd) |
| 16221 | Diclozip 25mg gastro-resistant tablets (Ashbourne Pharmaceuticals Ltd) |
| 15104 | Naproxen 500mg Granules |
| 15201 | Volraman 50mg gastro-resistant tablets (LPC Medical (UK) Ltd) |
| 9465 | Diclotard 100 100mg Modified-release tablet (Galen Ltd) |
| 434 | Aspirin 300mg gastro-resistant tablets |
| 7516 | Aspirin 300mg effervescent tablets sugar free |
| 4298 | Nurofen 200mg Tablet (Crookes Healthcare Ltd) |
| 7535 | Nurofen 200mg Capsule (Crookes Healthcare Ltd) |
| 29524 | Ibumetin 600mg Tablet (Alfred Benzon (UK) Ltd) |
| 23121 | Arthroxen 500mg Tablet (C P Pharmaceuticals Ltd) |
| 56503 | Asasantin Retard capsules (Mawdsley-Brooks & Company Ltd) |
| 31956 | Aspirin 75mg gastro-resistant tablets (Kent Pharmaceuticals Ltd) |
| 40083 | Ibuprofen 200mg caplets (Galpharm International Ltd) |
| 7058 | Calprofen 100mg/5ml Oral suspension (McNeil Products Ltd) |
| 10033 | Etodolac 300mg capsules |
| 54304 | Naproxen 500mg tablets (Actavis UK Ltd) |
| 53816 | Aspirin 300mg dispersible tablets (Alliance Healthcare (Distribution) Ltd) |
| 19007 | Naprosyn 500mg Granules (Roche Products Ltd) |
| 15364 | Aspirin 150mg suppositories |
| 38992 | Flamrase 75mg SR tablets (Teva UK Ltd) |
| 19046 | Ibuprofen 400mg tablets (A A H Pharmaceuticals Ltd) |
| 34359 | Ibuprofen 400mg tablets (Vantage) |
| 29455 | Flexotard MR 100mg tablets (Pfizer Ltd) |
| 47937 | Aspirin 75mg dispersible tablets (Wockhardt UK Ltd) |
| 27484 | Piroxicam 20mg/1ml solution for injection ampoules |
| 46925 | Co-codaprin 8mg/400mg dispersible tablets (Actavis UK Ltd) |
| 38527 | Proflex Pain Relief 5% cream (Novartis Consumer Health UK Ltd) |
| 19975 | Parecoxib 40mg powder for injection |
| 560 | Diflunisal 250mg tablets |
| 17131 | Lederfen 300mg tablets (Mercury Pharma Group Ltd) |
| 45331 | Ibuprofen 200mg Tablet (Co-Pharma Ltd) |
| 126 | Ponstan 250mg capsules (Chemidex Pharma Ltd) |
| 1688 | Indocid 50mg capsules (Merck Sharp & Dohme Ltd) |
| 30391 | Contraflam 500mg Tablet (Berk Pharmaceuticals Ltd) |
| 39873 | Cuprofen Maximum Strength 400mg tablets (SSL International Plc) |
| 33357 | Pacifene 200mg tablets (Sussex Pharmaceutical Ltd) |
| 45145 | Ibuleve Speed Relief Max Strength 10% gel (Dendron Ltd) |
| 1469 | Meloxicam 15mg tablets |
| 54526 | Aspirin 300mg tablets (Alliance Healthcare (Distribution) Ltd) |
| 38493 | Anadin Joint Pain 200mg tablets (Pfizer Consumer Healthcare Ltd) |
| 16918 | Flurbiprofen 0.03% eye drops 0.4ml unit dose preservative free |
| 40336 | Orudis 50mg capsules (Sanofi) |
| 51561 | Aspirin 75mg gastro-resistant tablets (Zanza Laboratories Ltd) |
| 48062 | Ibuprofen 200mg Tablet (Wockhardt UK Ltd) |
| 41615 | Indometacin 50mg Capsule (Approved Prescription Services Ltd) |
| 34616 | Piroxicam 0.5% gel (A A H Pharmaceuticals Ltd) |
| 21419 | Seractil 300mg tablets (Genus Pharmaceuticals Ltd) |
| 54463 | Diclofenac 50mg Tablet (Approved Prescription Services Ltd) |
| 16222 | Diclozip 50mg gastro-resistant tablets (Ashbourne Pharmaceuticals Ltd) |
| 45643 | Aspirin 75mg Soluble tablet (Celltech Pharma Europe Ltd) |
| 46638 | Paracetamol 500mg / Ibuprofen 200mg tablets |
| 20385 | Arthrosin 500 tablets (Ashbourne Pharmaceuticals Ltd) |
| 5268 | Naproxen 500mg modified-release tablets |
| 9688 | Diclovol 75mg SR tablets (Generics (UK) Ltd) |
| 7520 | Anadin Extra soluble tablets (Pfizer Consumer Healthcare Ltd) |
| 15286 | Ketocid 200 modified-release capsules (Chiesi Ltd) |
| 26165 | Diclofenac sodium 50mg gastro-resistant tablets (A A H Pharmaceuticals Ltd) |
| 56039 | Ibuprofen 600mg tablets (Waymade Healthcare Plc) |
| 34769 | Naproxen 500mg tablets (A A H Pharmaceuticals Ltd) |
| 3958 | Diclofenac 25mg suppositories |
| 50813 | Ibuderm 5% gel (Dermal Laboratories Ltd) |
| 39461 | Solpadeine Migraine Ibuprofen & Codeine tablets (Omega Pharma Ltd) |
| 23593 | PostMI 75 dispersible tablets (Ashbourne Pharmaceuticals Ltd) |
| 20805 | Dicloflex 75mg SR tablets (Teva UK Ltd) |
| 17068 | Nurofen Maximum Strength 10% gel (Reckitt Benckiser Healthcare (UK) Ltd) |
| 754 | Mobic 7.5mg suppositories (Boehringer Ingelheim Ltd) |
| 10913 | Diclofenac 0.1% eye drops |
| 32728 | Askit oral powder sachets (Bayer Plc) |
| 8451 | Etodolac 200mg Tablet |
| 4320 | Naprosyn 125mg/5ml oral suspension (Roche Products Ltd) |
| 34796 | Aspirin 75mg Gastro-resistant tablet (Galen Ltd) |
| 40253 | Ibuprofen 600mg Tablet (Sovereign Medical Ltd) |
| 649 | Diclofenac sodium 25mg gastro-resistant tablets |
