# Supplementary material for: The effect of glucocorticoid therapy on mortality in patients with rheumatoid arthritis and concomitant type II diabetes: a retrospective cohort study
Source: BMC Rheumatol. 2020 Feb 19;4:4. doi: 10.1186/s41927-019-0105-4 (PMC7029556; doi:10.1186/s41927-019-0105-4)
Supplement: Supplementary file 2 — Additional file 2. The RAE measure. [file 41927_2019_105_MOESM2_ESM.docx]

# The RAE measure

Suppose we wish to determine if the effect of treatment, $T$, on some outcome, $Y$, is different across patient subgroups defined by variable $M$, where $T$ and $M$ are binary. In a time-to-event data setting, the additive interaction effect is defined as the difference in risk differences across subgroups of$M$,

| ${INT}_{A}$ | $=\left( \lambda\left( l;T=1,M=1, X \right)-\lambda\left( l;T=0, M=1, X \right) \right)$  $- \left( \lambda\left( l;T=1,M=0, X \right)-\lambda\left( l;T=0, M=0, \right) \right)$ |
| --- | --- |

where$\lambda(l,\ldots)$ denotes the hazard function at time $l$. If this measure departs from 0, there is a difference in the hazard differences in the subgroups of M.

The most common model for time-to-event data is the Cox proportional hazards model. This is a semi-parametric model which incorporates a baseline hazard, $\lambda_{0}(l)$, which is a function of time and is not estimated. However, if we were to fit this model including the treatment, the moderator, interaction between the two and any additional covariates, $X_{k}$, $k=1,..,n$ (equation 1) we would not be able to directly estimate the additive interaction effect as a function of the estimated regression coefficients as this measure depends on the baseline hazard function $\lambda_{0}(l)$ (equation 2).

| $\lambda\left( l;T, X \right)=\lambda_{0}\left( l \right)e^{\beta_{0}+\beta_{1}T+\beta_{2}M+\beta_{3}TM+\sum_{k=1}^{n} c_{k}X_{k}}$ | ( 1 ) |
| --- | --- |

| $\hat{INT}_{A}$ | $={(\lambda}_{0}\left( l \right)e^{\hat{\beta}_{0}+\hat{\beta}_{1}T+\hat{\beta}_{2}M+\hat{\beta}_{3}TM+\sum_{k=1}^{n} \hat{c}_{k}X_{k}}-\lambda_{0}\left( l \right)e^{\hat{\beta}_{0}+\hat{\beta}_{2}M+\sum_{k=1}^{n} \hat{c}_{k}X_{k}})$  $- (\lambda_{0}\left( l \right)e^{\hat{\beta}_{0}+\hat{\beta}_{1}T+\sum_{k=1}^{n} \hat{c}_{k}X_{k}}-\lambda_{0}\left( l \right)e^{\hat{\beta}_{0}+\sum_{k=1}^{n} \hat{c}_{k}X_{k}})$  $=\lambda_{0}(l)e^{\sum_{k=1}^{n} \hat{c}_{k}X_{k}}(e^{\hat{\beta}_{1}+\hat{\beta}_{2}+\hat{\beta}_{3}}-e^{\hat{\beta}_{1}}-e^{\hat{\beta}_{2}}+1)$ | ( 2 ) |
| --- | --- | --- |

However, we can also compare the hazard differences in subgroups of $M$ by considering the ratio of the hazard differences; defined the Ratio of Absolute Effects ($RAE$) measure.

$$RAE=\frac{\lambda\left( l;T=1,M=1, X \right)-\lambda\left( l;T=0, M=1, X \right)}{\lambda\left( l;T=1,M=0, X \right)-\lambda\left( l;T=0, M=0, X \right)}$$

If this measure departs from 1, there is a difference in the hazard differences in the subgroups of M. An $RAE=a$ implies that the absolute treatment effect in patients with $M=1$ is $a$ times that in patients with $M=0$. An $RAE>1$ suggests either a larger positive absolute effect or a smaller negative effect in patients with $M=1$ compared to patients with $M=0$. If $RAE\approx1$, there is no suggestion of treatment effect modification by $M$ on the additive scale. Moreover if $RAE<0$, the estimated absolute treatment effect is in the opposite direction in the two subgroups.

When the $RAE$ is estimated as a function of the regression coefficients of the Cox model, the baseline hazard function cancels out; thus, unlike the additive interaction effect, the $RAE$ measure can be calculated from this model.

| $\frac{}{}\hat{RAE}$ | $=\frac{\lambda_{0}\left( l \right)e^{{\hat{\beta}_{0}+\hat{\beta}}_{1}+\hat{\beta}_{2}+\hat{\beta}_{3}+\sum_{k=1}^{n} \hat{c}_{k}X_{k}}-\lambda_{0}\left( l \right)e^{\hat{\beta}_{0}+\hat{\beta}_{2}+\sum_{k=1}^{n} \hat{c}_{k}X_{k}}}{\lambda_{0}\left( l \right)e^{{\hat{\beta}_{0}+\hat{\beta}}_{1}+\sum_{k=1}^{n} \hat{c}_{k}X_{k}}-\lambda_{0}\left( l \right)e^{\hat{\beta}_{0}+\sum_{k=1}^{n} \hat{c}_{k}X_{k}}}$  $=\frac{e^{\hat{\beta}_{1}+\hat{\beta}_{2}+\hat{\beta}_{3}}-e^{\hat{\beta}_{2}}}{e^{\hat{\beta}_{1}}-1}$ |
| --- | --- |

The standard error for this measure can be estimated using the delta method. As the $RAE$ is a ratio, it is unlikely to be normally distributed. Therefore, to obtain a 95% confidence interval for the $\hat{RAE}$, one can calculate$log(RAE)$ and it’s standard error, calculate a 95% confidence interval for $log(RAE)$ assuming it is approximately normally distriuted (95% CI: estimate $\pm1.95\times$ standard error) and then exponentiate the upper and lower limits.
